# Lower Limb Amputations Among Individuals Living With Diabetes Mellitus in Low‐ and Middle‐Income Countries: A Systematic Review and Meta‐Analysis

**DOI:** 10.1002/wjs.70493

**Published:** 2026-07-14

**Authors:** Kriste N. Alberts, Mercia N. Companie, Maxine P. Kerrod, Jessica Davies, Rosa D. E. Bock, Cinae Swart, Eyitayo O. Owolabi, Justine Davies, Kathryn M. Chu, Michael McCaul

**Affiliations:** ^1^ Department of Surgery Centre for Global Surgery Stellenbosch University Stellenbosch Cape Town South Africa; ^2^ Faculty of Medicine and Health Sciences Stellenbosch University Stellenbosch Cape Town South Africa; ^3^ International Association of Student Surgical Societies Stellenbosch Cape Town South Africa; ^4^ St John's Hospital NHS Lothian Lothian UK; ^5^ Center for Health Promotion and Disease Prevention Edson College of Nursing and Health Innovation Arizona State University Tempe Arizona USA; ^6^ Institute of Applied Health Research University of Birmingham Birmingham UK; ^7^ Division of Epidemiology and Biostatistics Department of Global Health Stellenbosch University Stellenbosch Cape Town South Africa

**Keywords:** diabetes mellitus, high‐risk, incidence, lower limb amputation, prevalence

## Abstract

**Background:**

Diabetes‐related lower‐limb amputation (LLA) is a major global health problem; although rates have declined in some high‐income settings, the burden in low‐ and middle‐income countries (LMICs) has not been systematically quantified. This review aimed to estimate the prevalence and incidence of diabetes‐related LLA in LMICs, based on available published evidence from community and facility‐based populations.

**Methods:**

Following a PROSPERO‐registered protocol (CRD42021238656), we searched Medline, CINAHL, AJOL, Scopus, Embase, and Google Scholar (January 1990–June 2025) for quantitative studies reporting diabetes‐related LLA in LMICs from community and facility populations. Data were extracted using Joanna Briggs Institute tools, low‐quality studies were excluded from quantitative synthesis, and meta‐analyses used the Freeman–Tukey double‐arcsine transformation; subgroup analyses (age, sex, WHO region, high‐risk groups) explored heterogeneity, and certainty of evidence (CoE) was assessed with GRADE.

**Results:**

Of 201 included studies from 32 LMICs, 121 moderate‐to‐high‐quality studies were meta‐analyzed; most were facility‐based high‐risk cohorts (especially diabetic foot or advanced‐complication care). Pooled prevalence was 23 per 100 individuals (95% CI 0.21–0.24; *n* = 89; moderate CoE), incidence 29 per 100 (95% CI 0.25–0.32; *n* = 29; low CoE), re‐amputation 31 per 100 (95% CI 0.14–0.48; *n* = 8; low CoE), and contralateral amputation 23 per 100 (95% CI 0.13–0.33; *n* = 2; very low CoE).

**Conclusions:**

Diabetes‐related LLA remains a substantial burden in LMICs, while scarce population‐based data highlights key surveillance gaps for prevention planning.

## Graphical abstract

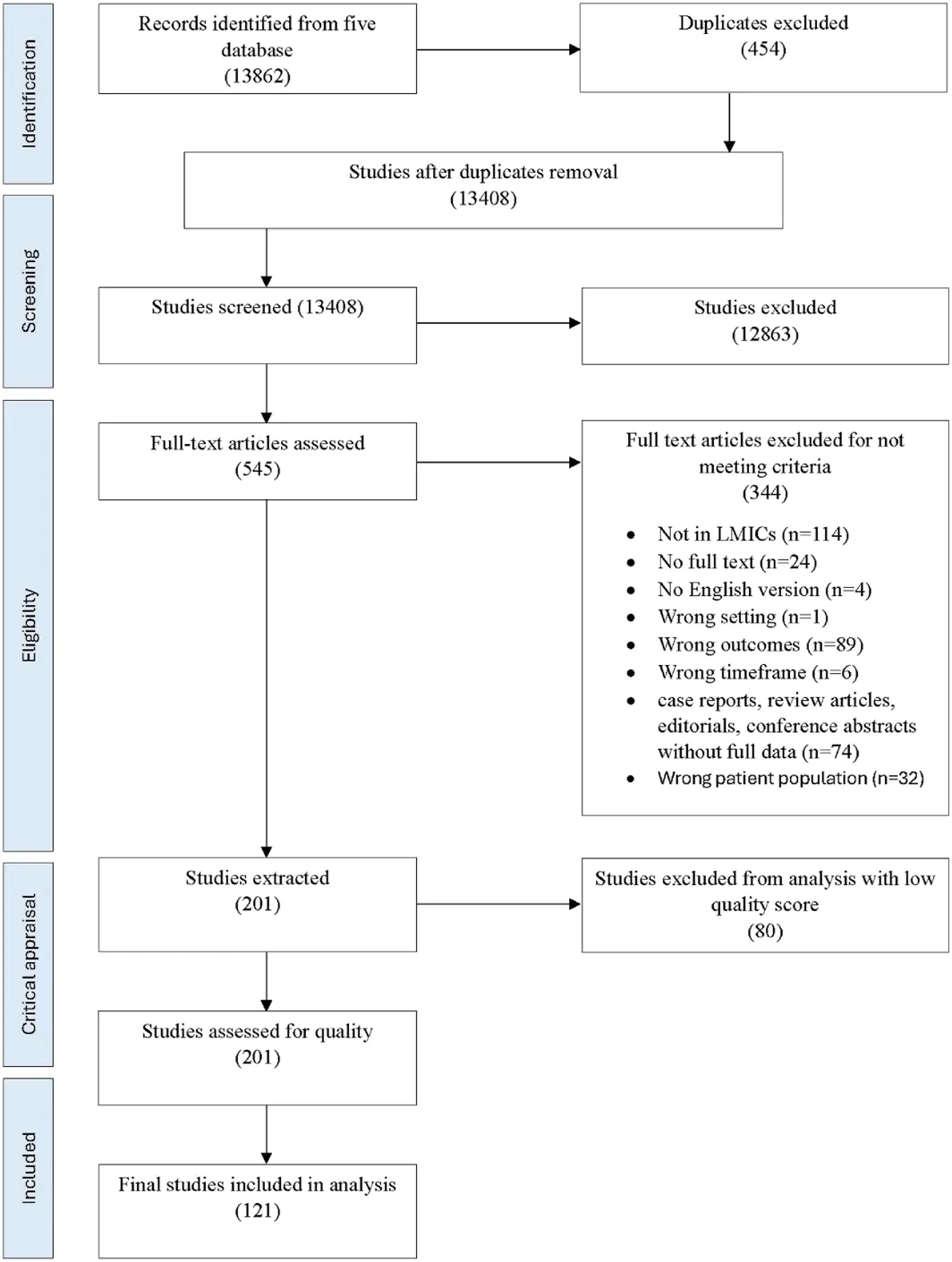

## 1 Introduction

Diabetes mellitus (DM) is a major global health challenge, with its burden increasing rapidly in low‐ and middle‐income countries (LMICs). The International Diabetes Federation estimates that nearly 80% of people living with diabetes reside in LMICs, where access to timely diagnosis and adequate disease management remains limited [1, 2]. As a result, diabetes‐related complications continue to rise, placing an increasing strain on already overburdened health systems.

Lower limb amputation (LLA) is one of the most severe and life‐altering complications of diabetes and contributes substantially to disability, loss of productivity, and premature mortality [3, 4, 5]. Diabetes‐related LLAs account for a large proportion of all non‐traumatic amputations globally and are widely regarded as a marker of advanced disease and the effectiveness of diabetes and foot‐care services within health systems [4, 6]. Each amputation not only reflects severe underlying pathology but also signals missed opportunities for earlier detection and prevention.

In some high‐income countries, reductions in diabetes‐related amputation rates have been reported, and have been attributed to earlier diagnosis, structured screening programmes, and the expansion of multidisciplinary foot‐care services [6, 7, 8]. In contrast, evidence from LMICs remains limited and heterogeneous. Health systems in these settings frequently face structural barriers—including restricted access to primary care, limited diagnostic capacity, and delayed referral pathways—that may contribute to an increased risk of preventable amputations [2, 9]. However, the extent of diabetes‐related LLAs across LMICs has not been comprehensively quantified.

Existing global reviews have primarily focussed on high‐income settings or have included LMICs without consistent methodological approaches to estimating incidence and prevalence, limiting comparability across regions and populations [10, 11]. The absence of systematically synthesized epidemiological data hampers any efforts to understand the burden of diabetes‐related LLA in LMICs and constrains health‐system planning, surveillance, and resource allocation. To address this evidence gap, this systematic review aims to estimate the incidence and prevalence of diabetes‐related LLA in LMICs using available published data, and to describe secondary outcomes, including contralateral amputations and re‐amputations.

## 2 Materials and Methods

This study was conducted in accordance with a previously published and peer‐reviewed protocol [11]; however, the data collection period was extended beyond the original timeframe to June 2025 to ensure inclusion of the most recent evidence.

### 2.1 Eligibility Criteria

We included studies that investigated LLA among individuals with DM in LMICs. We included any population irrespective of risk, across community and facility settings, including hospitals, specialist clinics, registries, and administrative databases. Eligible studies were required to report primary quantitative data on the incidence and/or prevalence of LLA within persons with diabetes typically from cross‐sectional, cohort or case‐control studies, as defined by the study authors, with or without associated risk factors or comorbid conditions.

When full texts were not available in English, articles were translated using Google Translate when feasible or accessed via university library networks.

We excluded case reports, review articles, editorials, conference abstracts without full data, and studies not reporting primary quantitative estimates of LLA incidence or prevalence among persons with diabetes.

### 2.2 Information Sources and Search Strategy

A comprehensive literature search was conducted across five electronic databases: Medline, CINAHL, AJOL, Scopus and Embase. Searches combined controlled vocabulary terms (where applicable) and free‐text keywords related to “lower limb amputation,” “diabetes,” and “low‐ and middle‐income countries,” using Boolean operators (AND/OR). The complete search strategies for each database are provided in Supporting Information S1: Online Resource 1 (ESM_1).

Searches were limited to studies published between January 1990 and June 2025. No language restrictions were applied during database searching.

In addition, a supplementary search was conducted using Google Scholar. As Google Scholar is not a formally indexed bibliographic database, a targeted approach was employed. Using the core search string, the first 10 pages of search results (sorted by relevance) were screened for potentially eligible studies. Reference lists of all included articles were also manually screened to identify any eligible studies not captured through database searches.

### 2.3 Study Selection

Titles and abstracts of all identified records were independently screened by seven reviewers (EO, CS, KA, MC, MK, JD, and RB) using the predefined eligibility criteria. Each article was screened by two independent reviewers. Screening assignments were randomly allocated using Covidence. Discrepancies at the title and abstract screening stage were resolved through discussion among the reviewers until consensus was reached.

Full texts of potentially eligible articles were retrieved and independently assessed for inclusion by five reviewers (KA, MC, MK, JD and RB). Full‐text screening assignments were randomly allocated using Covidence. Any disagreements were resolved through group discussion until consensus was reached.

The study selection process followed the Preferred Reporting Items for Systematic Reviews and Meta‐Analyses (PRISMA) 2020 guidelines, and a PRISMA‐compliant flow diagram was employed to document the selection process.

### 2.4 Data Extraction

Data from included studies were extracted using a standardized data extraction form, adapted from the Joanna Briggs Institute (JBI) template for prevalence and incidence reviews. Extracted variables included study title and authors, year of publication country, study design and setting, sample size, and population characteristics (e.g., age and sex). Data on the type and definition of amputation, as well as primary outcome measures (incidence and/or prevalence estimates of LLA) were also extracted.

Data extraction was conducted independently by five reviewers with studies distributed among reviewers (KA, MC, MK, JD and RB). Any inconsistencies identified during extraction were resolved through discussion until consensus was reached. All extracted data were compiled and managed using Microsoft Excel for subsequent analysis.

### 2.5 Critical Appraisal

The methodological quality of included studies was assessed using the JBI critical appraisal tools. For studies reporting prevalence outcomes, the JBI checklist for prevalence studies was applied [12]. For studies reporting incidence outcomes, the JBI checklist corresponding to the relevant study design was used (e.g., the JBI cohort checklist for cohort studies) [12].

Each study was independently appraised by two reviewers, with studies randomly allocated among four reviewers (KA, MC, MK and JD). Any discrepancies were resolved through discussion until consensus was reached. Studies were assessed across key methodological domains, including clarity of inclusion criteria, validity of outcome measurement, adequacy of sample size, appropriateness of analysis, and consideration of confounding.

For each study, an overall appraisal value reflecting the number of JBI criteria met was derived to facilitate broad categorization of methodological quality (e.g., low, moderate, or high). Studies with low methodological quality (JBI score ≤ 5), indicating the presence of multiple critical methodological limitations across key appraisal domains, were excluded from further quantitative synthesis.

### 2.6 Data Synthesis and Analysis

The primary outcomes were the incidence and/or prevalence of diabetes‐related LLA in LMICs. Incidence was defined as the number of new LLA cases among people with diabetes divided by the total diabetes population, while prevalence was defined as the total number of diabetes‐related LLAs in that population during a specified period [11]. Secondary outcomes included the incidence of contralateral amputation and re‐amputation. For each included study, incidence and/or prevalence proportions were derived from the reported numerators and denominators, where applicable.

All quantitative syntheses were performed in Stata, using the metaprop command for pooling proportions. Random‐effects meta‐analysis was performed using the DerSimonian–Laird method with the Freeman–Tukey double‐arcsine transformation to stabilize variances. Pooled estimates are reported with 95% confidence intervals, calculated using the Wilson score method.

Between‐study heterogeneity was assessed using Cochran's Q test and quantified with the *I* ^2^ statistic, with values of 0%–25% low, 25%–50% moderate, 50%–75% substantial, and > 75% considerable heterogeneity. Subgroup analyses (e.g., WHO region, income level, and study setting) and meta‐regression were undertaken where sufficient data were available (≥ 10 studies contributing to an analysis) to explore sources of heterogeneity.

Following quantitative synthesis, the certainty of evidence was assessed using the Grading of Recommendations Assessment, Development and Evaluation (GRADE) approach. Summary of Findings tables were prepared using GRADEpro. Certainty judgments considered risk of bias, inconsistency, indirectness, imprecision, and publication bias in accordance with the methods and recommendations provided in Chapter 14 of the Cochrane Handbook for Systematic Reviews of Interventions [13] and GRADE guidance [14]. Two review authors (KA and MM) independently assessed the certainty of evidence, with any disagreements resolved through discussion. Decisions to downgrade the certainty of the evidence were documented and justified in footnotes.

## 3 Results

### 3.1 Study Selection

**FIGURE 1:**
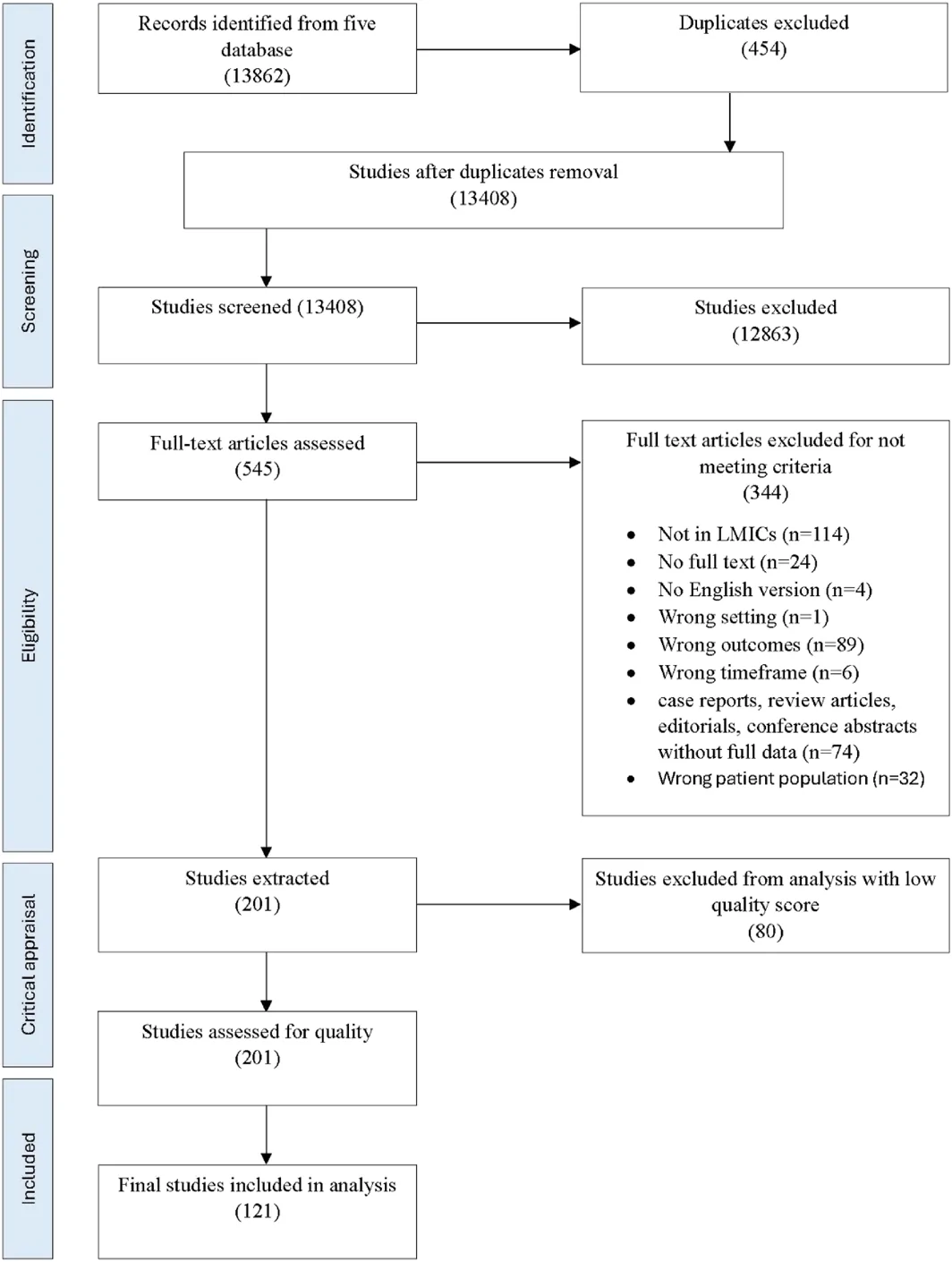
PRISMA flow diagram of included studies.

The database search identified 13,862 records, of which 454 duplicates were removed. The remaining 13,408 records were screened by title and abstract, and 12,863 records were excluded at this stage. A total of 545 full‐text articles were assessed for eligibility, of which 201 studies met the inclusion criteria and were included in the review (Figure 1). Non‐English full‐text articles that could not be accessed through university library resources were translated using Google Translate (*n* = 13).

### 3.2 Study Characteristics

A total of 201 studies met the inclusion criteria, following methodological quality appraisal, 111 studies were included for further analysis, representing data from 32 LMICs across 6 WHO regions. Studies were widely distributed across Low‐income (*n* = 16, 14.41%), Lower‐Middle Income (*n* = 40, 36.04%) and Upper Middle Income (*n* = 55, 49.55%) countries. Most studies were conducted in the South‐East Asia region (*n* = 29), followed by the Eastern Mediterranean region (*n* = 27) and Region of the Americas (*n* = 21), with fewer studies from the African Region (*n* = 17), Western Pacific (*n* = 11) and European (*n* = 6) regions.

Most included studies were cohort studies (56.76%), while a smaller proportion were cross‐sectional (45.04%). Sample sizes varied widely, ranging from 17 to over 5 million participants. The majority of studies were hospital‐based (79.27%), predominantly conducted in tertiary‐level (44.14%) or teaching (18.92%) hospitals.

Participant characteristics were heterogeneous. Reported mean ages ranged from 42 to 66 years, and sex distribution was generally balanced across studies. Several studies described populations with long‐standing diabetes and a high prevalence of comorbid conditions such as peripheral arterial disease, neuropathy, or infection, preceding amputation.

Definitions of LLA were generally consistent across studies and were based on surgical removal of the limb at or above the level described by the study authors.

Where reported, studies classified amputations as major, minor, or both, using author‐defined criteria; no attempt was made to reclassify amputation type using external or standardised definitions. Diabetes mellitus was identified according to the criteria reported by individual studies (e.g., clinical diagnosis or laboratory‐based measures), and reporting of diagnostic criteria was variable.

Included study populations were derived from healthcare and administrative data sources, including hospital inpatients (e.g., surgical, orthopedic, or medical wards), outpatient services, general diabetic clinics, specialist services such as diabetic foot clinics, and national or regional disease or disability registries.

Population‐based household surveys and community‐sampled cohorts were not identified in the available literature; consequently, all incidence and prevalence estimates were derived from individuals who had contact with health or social service systems rather than from population‐representative sampling frames.

The characteristics of all included studies are summarized in Supporting Information S2: Online Resource 2 (ESM_2).

### 3.3 Methodological Quality of Included Studies

Overall, the methodological quality of the included studies was considered predominantly moderate to high based on appraisal using the JBI critical appraisal tools. Of the 201 studies assessed, 80 were identified as having multiple critical methodological limitations and were excluded from further analysis, while 77 were categorized as moderate quality and 44 as high quality.

Across the included studies, several common methodological limitations were observed. Among the studies reporting prevalence outcomes, the most frequent limitations were related to inadequate sample size, non‐representative sampling frames, and lack of standardized measurement of the condition. In contrast, statistical analyses in these studies were generally of moderate quality.

For the studies reporting incidence outcomes, different JBI checklists were applied according to study design. Among cross‐sectional studies, common limitations included unclear inclusion criteria, inconsistent exposure measurement, and poor reliability of outcome measurement and statistical analysis. Cohort studies generally demonstrated higher adherence to quality appraisal criteria, particularly with respect to the identification and management of confounding factors and reliability of exposure measurement. No consistent differences in methodological quality were observed across regions or study designs.

### 3.4 Characteristics of Studies Contributing to Quantitative Synthesis

Following methodological quality appraisal, 111 studies contributed to the quantitative synthesis. Across these studies, the underlying study populations were predominantly high‐risk populations rather than general community‐based groups with diabetes. Accordingly, studies were classified as either high‐risk or general diabetes populations, and subgroup analyses by risk group are presented in Section 3.6; the specific high‐risk categories are summarized in Table 1.

**TABLE 1:** Baseline risk profile of included study populations.

Distribution of the 111 included studies according to study population category and baseline risk profile, showing the number and percentage of studies in general diabetes populations and high‐risk diabetes subgroups.		
Risk group	Study population category	Studies, *n* (%)
General diabetes population	All DM patients presenting to a facility (no pre‐specified foot complication/co‐morbidity required at enrollment)	26 (23.4)
High‐risk diabetes populations	All DFU/DFI or diabetic foot patients with DM	66 (59.5)
	All type 2 DM patients with DFU	13 (11.7)
	Other narrow subgroups defined by additional high‐risk features for example: • Participants identified as a disabled • Participants diagnosed with cranial neuropathy with a bacterial culture for drug resistance	6 (5.4)

Seventy‐nine studies focused specifically on individuals with diabetes‐related foot disease, including diabetic foot ulcers (DFU) or infections, while 26 studies included broader populations of individuals with diabetes presenting to healthcare facilities (clinic‐ or hospital‐based). A small number of studies (*n* = 6) examined narrowly defined subgroups, such as Type 1 DM only, Type 2 DM only, or persons with diabetes foot infections (DFI) defined by specific laboratory or clinical criteria; these were not disaggregated further due to heterogeneity and small numbers.

Although 111 unique studies contributed to the quantitative synthesis, a total of 121 outcome estimates were analyzed, as 10 studies reported more than one eligible outcome (e.g., prevalence and re‐amputation or contralateral amputation).

### 3.5 Prevalence and Incidence of Diabetes‐Related LLA

The pooled estimates for the prevalence and incidence of diabetes‐related LLA, as well as the incidence of re‐amputation and contralateral amputation, are presented in Table 2.

**TABLE 2:** Summary of findings for diabetes‐related lower limb amputation outcomes in LMICs.

Prevalence and incidence of diabetes‐related lower limb amputation, contralateral amputation, and re‐amputation among individuals with diabetes mellitus in low‐ and middle‐income countries from 1990 to 2025, with absolute effects and certainty of evidence assessed using GRADE.					
**Patient or population:** Individuals with diabetes mellitus receiving care in healthcare or service‐based settings in LMICs (predominantly higher‐risk clinical populations) and is at risk of or had LLA **Setting:** Population‐level and health facility‐level studies conducted in LMICs					
**Intervention:** Diabetes‐related LLA					
Outcomes	Absolute effects (95% CI)	№ of studies	№ of events	№ of individuals	Certainty of the evidence (GRADE)
Prevalence	23 per 100 (21–24)	89	55,666	5,999,142	⊕⊕⊕◯ Moderate^a,b,c^
Incidence	29 per 100 (25–32)	22	2509	29,385	⊕⊕◯◯ Low^a,b,d^
Incidence of contralateral amputation	23 per 100 (13–33)	2	18	70	⊕◯◯◯ Very low^a,b,e^
Incidence of Re‐amputation	31 per 100 (14–48)	8	221	1044	⊕⊕◯◯ Low^a,b,d^
**CI: Confidence interval** **Incidence estimates represent cumulative incidence proportions derived from reported numerators and denominators** **Grades of evidence:** **High certainty:** We are very confident that the true effect lies close to that of the estimate of the effect. **Moderate certainty:** We are moderately confident in the effect estimate: The true effect is likely to be close to the estimate of the effect, but there is a possibility that it is substantially different. **Low certainty:** Our confidence in the effect estimate is limited: The true effect may be substantially different from the estimate of the effect. **Very low certainty:** We have very little confidence in the effect estimate: The true effect is likely to be substantially different from the estimate of effect.					
**Explanations:** ^a.^ No serious risk of bias: Majority of studies were judged as moderate quality (JBI ROB tool) and low‐quality studies were excluded. ^b.^ serious inconsistency: Downgraded by 1 level due to significant unexplained clinical and statistical heterogeneity, despite subgroup analysis across age, sex, income‐region, year, clinical setting and comorbidities. ^c.^ No serious imprecision: Pooled 95% CI is clinically precise, we note the majority (∼72%) of studies included have a large sample size (> 1000). ^d.^ serious imprecision: Downgraded by 1 level for imprecision as the 95% CI of the pooled incidence includes a wide risk profile. ^e.^ very serious imprecision: Downgraded by 2 levels for imprecision as the 95% CI of the pooled incidence includes a wide risk profile, and we note the sample size is small (< 1000) and does not meet the minimum information size.					

Across 89 studies comprising 5,599,142 individuals and 55,666 diabetes‐related LLA events, the pooled prevalence of LLA among individuals with diabetes in LMICs was 23 per 100 individuals (95% CI 0.21–0.24), with substantial heterogeneity (*I* ^2^ = 93.71%, *p* < 0.001) as seen in Supporting Information S3: Online Resource 3 (ESM_3). The certainty of the evidence was rated as moderate and downgraded once for inconsistency.

Based on 22 studies including 29,385 participants and 2509 events, the pooled incidence proportion (risk) of diabetes‐related LLA was 29 per 100 individuals (95% CI 0.25–0.32). Heterogeneity was considerable (*I* ^2^ = 99.28%, *p* < 0.001), and the certainty of the evidence was low, downgraded for serious inconsistency and imprecision (Supporting Information S4: Online Resource 4 [ESM_4]).

Eight studies, comprising 1044 individuals and 221 events, reported on re‐amputation following diabetes‐related LLA. The pooled incidence was 31 per 100 individuals (95% CI 0.14–0.48), with high heterogeneity (*I* ^2^ = 98.42%, *p* < 0.001). The certainty of the evidence was low due to serious imprecision (Supporting Information S5: Online Resource 5 [ESM_5]).

Two studies, with 70 individuals and 18 contralateral amputation events, reported a pooled incidence proportion of 23 per 100 individuals (95% CI 0.13–0.33), with no observed heterogeneity (*I* ^2^ = 0.00%, *p* = 1.00; Supporting Information S6: Online Resource 6 [ESM_6]). The certainty of the evidence for this outcome was very low because of the small sample size and wide confidence intervals that did not meet the minimum information size.

### 3.6 Subgroup Analyses

#### 3.6.1 Prevalence

Subgroup analyses for prevalence are presented in Table 3. Pooled prevalence estimates were broadly similar across age groups, sex, and WHO regions, with no clear between‐group differences observed. Differences were observed by country income status, with lower pooled prevalence in lower‐middle‐income countries than in low‐ and upper‐middle‐income countries. However, substantial heterogeneity persisted within the subgroups (*I* ^2^ > 95%), reflecting continued variability across studies. In contrast, studies that included high‐risk populations characterized by foot‐related comorbidities (such as DFU) demonstrated higher pooled prevalence estimates of LLA than studies from general‐DM population groups. However, substantial within‐group heterogeneity remained in both subgroups (*I* ^2^ > 95%), indicating considerable variability in prevalence estimates across studies even within these categories.

**TABLE 3:** Subgroup analysis of pooled prevalence estimates for diabetes‐related lower limb amputation in LMICs.

Pooled prevalence estimates of diabetes‐related lower limb amputation stratified by age, sex, WHO region, country income status, and baseline risk profile, with within‐group heterogeneity and *p*‐values for between‐group differences.			
Subgroup	Pooled prevalence (95% CI)	*I* ^2^ (%)	Heterogeneity *p*‐value (between groups)
Overall	0.23 (0.21–0.24)	99.92	—
Age	< 50 years: 0.21 (0.16–0.27)	—	0.643
	≥ 50 years: 0.18 (0.12–0.24)	99.92	
Sex	Male‐dominant: 0.24 (0.23–0.26)	99.84	0.053
	Female‐dominant: 0.24 (0.21–0.26)	99.96	
WHO region	African: 0.19 (0.02–0.35)	99.98	0.233
	South‐East Asia: 0.21 (0.18–0.25)	99.74	
	Eastern Mediterranean: 0.26 (0.22–0.29)	99.88	
	Western Pacific: 0.19 (0.15–0.23)	92.37	
	Region of the Americas: 0.22 (0.19–0.24)	99.86	
	European: 0.27 (0.16–0.37)	95.56	
Country income status	Low income: 0.30 (0.06–0.55)	99.98	< 0.001
	Lower‐middle income: 0.16 (0.14–0.18)	99.65	
	Upper‐middle income: 0.23 (0.21–0.24)	99.77	
Risk‐profile	Identified high‐risk group: 0.28 (0.24–0.32)	99.74	< 0.005
	General DM population: 0.12 (0.09–0.15)	99.97	

#### 3.6.2 Incidence

Subgroup analyses for incidence are presented in Table 4. Pooled incidence estimates were broadly similar across age groups, with no clear between‐group differences observed. Variation in pooled incidence estimates was observed across sex, WHO region, country income status, and high‐risk versus general‐DM population groups, with higher pooled incidence estimates generally observed in studies of populations characterized as high‐risk compared with those in general‐DM population groups. Substantial within‐group heterogeneity persisted across all subgroups (*I* ^2^ > 95%), indicating considerable variability between studies.

**TABLE 4:** Subgroup analysis of pooled incidence estimates for diabetes‐related lower limb amputation.

Pooled incidence estimates of diabetes‐related lower limb amputation stratified by age, sex, WHO region, country income status and baseline risk profile, with within‐group heterogeneity and *p*‐values for between‐group differences.			
Subgroup	Pooled prevalence (95% CI)	*I* ^2^ (%)	Heterogeneity *p*‐value (between groups)
Overall	0.29 (0.25–0.32)	99.28	—
Age	< 50 years: 0.30 (0.23–0.39)	—	0.729
	≥ 50 years: 0.29 (0.25–0.32)	99.30	
Sex	Male‐dominant: 0.32 (0.27–0.38)	99.38	< 0.005
	Female‐dominant: 0.17 (0.10–0.23)	98.32	
WHO region	African: 0.22 (0.18–0.26)	99.01	< 0.005
	South‐East Asia: 0.33 (0.23–0.43)	97.18	
	Eastern Mediterranean: 0.28 (0.22–0.34)	—	
	Western Pacific: No studies	—	
	Region of the Americas: 0.20 (0.14–0.26)	98.22	
	European: 0.58 (0.50–0.66)	—	
Country income status	Low income: 0.35 (0.23–0.48)	—	< 0.001
	Lower‐middle income: 0.16 (0.11–0.20)	97.91	
	Upper‐middle income: 0.35 (0.26–0.45)	99.52	
Risk‐profile	Identified high‐risk group: 0.36 (0.24–0.47)	99.10	< 0.005
	General DM population: 0.12 (0.09–0.15)	98.41	

Subgroup analyses were not performed for the incidence of contralateral amputation and re‐amputation due to the limited number of studies (two and eight studies, respectively), which precluded reliable estimation of between‐group heterogeneity.

## 4 Discussion

Across studies conducted in LMIC settings, this systematic review found a high pooled prevalence and incidence of diabetes‐related LLA in high‐risk individuals, accompanied by substantial between‐study heterogeneity. In particular, the pooled estimates in this review are higher than global and population‐based incidence benchmarks reported for all people with diabetes in broader denominator studies (e.g., global annual incidence estimates in the order of ∼95 major and ∼140 minor amputations per 100,000 people with diabetes), highlighting the concentration of risk within advanced‐disease and facility‐attending cohorts [15].

The relatively high prevalence and incidence estimates observed in this review should be interpreted considering the underlying study populations. Most included studies were persons with high‐risk DM, particularly individuals attending diabetic foot clinics, hospital services, or receiving care for advanced diabetes‐related complications. These populations represent higher‐risk groups with an elevated probability of LLA compared with general community‐based groups with diabetes. Accordingly, the pooled estimates reported here do not represent population‐wide incidence or prevalence among all people with diabetes in LMICs but instead reflect the burden of diabetes‐related LLA among individuals who have already engaged with healthcare services and are at increased risk of severe outcomes.

Significant clinical and methodological variation is common in broad prevalence/incidence reviews and contributes to high *I* ^2^ values (often exceeding 90%) [16]. In this review, heterogeneity likely reflects differences in: (i) the event definition (major vs. minor; diabetes‐related vs. all‐cause; first amputation per person vs. multiple amputations), (ii) the denominator (general diabetes population vs. high‐risk groups), and (iii) measurement/ascertainment (clinical records, administrative data, registry‐based approaches), alongside variation in case‐mix severity and care context. These findings emphasize the need for further primary epidemiological studies in LMICs that use standardised case definitions, denominator specification, and consistent outcome ascertainment to improve comparability of occurrence measures across settings [16].

When contextualized against evidence from broader denominator studies (primarily from high‐income settings), the magnitude of diabetes‐related LLA observed aligns with expectations once denominators and care contexts are accounted for. Population‐based systematic reviews report incidence in people with diabetes ranging roughly from ∼78 to ∼704 per 100,000 person‐years (with additional variability by amputation definition and counting approach), and global estimates place annual diabetes‐related amputation incidence around ∼95 major and ∼140 minor per 100,000 people with diabetes [8]. In contrast, studies focused on advanced diabetic foot disease often report higher risks (e.g., meta‐analytic estimates suggesting an overall amputation proportion around ∼31% among patients with diabetic foot ulcers), which more closely resembles the high‐risk clinical cohorts represented in this review [17]. These comparisons reinforce that the elevated estimates in this review primarily reflect a high‐risk population of individuals living with diabetes in LMICs.

Subgroup patterns by sex, region, and high‐risk status were observed, but these findings were inconsistent across settings and should be interpreted as exploratory. Higher incidence estimates among males may reflect differences in underlying case‐mix, risk‐factor distribution, and access to timely preventive and limb‐salvage care rather than stable subgroup effects across LMIC contexts [8].

This review highlights important gaps in the current evidence base. Population‐based data on diabetes‐related LLA in LMICs remain scarce. Evidence on re‐amputation and contralateral amputation was limited, with few studies reporting these outcomes and wide confidence intervals around pooled estimates, limiting inference about disease course and recurrence patterns in LMIC settings. Future epidemiological studies should routinely report glycemic control (HbA1c), diabetes duration, vascular disease, infection severity, comorbidities, and reasons for re‐amputation using standardised definitions to enable future evidence synthesis and identification of modifiable risk factors.

Prior systematic reviews have largely focused on (i) population‐based incidence comparisons between people with and without diabetes (mostly in higher‐income settings) and (ii) global incidence estimates, while other work has examined related but distinct outcomes such as amputation occurrence among diabetic foot ulcer cohorts or the contribution of diabetes to amputations in specific regions [8]. By synthesizing this evidence, this review adds clarity on where the current evidence is concentrated, quantifies the burden in those who are most vulnerable, and delineates the key evidence gaps—particularly the lack of population‐representative occurrence data and limited reporting of re‐amputation/contralateral outcomes—thereby helping to prioritize future LMIC epidemiological surveillance and standardised measurement.

## 5 Strengths and Limitations in Context

This review synthesized over 3 decades of LMIC evidence using extensive database and manual reference searching, JBI appraisal, random‐effects meta‐analysis, and GRADE certainty assessment. Studies judged to be of low methodological quality were excluded from the quantitative synthesis, strengthening the internal validity of the pooled estimates. Including studies from multiple regions and healthcare settings provides a broad overview of diabetes‐related LLA in LMIC contexts.

Key limitations relate to study design and reporting. Most studies were retrospective, facility‐based and included high‐risk clinical cohorts, so estimates may be higher than in general diabetes populations and are not widely generalizable. High heterogeneity likely reflects differences in study populations, sampling, outcome definitions, and ascertainment methods. Follow‐up was often poorly reported, population‐representative data were limited, and evidence on contralateral amputation and re‐amputation was scarce, reducing precision for these outcomes.

## 6 Future Research

Future LMIC research should prioritize population‐representative, multi‐center studies that estimate diabetes‐related LLA incidence and prevalence using standardised definitions and clearly specified denominators. Studies should report follow‐up consistently and be long enough to capture incident amputations, re‐amputation, and contralateral amputation.

## 7 Conclusion

Diabetes‐related LLA remains a major and largely preventable cause of disability in LMICs. This review shows a substantial burden among people with diabetes accessing healthcare, although estimates vary across settings because of differences in case‐mix, definitions, and health‐system context. As most evidence comes from high‐risk, facility‐based cohorts, pooled estimates should not be interpreted as population‐wide risk for all people with diabetes in LMICs. These findings highlight the need for earlier detection, stronger diabetic foot care pathways, and better population‐representative data, consistent outcome definitions, and longitudinal follow‐up to guide prevention.

## Author Contributions

**Kriste N. Alberts:** conceptualization, investigation, writing – original draft, writing – review and editing, visualization, validation, methodology, formal analysis, project administration, data curation, supervision. **Mercia N. Companie:** writing – original draft, writing – review and editing, data curation, formal analysis, visualization, investigation. **Maxine P. Kerrod:** writing – original draft, writing – review and editing, visualization, formal analysis, data curation, investigation. **Jessica Davies:** writing – original draft, writing – review and editing, visualization, formal analysis, data curation, investigation. **Rosa D. E. Bock:** writing – review and editing, data curation, visualization, investigation. **Cinae Swart:** data curation, writing – review and editing. **Eyitayo O. Owolabi:** conceptualization, methodology, validation, investigation, writing – review and editing, data curation, supervision, project administration, software, visualization. **Justine Davies:** conceptualization, investigation, writing – review and editing, methodology, validation, supervision, visualization. **Kathryn M. Chu:** conceptualization, investigation, supervision, writing – review and editing, methodology, validation, visualization, writing – original draft. **Michael McCaul:** conceptualization, investigation, formal analysis, software, supervision, writing – original draft, writing – review and editing, methodology, validation, visualization.

## Funding

The authors have nothing to report.

## Conflicts of Interest

Michael McCaul is the co‐director of the South African GRADE network. There are no other conflicts of interest to declare.

## Supporting information


Supporting Information S1

Supporting Information S2

Supporting Information S3

Supporting Information S4

Supporting Information S5

Supporting Information S6

## Data Availability

The data that support the findings of this study are available from the corresponding author upon reasonable request.
