# Supplementary material for: Lower Limb Amputations Among Individuals Living With Diabetes Mellitus in Low‐ and Middle‐Income Countries: A Systematic Review and Meta‐Analysis
Source: World J Surg. 2026 Jul 14;50(9):2750–9. doi: 10.1002/wjs.70493 (PMC13576533; doi:10.1002/wjs.70493)
Supplement: Supplementary file 2 [file WJS-50-2750-s002.pdf]

World Journal of Surgery

Title: Lower limb amputations among individuals living with diabetes mellitus in low- and middle-income countries: A systematic review and meta-analysis

Short title: Diabetes-related LLA in LMICs: A systematic review and meta-analysis

Authors

Kriste N. Alberts[a*] (0000-0002-9068-6979), Mercia N. Companie[a] (0009-0007-0015-296X), Maxine P. Kerrod[b] (0009-0003-2801-288X), Jessica Davies[c] (0000-0002-4695-9028), Rosa D.E. Bock[d] (0009-0008-4735-008X), Cinae Swart[b] (0009-0001-7864-7467), Eyitayo O. Owolabi[e, a] (0000-0001-5575-7842), Justine Davies[f] (0000-0001-6834-1838), Kathryn M. Chu[a] (0000-0002-8923-7447), Michael McCaul[g] (0000-0002-2730-6478)

Affiliations

[a] Centre for Global Surgery, Department of Surgery, Stellenbosch University, Stellenbosch, Cape Town, South Africa

[b] Faculty of Medicine and Health Sciences, Stellenbosch University, Stellenbosch, Cape Town, South Africa

[c] International Association of Student Surgical Societies, Cape Town, South Africa

[d] St John's Hospital, NHS Lothian, United Kingdom

[e] Center for Health Promotion and Disease Prevention, Edson College of Nursing and Health Innovation, Arizona State University, Arizona, USA.

[f] Institute of Applied Health Research, University of Birmingham, Birmingham, United Kingdom

[g] Division of Epidemiology and Biostatistics, Department of Global Health, Stellenbosch University, Stellenbosch, Cape Town, South Africa

Diabetes-related LLA in LMICs: A systematic review and meta-analysis

***Corresponding author**

Kriste N. Alberts

Email address: kristealberts@sun.ac.za

Physical address: Room 4046A, 4th Floor, Education building, Francie van Zijl Drive, Parrow, Cape Town, 7500

Contact number: +27 21 938 9442

Online Resource 2 (ESM_2)

Detailed characteristics of all included studies, including article title, authors, publication year, study design, study period, JBI quality score, population description, age and sex distribution, outcome data, and study setting. For each study, the file also summarises the population at risk, number of amputations, country, WHO region, and level of care or data source.

Prevalence Studies	
[1]	**Title:** Admission Trends Over 8 Years for Diabetic Foot Ulceration in a Specialized Diabetes Unit in Cameroon<br>**Authors:** Kengne AP, Djouogo CFT, Dehayem MY, et al.<br>• *Publication Year:* 2009<br>• *Study Design:* Prospective Cohort<br>• *Study Period:* January 2000 - December 2007<br>• *JBI Quality Score:* Moderate (6)<br>**Population:**<br>The study population included all patients with diabetes admitted to the Endocrine and Diabetes Unit of Yaoundé Central Hospital in Cameroon with a diagnosis of DFU between January 2000 and December 2007.<br>• *Mean Age (SD):* 57.3 (14.5)<br>• *Male, %(n):* 56.6% (1042)<br>**Outcome:**<br>The study reported eight-year trends in DFU admissions, hospital bed use, and outcomes at a specialized diabetes care unit in Yaoundé, Cameroon.<br>• *Population at risk:* 240<br>• *Total no. of amputations:* 38

	**Setting:**<br>*Location:* Cameroon, African Region<br>*Study setting and level:* Hospital based – Level Not Specified
[2]	**Title:** Assessing the Knowledge of Patients With Diabetes About Foot Care and Prevention of Foot Complications in Cameroon, West Africa<br>**Authors:** C.K. Brown, C. Kejeh, C. Limnyuy, et al.<br>• *Publication Year:* 2025<br>• *Study Design:* Prospective Cohort<br>• *Study Period:* 23 December 2021 - 26 August 2022<br>• *JBI Quality Score:* Moderate (7)<br>**Population:**<br>The study population included patients with diabetes ≥21 years seen by wound care nurses at two hospitals who could communicate orally; excluded <21, cognitively impaired, unable to communicate, employees, or groups of persons without diabetes.<br>• *Mean Age (SD):* 59.2 (14.4)<br>• *Male, %(n):* 48.46% (63)<br>**Outcome:**<br>The study surveyed persons with diabetes in Cameroon to assess their foot-care knowledge and prevention practices, with the goal of improving diabetic foot education across a hospital system.<br>• *Population at risk:* 130<br>• *Total no. of amputations:* 15<br>**Setting:**<br>*Location:* Cameroon, African Region<br>*Study setting and level:* Hospital based - Multiple
[3]	**Title:** Prevalence and Determinants of Diabetic Foot Ulcers and Lower Extremity Amputations in Three Selected Tertiary Hospitals in Ghana<br>**Authors:** A. Atosona, C. Larbie<br>• *Publication Year:* 2019<br>• *Study Design:* Prospective Cross-Sectional<br>• *Study Period:* June 2015 - July 2015<br>• *JBI Quality Score:* Moderate (7)<br>**Population:**<br>The study population included persons with diabetes booked and attending clinic on a specified day with WHO-defined diabetes, on diet or antidiabetic drugs ≥1 year, age ≥18, and consented; excluded very ill and pregnant patients.<br>• *Mean Age (SD):* 53.8 (13.8)<br>• *Male, %(n):* 31% (31)<br>**Outcome:**

	The study defined the extent, associated risk factors, and predictors of foot ulcers and lower-extremity amputations (LEA) among persons with Type 1 and Type 2 DM in Ghana to support preventive strategies.<br>• *Population at risk:* 100<br>• *Total no. of amputations:* 3<br>**Setting:**<br>*Location:* Ghana, African Region<br>*Study setting and level:* Hospital based - Tertiary level
[4]	**Title:** Foot complications in people with diabetes: experience with 150 Nigerian Africans<br>**Authors:** Adigun I, and Olarinoye J<br>• *Publication Year:* 2008<br>• *Study Design:* Prospective Cross-Sectional<br>• *Study Period:* October 2003 - December 2003<br>• *JBI Quality Score:* Moderate (7)<br>**Population:**<br>The study population consisted of individuals aged 15 years and above with diabetes attending the outpatient clinic at the University of Ilorin Teaching Hospital, Nigeria, who provided informed consent between October and December 2003.<br>• *Mean Age (SD):* 56.78 (13.04)<br>• *Male, %(n):* 42.85% (45)<br>**Outcome:**<br>The study assessed the prevalence and types of diabetic foot complications and their management, measuring the incidence of foot ulcer and amputation.<br>• *Population at risk:* 105<br>• *Total no. of amputations:* 6<br>**Setting:**<br>*Location:* Nigeria, African Region<br>*Study setting and level:* Hospital based - Tertiary level
[5]	**Title:** Predictors of Lower Extremity Amputation Among Patients with Diabetic Foot Ulcer in a Tertiary Health Facility in North Central Nigeria<br>**Authors:** M. Enamino, G. Odoh, J.N Uwakwe, et. al<br>• *Publication Year:* 2018<br>• *Study Design:* Retrospective Cohort<br>• *Study Period:* April 2016 – April 2017<br>• *JBI Quality Score:* Moderate (7)<br>**Population:**<br>The study population included adult inpatients aged 20–80 years with DFUs treated at the Federal Medical Centre during the study period.

<table>
<tr><td></td><td>
Mean Age: Not reported
Male, %(n): 52.85% (37)
Outcome:
The study determined the prevalence and independent predictors of LEA among DFU patients at a tertiary health facility in North Central Nigeria.
Population at risk: 70
Total no. of amputations: 27
Setting:
Location: Nigeria, African Region
Study setting and level: Hospital based - Tertiary level
</td></tr>
<tr><td>[6]</td><td>
Title: Diabetic foot lesions as seen in Nigerian Teaching Hospital: Pattern and Simple Classification.
Authors: A. Musa
Publication Year: 2012
Study Design: Prospective Cohort
Study Period: 1 January 2012 - 31 December 2015
JBI Quality Score: Moderate (6)
Population:
The study population included persons with diabetes presenting with foot complaints to the diabetic foot clinic.
Mean Age: Not reported
Male, %(n): 62.22% (28)
Outcome:
The study examined patterns of diabetic foot lesions over a five-year period at the Usmanu Danfodiyo University Teaching Hospital in Nigeria and presented a classification of these lesions.
Population at risk: 45
Total no. of amputations: 7
Setting:
Location: Nigeria, African Region
Study setting and level: Hospital based - Teaching Hospital
</td></tr>
<tr><td>[7]</td><td>
Title: Predictors of lower extremity amputation in patients with diabetic foot ulcer: findings from MEDFUN, a multi-centre observational study
Authors: E. Ugwu, O. Adeleye, I. Gezawa, et. al.
Publication Year: 2019
Study Design: Prospective Cohort
Study Period: March 2016 - April 2017
JBI Quality Score: Moderate (7)
Population:
</td></tr>
</table>

	The study population included consenting adults (≥18) with Type 1 or Type 2 DM hospitalized for DFU and managed by a multidisciplinary team until discharge or death.<br>• *Mean Age (SD):* 55.9 (12.5)<br>• *Male, %(n):* 55.05% (185)<br>**Outcome:**<br>The study identified demographic, clinical, and laboratory variables that significantly predict LEA among patients hospitalized for DFUs.<br>• *Population at risk:* 336<br>• *Total no. of amputations:* 119<br>**Setting:**<br>*Location:* Nigeria, African Region<br>*Study setting and level:* Hospital based - Tertiary level
[8]	**Title:** Clinical and progressive profile of skin and soft tissue lesions in diabetics in 2017 at the dressing room of the Marc Sankale Center in Dakar<br>**Authors:** D.I. Mané, D. Demba, S. Djiby, et al.<br>• *Publication Year:* 2019<br>• *Study Design:* Prospective Cross-Sectional<br>• *Study Period:* 1 January 2017 - 31 December 2017<br>• *JBI Quality Score:* High (9)<br>**Population:**<br>The study population comprised persons with diabetes examined at the Marc Sankale Center, excluding those without records or without telephone contact information.<br>• *Mean Age (SD):* 56.6 (12)<br>• *Male, %(n):* 46.80% (7685)<br>**Outcome:**<br>The study determined the clinical and evolutionary profile of skin and soft tissue lesions in persons with diabetes followed up at the dressing room, describing lesion types, outcomes, and factors linked to amputation.<br>• *Population at risk:* 16418<br>• *Total no. of amputations:* 13259<br>**Setting:**<br>*Location:* Senegal, African Region<br>*Study setting and level:* Hospital based - Specialist
[9]	**Title:** Audit of Public Sector Primary Diabetes Care in Cape Town, South Africa: High Prevalence of Complications, Uncontrolled Hyperglycaemia, and Hypertension<br>**Authors:** NS. Levitt, D. Bradshaw, MF. Zwarenstein, et. al<br>• *Publication Year:* 1997<br>• *Study Design:* Prospective Cross-Sectional

	• *Study Period:* July 1992 - December 1992<br>• *JBI Quality Score:* High (9)<br>**Population:**<br>The study population consisted of patients attending community health centres.<br>• *Mean Age (SD):* 56.4 (11.2)<br>• *Male, %(n):* 38.27% (93)<br>**Outcome:**<br>The study investigated the prevalence of diabetes-related complications and assessed glycaemic and blood pressure control among Black African patients at primary care level in Cape Town, South Africa.<br>• *Population at risk:* 243<br>• *Total no. of amputations:* 3<br>**Setting:**<br>*Location:* South Africa, Arican Region<br>*Study setting and level:* Community - Primary Level
[10]	**Title:** Clinical outcome of diabetes patients hospitalized with foot ulcers, Dar es Salaam, Tanzania<br>**Authors:** Gulam-Abbas Z, Lutale JK, Morbach S., et al.<br>• *Publication Year:* 2002<br>• *Study Design:* Prospective Cohort<br>• *Study Period:* January 1997 - December 1998<br>• *JBI Quality Score:* High (9)<br>**Population:**<br>The study population comprised consecutive adult patients with diabetes who were newly hospitalized with foot ulcers.<br>• *Mean Age (SD):* 53 (21-90)<br>• *Male, %(n):* 65% (60)<br>**Outcome:**<br>The study determined the prevalence, clinical features, risk factors, and clinical outcomes of foot ulcers among hospitalized diabetes patients in Dar es Salaam, Tanzania.<br>• *Population at risk:* 627<br>• *Total no. of amputations:* 30<br>**Setting:**<br>*Location:* Tanzania, Arican Region<br>*Study setting and level:* Hospital based - Teaching Hospital
[11]	**Title:** Prevalence of diabetic foot disorders and related risk factors among Egyptian subjects with diabetes<br>**Authors:** S.H. Assaad-Khalil, A. Zaki b, A. Abdel Rehim

	• *Publication Year:* 2014<br>• *Study Design:* Prospective Cross-Sectional<br>• *Study Period:* July 2008 - December 2009<br>• *JBI Quality Score:* High (9)<br>**Population:**<br>The study population included attendants of the Diabetic Foot Screening Clinic in Alexandria; all general diabetes clinic patients were appointed to the DF clinic without prior selection.<br>• *Mean Age (SD):* 57.30 (10.47)<br>• *Male, %(n):* 50% (1000)<br>**Outcome:**<br>The study examined the prevalence of diabetic foot problems and their associated risk factors among patients in Egypt.<br>• *Population at risk:* 2000<br>• *Total no. of amputations:* 88<br>**Setting:**<br>*Location:* Egypt, Eastern Mediterranean Region<br>*Study setting and level:* Hospital based - Teaching Hospital
[12]	**Title:** The prevalence and risk categorization of diabetic foot complications in cohort group in, Beni Suif, Egypt, 2010-2012<br>**Authors:** N. Saad, K. Elhadedy, N. Ramadan, et al.<br>• *Publication Year:* 2013<br>• *Study Design:* Prospective Cross-Sectional<br>• *Study Period:* October 2010 - October 2012<br>• *JBI Quality Score:* Moderate (6)<br>**Population:**<br>The study population included Egyptian adults (500 males, 500 females) aged 20–80 years with Type 2 DM (ADA 2011), both inpatients and outpatients; traumatic foot complications excluded.<br>• *Mean Age (SD):* 57 (6)<br>• *Male, %(n):* 5% (500)<br>**Outcome:**<br>The study determined the prevalence and classification of DFUs among patients in Beni Suif, Egypt, between 2010 and 2012.<br>• *Population at risk:* 1000<br>• *Total no. of amputations:* 1<br>**Setting:**<br>*Location:* Egypt, Eastern Mediterranean Region<br>*Study setting and level:* Hospital based - Teaching Hospital

[13]	**Title:** Clinical outcome and determinants of amputation in a large cohort of Iranian patients with diabetic foot ulcers<br><br>**Authors:** M. Niakana, M. Dehghankhalilib, A. Pourdavoodc, et al.<br><br>• *Publication Year:* 2020<br>• *Study Design:* Prospective Cohort<br>• *Study Period:* 2014 - 2016<br>• *JBI Quality Score:* High (9)<br><br>**Population:**<br><br>The study population consisted of adult (≥18 years) patients with Type 1 or Type 2 DM and newly diagnosed DFUs, excluding those with peripheral vascular disease, lumbar canal stenosis, neurogenic claudication, previous traumatic amputations, or disabling/psychological disorders.<br><br>• *Mean Age (SD):* 56.8 (13.3)<br>• *Male, %(n):* 70.83% (306)<br><br>**Outcome:**<br><br>The study determined outcomes and predictors of LEA in a large sample of Iranian patients with DFUs<br><br>• *Population at risk:* 432<br>• *Total no. of amputations:* 94<br><br>**Setting:**<br><br>*Location:* Iran, Eastern Mediterranean Region<br><br>*Study setting and level:* Hospital based - Tertiary level
[14]	**Title:** Diabetic foot lesions as seen in Nigerian Teaching Hospital: Pattern and Simple Classification.<br><br>**Authors:** A. Hadadi, H.O. Ghiasi, M. Hajiabdolbaghi, et. al.<br><br>• *Publication Year:* 2014<br>• *Study Design:* Retrospective Cross-Sectional<br>• *Study Period:* 2007- 2010<br>• *JBI Quality Score:* Moderate (6)<br><br>**Population:**<br><br>The study population comprised all patients with diabetic foot admitted to the infectious diseases, surgery, and endocrinology wards of the two hospitals from 2007 to 2010, with diabetes diagnosed according to WHO and ADA criteria.<br><br>• *Mean Age (SD):* 60.84 (10.3)<br>• *Male, %(n):* 57.65% (113)<br><br>**Outcome:**<br><br>The study identified the most common microorganisms involved in DFI to help reduce antibiotic therapy failure and complications.<br><br>• *Population at risk:* 196<br>• *Total no. of amputations:* 89

	**Setting:**<br>*Location:* Iran, Eastern Mediterranean Region<br>*Study setting and level:* Hospital based - Teaching Hospital
[15]	**Title:** Management of diabetic foot ulcer in Babol, North of Iran: an experience on 520 cases<br>**Authors:** N. Janmohammadi, MRH. Roshan, M. Rouhi , et. al.<br>• *Publication Year:* 2012<br>• *Study Design:* Retrospective Cohort<br>• *Study Period:* March 2005 - September 2011<br>• *JBI Quality Score:* Moderate (7)<br>**Population:**<br>The study population comprised patients hospitalized with DFUs at two hospitals affiliated with Babol University of Medical Sciences during the study period.<br>• *Mean Age (SD):* 57.8 (11.2)<br>• *Male, %(n):* 34.03% (177)<br>**Outcome:**<br>The study evaluated DFU management in Babol, Iran, focusing on glycaemic control, wound care, ulcer treatment, and amputation levels.<br>• *Population at risk:* 520<br>• *Total no. of amputations:* 298<br>**Setting:**<br>*Location:* Iran, Eastern Mediterranean Region<br>*Study setting and level:* Hospital based - Teaching Hospital
[16]	**Title:** Correlation Between Amputation of Diabetic Foot and Nephropathy<br>**Authors:** O. Akha, Z. Kashi, A. Makhlough<br>• *Publication Year:* 2010<br>• *Study Design:* Retrospective Cross-Sectional<br>• *Study Period:* 1998 - 2005<br>• *JBI Quality Score:* Moderate (7)<br>**Population:**<br>The study population consisted of patients admitted to Imam Khomeini Hospital in Sari, Iran, presenting with diabetic foot.<br>• *Mean Age (SD):* 57 (12.4)<br>• *Male, %(n):* 52.86% (129)<br>**Outcome:**<br>The study examined the relationship between kidney function and amputation risk in diabetic foot patients by comparing urine protein excretion and serum creatinine between amputees and non-amputees.<br>• *Population at risk:* 244

	• *Total no. of amputations:* 66<br>**Setting:**<br>*Location:* Iran, Eastern Mediterranean Region<br>*Study setting and level:* Hospital based - Teaching Hospital
[17]	**Title:** Frequency of Diabetes Foot Ulcer and Related Factors Among Adult Diabetic Patients in the Diabetes Center of Kerman, Iran<br>**Authors:** Khalooei A, and Meymand MM.<br>• *Publication Year:* 2020<br>• *Study Design:* Prospective Cross-Sectional<br>• *Study Period:* January 2017 - March 2017<br>• *JBI Quality Score:* Moderate (6)<br>**Population:**<br>The study population included adult patients (≥18 years) with Type 2 DM who had received diabetes-related care for at least one year at the Diabetes Center of Kerman University of Medical Sciences.<br>• *Mean Age (SD):* 57.1 (11.9)<br>• *Male, %(n):* 31% (124)<br>**Outcome:**<br>The study assessed the prevalence of DFU and related factors among persons with diabetes at the Diabetes Center of Kerman University of Medical Sciences.<br>• *Population at risk:* 400<br>• *Total no. of amputations:* 10<br>**Setting:**<br>*Location:* Iran, Eastern Mediterranean Region<br>*Study setting and level:* Community - Teaching Hospital
[18]	**Title:** Epidemiological study, risk factors and clinical outcomes of diabetic foot infection in hospitalized patients<br>**Authors:** A. Alikhani, M. Moradi, S. Alikhani<br>• *Publication Year:* 2015<br>• *Study Design:* Retrospective Cohort<br>• *Study Period:* 2013 - 2014<br>• *JBI Quality Score:* Moderate (7)<br>**Population:**<br>The study population included hospitalized patients with DFIs in Sari teaching hospitals (2013–2014); wounds without infection or with only mild infections excluded.<br>• *Mean Age (SD):* 58.3 (10.2)<br>• *Male, %(n):* 54% (108)<br>**Outcome:**

	The study determined outcomes and predictors of LEA in patients with DFU, focusing on infection and related factors.<br>• *Population at risk:* 200<br>• *Total no. of amputations:* 41<br>**Setting:**<br>*Location:* Iran, Eastern Mediterranean Region<br>*Study setting and level:* Hospital based - Teaching Hospital
[19]	**Title:** Septic Diabetic Foot is Not Necessarily an Indication for Amputation<br>**Authors:** A. Shojaiefard, Z. Khorgami, and B. Larijani<br>• *Publication Year:* 2008<br>• *Study Design:* Prospective Cohort<br>• *Study Period:* May 2004 - June 2006<br>• *JBI Quality Score:* Moderate (7)<br>**Population:**<br>The study population included consecutive persons with diabetes from the authors' practices admitted for management of diabetic foot wounds at the authors' discretion.<br>• *Mean Age (SD):* 59 (42-83)<br>• *Male, %(n):* 62.58% (87)<br>**Outcome:**<br>The study described outcomes of a prospective cohort evaluating a treatment protocol for persons with diabetes with and without septic feet, focusing on limb preservation, LEA, and amputation level.<br>• *Population at risk:* 139<br>• *Total no. of amputations:* 4<br>**Setting:**<br>*Location:* Iran, Eastern Mediterranean Region<br>*Study setting and level:* Hospital based - Teaching Hospital
[20]	**Title:** Epidemiological Insights into Diabetic Foot Amputation and its Correlates: A Provincial Study<br>**Authors:** M. Aalaa, A.M. Vahdani, M. Mohajeri Tehrani et. al.<br>• *Publication Year:* 2024<br>• *Study Design:* Retrospective Cross-Sectional<br>• *Study Period:* 2012 - 2020<br>• *JBI Quality Score:* Moderate (8)<br>**Population:**<br>The study population included patients of all ages and both genders with diabetes who underwent LEA due to DFU; excluded records with >20% missing variables.<br>• *Mean Age (SD):* 61.6 (32-85)<br>• *Male, %(n):* 75.86% (525)

	**Outcome:**<br>The study assessed the prevalence of diabetic foot amputation and explored correlates related to the cause and type of amputation among persons with diabetes in Tehran, Iran.<br>• *Population at risk:* 4676<br>• *Total no. of amputations:* 882<br>**Setting:**<br>*Location:* Iran, Eastern Mediterranean Region<br>*Study setting and level:* Hospital based - Multiple
[21]	**Title:** Chronic Complications of Diabetes in Iraq: Experience from Southern Iraq<br>**Authors:** A.A. Mansour<br>• *Publication Year:* 2009<br>• *Study Design:* Retrospective Cross-Sectional<br>• *Study Period:* April 2003 - 28 February 2009<br>• *JBI Quality Score:* Moderate (6)<br>**Population:**<br>The study population included patients with diabetes registered at Al-Faiha Diabetes and Endocrine Centre in Basrah with at least one year of follow-up, excluding those lost to follow-up ≥6 months, with incomplete investigations, diabetes <1 year, pregnancy, Type 1 DM, dilated cardiomyopathy, non-cardiovascular deaths, or aged <20 years.<br>• *Mean Age (SD):* 55 (13.1)<br>• *Male, %(n):* 49% (2414)<br>**Outcome:**<br>The study evaluated the prevalence of chronic complications among Type 2 persons with diabetes in Basrah, Southern Iraq.<br>• *Population at risk:* 4926<br>• *Total no. of amputations:* 33<br>**Setting:**<br>*Location:* Iraq, Eastern Mediterranean Region<br>*Study setting and level:* Community - Not Specified
[22]	**Title:** Foot Abnormalities in Diabetics: Prevalence & Predictors in Basrah, Iraq<br>**Authors:** A. A. Mansour, H. J. Imran<br>• *Publication Year:* 2006<br>• *Study Design:* Prospective Cross-Sectional<br>• *Study Period:* January 2005 - December 2005<br>• *JBI Quality Score:* Moderate (8)<br>**Population:**

	The study population consisted of patients with Type 2 DM attending the outpatient clinics of the General and Teaching Hospitals in Basrah between January and December 2005.<br>• *Mean Age (SD):* 56 (8.4)<br>• *Male, %(n):* 43.95% (80)<br>**Outcome:**<br>The study estimated the prevalence of diabetic foot abnormalities and identified predictors of these abnormalities among Type 2 DM patients in Basrah, Iraq.<br>• *Population at risk:* 182<br>• *Total no. of amputations:* 4<br>**Setting:**<br>*Location:* Iraq, Eastern Mediterranean Region<br>*Study setting and level:* Multiple - Multiple
[23]	**Title:** Prevalence of Foot Ulcers, Foot at Risk and Associated Risk Factors<br>Among Jordanian Diabetics<br>**Authors:** MY. Al Ayed, N. Younes, M. Al-Smady, et. al.<br>• *Publication Year:* 2016<br>• *Study Design:* Prospective Cross-Sectional<br>• *Study Period:* 2016<br>• *JBI Quality Score:* High (9)<br>**Population:**<br>The study population included diabetes patients over 20 years of age visiting the diabetes centre.<br>• *Mean Age (SD):* 58.5 (11.4)<br>• *Male, %(n):* 48.2% (482)<br>**Outcome:**<br>The study examined the prevalence of foot ulcers, “feet at risk,” and associated risk factors among persons with diabetes at the National Center for Diabetes, Endocrinology, and Genetics in Jordan.<br>• *Population at risk:* 1000<br>• *Total no. of amputations:* 17<br>**Setting:**<br>*Location:* Jordan, Eastern Mediterranean Region<br>*Study setting and level:* Community - Not Specified
[24]	**Title:** Prevalence and predictors of diabetic foot syndrome in Type 2 diabetes mellitus in Jordan<br>**Authors:** AS. Jbour, NS. Jarrah, AM. Radaideh, et. al.<br>• *Publication Year:* 2003<br>• *Study Design:* Prospective Cross-Sectional

	• *Study Period:* 1 January 2001 - December 2001<br>• *JBI Quality Score:* Moderate (7)<br>**Population:**<br>The study population included all patients with Type 2 DM who attended the clinic between January and December 2001 and were seen by the principal investigator.<br>• *Mean Age (SD):* 56.1 (10.2)<br>• *Male, %(n):* 52.10% (595)<br>**Outcome:**<br>The study examined foot changes and identified risk factors leading to amputation among patients with Type 2 DM.<br>• *Population at risk:* 1142<br>• *Total no. of amputations:* 53<br>**Setting:**<br>*Location:* Jodan, Eastern Mediterranean Region<br>*Study setting and level:* Not specified
[25]	**Title:** Clinical profile and surgical management of diabetic foot in Benghazi, Libya<br>**Authors:** M. Benkhadoura, M. Alswehly, A. Elbarsha, et. al.<br>• *Publication Year:* 2015<br>• *Study Design:* Prospective Cohort<br>• *Study Period:* 1 June 2008 - 31 May 2013<br>• *JBI Quality Score:* High (9)<br>**Population:**<br>The study population included all consecutive patients admitted to Al-Jala Hospital with diabetic foot.<br>• *Mean Age:* 59<br>• *Male, %(n):* 64.94% (352)<br>**Outcome:**<br>The study outlined patterns and management approaches for DFUs and compared local experience with findings from other published data.<br>• *Population at risk:* 542<br>• *Total no. of amputations:* 185<br>**Setting:**<br>*Location:* Libya, Eastern Mediterranean Region<br>*Study setting and level:* Hospital based - Not Specified
[26]	**Title:** The neurovascular consequences of diabetes: Foot amputation and evaluation of its risk factors and health related economic impact<br>**Authors:** H.I. Naqvi, A. Talib, S.H. Naqvi, et al.

<table>
<tr><td></td><td>

- *Publication Year:* 2020
- *Study Design:* Retrospective Cross-Sectional
- *Study Period:* 1 January 2017 - 31 December 2018
- *JBI Quality Score:* High (10)

Population:

The study population comprised all hospital-admitted patients of any age, excluding those admitted to the ICU or with incomplete data.

- *Mean Age (SD):* 57.0 (13.7)
- *Male, %(n):* 70.49% (1032)

Outcome:

The study determined the frequency and financial burden of diabetic foot amputations and identified risk factors associated with amputation.

- *Population at risk:* 1464
- *Total no. of amputations:* 484

Setting:

Location: Pakistan, Eastern Mediterranean Region

Study setting and level: Hospital based - Tertiary level

</td></tr>
<tr><td>[27]</td><td>

Title: Impact of multidisciplinary foot care team on outcome of diabetic foot ulcer in term of lower extremity amputation at a tertiary care unit in Karachi, Pakistan

Authors: M. Riaz, Z. Miyan, N. Waris, et. al

- *Publication Year:* 2018
- *Study Design:* Retrospective Cross-Sectional
- *Study Period:* January 1997 - December 2006
- *JBI Quality Score:* High (9)

Population:

The study population comprised all subjects with DFUs attending the foot clinic, excluding those who died, were lost to follow-up, or unable to complete treatment.

- *Mean Age (SD):* 53.8 (10.4)
- *Male, %(n):* 70.75% (5656)

Outcome:

The study compared outcomes of DFUs in terms of healing and LEA rates before and after the implementation of a multidisciplinary foot care team.

- *Population at risk:* 7994
- *Total no. of amputations:* 4882

Setting:

Location: Pakistan, Eastern Mediterranean Region

Study setting and level: Hospital based - Tertiary level

</td></tr>
<tr><td>[28]</td><td>

Title: A Study of Risk Factors of Diabetic Foot Ulcers

</td></tr>
</table>

<table>
<tr><td></td><td>

Authors: Anam Shafi, Maaz-Ul-Hassan and Sidra Tufail

- *Publication Year:* 2017
- *Study Design:* Prospective Cross-Sectional
- *Study Period:* 1 January 2017 - 31 July 2017
- *JBI Quality Score:* High (9)

Population:

The study population included persons with diabetes aged 20–75 presenting to surgical OPD/emergency with DFUs; excluded chronic renal failure, or chronic liver disease.

- *Mean Age (SD):* 55.35 (7.12)
- *Male, %(n):* 60% (90)

Outcome:

The study identified risk factors associated with the development of DFUs among patients with diabetes.

- *Population at risk:* 150
- *Total no. of amputations:* 30

Setting:

Location: Pakistan, Eastern Mediterranean Region

Study setting and level: Hospital based - Teaching Hospital

</td></tr>
<tr><td>[29]</td><td>

Title: Role of wound classification in predicting the outcome of Diabetic Foot Ulcer

Authors: A. Gul, A. Basit, S. M. Ali

- *Publication Year:* 2006
- *Study Design:* Prospective Cohort
- *Study Period:* January 1997 - December 2003
- *JBI Quality Score:* Moderate (7)

Population:

The study population consisted of persons with diabetes with foot ulcers who visited BIDE, excluding those with incomplete records.

- *Mean Age (SD):* 53.04 (10.33)
- *Male, %(n):* 65% (130)

Outcome:

The study compared the effectiveness of the Wagner and University of Texas classification systems in guiding therapy and predicting outcomes in DFUs.

- *Population at risk:* 200
- *Total no. of amputations:* 48

Setting:

Location: Pakistan, Eastern Mediterranean Region

Study setting and level: Hospital based - Not Specified

</td></tr>
</table>

[30]	**Title:** Frequency of Lower Extremity Amputation in Diabetics with Reference to Glycemic Control and Wagner's Grades<br>**Authors:** S. Imran, R. Ali, G. Mahboob<br>• *Publication Year:* 2004<br>• *Study Design:* Prospective Cross-Sectional<br>• *Study Period:* August 1999 - August 2002<br>• *JBI Quality Score:* Moderate (6)<br>**Population:**<br>The study population included patients with diabetic foot admitted to the orthopaedic ward, excluding those with previous major amputations due to diabetic foot or amputations from non-diabetic causes.<br>• *Mean Age (SD):* 50.88 (11.06)<br>• *Male, %(n):* 61.66% (37)<br>**Outcome:**<br>The study determined the frequency of minor and major amputations in persons with diabetes across different Wagner grades and correlated the findings with glycaemic control.<br>• *Population at risk:* 60<br>• *Total no. of amputations:* 42<br>**Setting:**<br>*Location:* Pakistan, Eastern Mediterranean Region<br>*Study setting and level:* Hospital based - Tertiary level
[31]	**Title:** A Comparative Study on Diabetic Foot Ulcers Leading to Amputations<br>**Authors:** R. Rehman, F. R. Malik, Z. Rehman<br>• *Publication Year:* 2016<br>• *Study Design:* Prospective Cross-Sectional<br>• *Study Period:* November 2015 - February 2016<br>• *JBI Quality Score:* Moderate (6)<br>**Population:**<br>The study population included diagnosed and admitted patients with Type 1 or Type 2 DM who were non-smokers, had no visible foot deformities (other than diabetes-related ulcers or amputations), and were newly diagnosed, excluding critically ill, gestational, or non-consenting patients.<br>• *Mean Age (SD):* 53.55 (11.58)<br>• *Male, %(n):* 35.71% (40)<br>**Outcome:**<br>The study determined and compared the frequency of foot ulcers leading to amputations by gender, diabetes type, and disease duration.<br>• *Population at risk:* 112<br>• *Total no. of amputations:* 40

	**Setting:**<br>*Location:* Pakistan, Eastern Mediterranean Region<br>*Study setting and level:* Hospital based - Teaching Hospital
[32]	**Title:** Characteristics of a large cohort of patients with diabetes having at-risk feet and outcomes in patients with foot ulceration referred to a tertiary care diabetes unit<br>**Authors:** M. Riaz, Z. Miyan, S. I. Zaidi, et. al.<br>• *Publication Year:* 2014<br>• *Study Design:* Prospective Cross-Sectional<br>• *Study Period:* January 2004 - April 2012<br>• *JBI Quality Score:* Moderate (7)<br>**Population:**<br>The study population included patients with diabetes attending the Baqai Institute of Diabetology and Endocrinology, including those with and without foot problems.<br>• *Mean Age (SD):* 48.73 (12.57)<br>• *Male, %(n):* 71.40% (12937)<br>**Outcome:**<br>The study identified clinical and biochemical characteristics of persons with diabetes at risk for foot ulceration and evaluated outcomes among those with ulcers in a large population cohort.<br>• *Population at risk:* 18119<br>• *Total no. of amputations:* 293<br>**Setting:**<br>*Location:* Pakistan, Eastern Mediterranean Region<br>*Study setting and level:* Hospital based - Tertiary level
[33]	**Title:** Risk factors associated with lower extremity amputation in Sudanese individuals with diabetes: The need for improvement in primary health care system<br>**Authors:** A. T. E. Elkhider, A. O. Almobark, S. Badi, et. al.<br>• *Publication Year:* 2020<br>• *Study Design:* Retrospective Cohort<br>• *Study Period:* 1 July 2016 - 30 July 2018<br>• *JBI Quality Score:* High (9)<br>**Population:**<br>The study population comprised adult individuals (≥18 years) with diabetes and foot ulcers, with or without amputation, who consented to participate, excluding groups with no diabetic amputations, those diagnosed with diabetes for ≤6 months, individuals with incomplete records, and those who did not provide consent.<br>• *Mean Age: Not reported*<br>• *Male, %(n):* 71.11% (224)<br>**Outcome:**

	The study assessed the prevalence and risk factors associated with LEA in DFU patients.<br>• *Population at risk:* 315<br>• *Total no. of amputations:* 54<br>**Setting:**<br>*Location:* Sudan, Eastern Mediterranean Region<br>*Study setting and level:* Hospital based - Not Specified
[34]	**Title:** Diabetic Heel Ulcer in the Sudan: Determinants of Outcome<br>**Authors:** H.E. Bakheit, M.F. Mohamed, S.E.I. Mahadi<br>• *Publication Year:* 2012<br>• *Study Design:* Prospective Cross-Sectional<br>• *Study Period:* May 2003 - January 2005<br>• *JBI Quality Score:* Moderate (7)<br>**Population:**<br>The study population included persons with diabetes with heel ulcers attending the foot clinic.<br>• *Mean Age (SD):* 52.02 (13.6)<br>• *Male, %(n):* 63% (63)<br>**Outcome:**<br>The study analysed determinants of healing in diabetic heel ulcers and assessed long-term outcomes, including healing rates and amputation outcomes.<br>• *Population at risk:* 100<br>• *Total no. of amputations:* 22<br>**Setting:**<br>*Location:* Sudan, Eastern Mediterranean Region<br>*Study setting and level:* Hospital based - Tertiary level
[35]	**Title:** Diabetic foot and disaster; risk factors for amputation during the Syrian crisis<br>**Authors:** A. Abdul Aziz, M. Alsabek<br>• *Publication Year:* 2019<br>• *Study Design:* Retrospective Cohort<br>• *Study Period:* January 2012 - December 2017<br>• *JBI Quality Score:* High (9)<br>**Population:**<br>The study population included diabetic foot outpatients attending the Damascus Teaching Hospital diabetic clinic; excluded leg-only lesions, Wagner grade 0 neuropathic symptoms, cases not followed to healing (noncompliance or death).<br>• *Mean Age:* 60<br>• *Male, %(n):* 56.72% (1138)

	**Outcome:**<br>The study established risk factors for diabetic foot amputation—both minor and major—by analysing age, gender, lesion type and location, infection status, vascular status, and amputation rate.<br>• *Population at risk:* 2006<br>• *Total no. of amputations:* 634<br>**Setting:**<br>*Location:* Syria, Eastern Mediterranean Region<br>*Study setting and level:* Hospital based - Teaching Hospital
[36]	**Title:** Analysis of Disability due to Diabetes Mellitus in a Large City<br>**Authors:** A. Tanirbergenova, M. Kamaliev, Z. Akanov, et. al<br>• *Publication Year:* 2021<br>• *Study Design:* Retrospective Cross-Sectional<br>• *Study Period:* 2013 - 2017<br>• *JBI Quality Score:* High (9)<br>**Population:**<br>The study population included residents of Almaty with diabetes identified as disabled (child or I/II/III groups) at initial 2013–2017 examinations by territorial medical-social expertise.<br>• *Mean Age (SD):* 58.8 (+-1.3)<br>• *Male, %(n):* 52.53% (342)<br>**Outcome:**<br>The study examined the dynamics and causes of disability in the population of Almaty, Kazakhstan, due to diabetes.<br>• *Population at risk:* 651<br>• *Total no. of amputations:* 82<br>**Setting:**<br>*Location:* Kazakhstan, European Region<br>*Study setting and level:* Country – Multiple Levels
[37]	**Title:** Risk factors for amputation in patients with diabetic foot infection: a prospective study<br>**Authors:** Serhat Uysal, Bilgin Arda, Meltem I. Taşbakan<br>• *Publication Year:* 2017<br>• *Study Design:* Prospective Cohort<br>• *Study Period:* June 2012 – June 2014<br>• *JBI Quality Score:* High (9)<br>**Population:**

	The study population included patients with DFIs admitted to the Diabetic Foot Council at Ege University (June 2012–June 2014); excluded non-infected ulcers, rapidly progressive/terminal illness, decliners, missing data, or lost to follow-up.<br>• *Mean Age (Range):* 62.4 (*SD*)<br>• *Male, %(n):* 67.54% (256)<br>**Outcome:**<br>The study evaluated independent risk factors for osteomyelitis, amputation, and major amputation in patients with DFI using standardized scoring methods.<br>• *Population at risk:* 379<br>• *Total no. of amputations:* 126<br>**Setting:**<br>*Location:* Turkey, South-East Asia Region<br>*Study setting and level:* Hospital based - Tertiary level
[38]	**Title:** Prevalence of diabetic foot in patients with diabetes mellitus undergoing dialysis treatment in a tertiary-level hospital in Argentina<br>**Authors:** M.B. Spinelli, V.M. Cafruni, N. Lucero Viviani, et al.<br>• *Publication Year:* 2025<br>• *Study Design:* Prospective Cross-Sectional<br>• *Study Period:* 1 December 2022 - 31 September 2024<br>• *JBI Quality Score:* Moderate (6)<br>**Population:**<br>The study population included patients >18 with confirmed Type 1 or 2 diabetes at a tertiary hospital, with complete EMR and consent for foot exam; excluded incomplete records, incorrect/other-centre RRT, or no/declined foot exam.<br>• *Mean Age (Range):* 65.9 (34-88)<br>• *Male, %(n):* 70.37% (38)<br>**Outcome:**<br>The study assessed the prevalence of diabetic foot and other associated conditions in patients with DM undergoing renal replacement therapy, including haemodialysis and peritoneal dialysis, at a tertiary hospital in Argentina.<br>• *Population at risk:* 54<br>• *Total no. of amputations:* 17<br>**Setting:**<br>*Location:* Argentina, Region of the Americas<br>*Study setting and level:* Hospital based - Tertiary level
[39]	**Title:** Analysis of Diabetes Mellitus-Related Amputations in the State of Espírito Santo, Brazil<br>**Authors:** W.J.T. Costa, N. Penha-Silva, I.M.P. Bezerra, et. al.<br>• *Publication Year:* 2020<br>• *Study Design:* Retrospective Cross-Sectional

	• *Study Period:* 2003 - 2012<br>• *JBI Quality Score:* High (9)<br>**Population:**<br>The study population comprised individuals with Type 1 or Type 2 DM, with or without concomitant hypertension, who were registered and followed by the SisHiperdia system in Espírito Santo and underwent amputation due to diabetes between 2003 and 2012.<br>• *Mean Age: Not reported*<br>• *Male, %(n):* 33.36% (21416)<br>**Outcome:**<br>The study analysed the prevalence of diabetes-related amputations, comorbidities, and associated risk factors among persons with diabetes in Espírito Santo, Brazil.<br>• *Population at risk:* 64196<br>• *Total no. of amputations:* 1357<br>**Setting:**<br>*Location:* Brazil, Region of the Americas<br>*Study setting and level:* Country – Multiple Levels
[40]	**Title:** Relationship between amputation and risk factors in individuals with diabetes mellitus: A study with Brazilian patients<br>**Authors:** AM. Mantovania, CEPT. Fregonesib, MR. Palma, et al.<br>• *Publication Year:* 2017<br>• *Study Design:* Prospective Cross-Sectional<br>• *Study Period:* 2007 – 2014<br>• *JBI Quality Score:* High (9)<br>**Population:**<br>The study population comprised participants (n = 165) recruited from the Diabetic Foot Program at a Brazilian university between 2007 and 2014, with data extracted from clinical records.<br>• *Mean Age (SD):* 65.73 (6.83)<br>• *Male, %(n):* 40% (66)<br>**Outcome:**<br>The study evaluated the relationship between amputation and associated risk factors in people with DM.<br>• *Population at risk:* 165<br>• *Total no. of amputations:* 51<br>**Setting:**<br>*Location:* Brazil, Region of the Americas<br>*Study setting and level:* Hospital based - Teaching Hospital

[41]	**Title:** Risk factors for ulceration and amputation in diabetic foot: study in a cohort of 496 patients<br>**Authors:** A.M. Neto, D.E. Zantut-Wittmann, T.D. Fernandes, et. al.<br>• *Publication Year:* 2012<br>• *Study Design:* Retrospective Cohort<br>• *Study Period:* January 2007- January 2010<br>• *JBI Quality Score:* High (9)<br>**Population:**<br>The study population comprised patients with diabetes and an established foot at risk or diabetic foot, followed up at the Endocrinology Division of the University of Campinas from January 2007 to January 2010.<br>• *Mean Age (SD):* 60.2 (11.8)<br>• *Male, %(n):* 69.6% (348)<br>**Outcome:**<br>The study determined the epidemiological presentation of diabetic foot and assessed whether simple, easily measurable variables could predict its development.<br>• *Population at risk:* 496<br>• *Total no. of amputations:* 52<br>**Setting:**<br>*Location:* Brazil, Region of the Americas<br>*Study setting and level:* Hospital based - Teaching Hospital
[42]	**Title:** Baseline characteristics and risk factors for ulcer, amputation and severe neuropathy in diabetic foot at risk: the BRAZUPA study<br>**Authors:** MCR. Parisi, AM. Neto, FH. Menezes, et al.<br>• *Publication Year:* 2016<br>• *Study Design:* Retrospective Cross-Sectional<br>• *Study Period:* June 2012 - July 2014<br>• *JBI Quality Score:* High (9)<br>**Population:**<br>The study population included patients referred for evaluation of foot-at-risk to 19 specialized diabetic centres across Brazil.<br>• *Mean Age (SD):* 57.67 (14.23)<br>• *Male, %(n):* 41.37% (602)<br>**Outcome:**<br>The study collected clinical and epidemiological data on a large Brazilian cohort of persons with diabetes to identify factors associated with ulcer and amputation risk.<br>• *Population at risk:* 1455<br>• *Total no. of amputations:* 199<br>**Setting:**

<table>
<tr><td></td><td>Location: Brazil, Region of the Americas
Study setting and level: Hospital based - Specialist</td></tr>
<tr><td>[43]</td><td>Title: Risk Factors for Foot Amputation in Patients Hospitalized for Diabetic Foot Infection
Authors: M. T. V. Quilici, F. de Sá Del Fiol, A. E. F. Vieira, et. al.
• Publication Year: 2016
• Study Design: Prospective Cross-Sectional
• Study Period: Not reported
• JBI Quality Score: High (9)
Population:
The study population included adult (≥18 years) persons with diabetes with infected lower limb ulcers hospitalized at the Vascular Surgery Clinic of the Conjunto Hospitalar de Sorocaba who consented to participate.
• Mean Age (Range): 62 (31.9-89.7)
• Male, %(n): 68% (68)
Outcome:
The study identified and quantified clinical, biochemical, epidemiological, and behavioral predictors of amputation outcomes among persons with diabetes hospitalized for foot infections.
• Population at risk: 100
• Total no. of amputations: 39
Setting:
Location: Brazil, Region of the Americas
Study setting and level: Hospital based - Tertiary level</td></tr>
<tr><td>[44]</td><td>Title: Prevalence of Diabetic Foot and Associated Factors in Family Health Units in the City of Recife, Pernambuco, Brazil
Authors: I. C. R. Vieira-Santos, W. V. de Souza, E. F. de Carvalho, et. al
• Publication Year: 2005
• Study Design: Retrospective Cross-Sectional
• Study Period: 2005
• JBI Quality Score: High (9)
Population:
The study population included persons with diabetes registered with Family Health Units in Recife.
• Mean Age (SD): 61 (12.1)
• Male, %(n): 29.83% (410)
Outcome:</td></tr>
</table>

	The study identified the proportion of individuals with diabetic foot treated at family health units in Recife, Pernambuco, Brazil, and examined associations with unhealthy habits and socioeconomic factors.<br>• *Population at risk:* 1374<br>• *Total no. of amputations:* 32<br>**Setting:**<br>*Location:* Brazil, Region of the Americas<br>*Study setting and level:* Community - Not Specified
[45]	**Title:** Characteristics of diabetic foot injuries and their complications<br>**Authors:** Carla Luiza da Silva, Cintia Moleta, Emanoel Severo, et al.<br>• *Publication Year:* 2012<br>• *Study Design:* Retrospective Cross-Sectional<br>• *Study Period:* May 2010 - October 2010<br>• *JBI Quality Score:* Moderate (7)<br>**Population:**<br>The study population included people registered at the unit with diabetes, diabetic foot injuries, or amputations.<br>• *Mean Age:* Not reported<br>• *Male, %(n):* Not reported<br>**Outcome:**<br>The study verified the prevalence of LLAs due to infected diabetic ulcers in patients at a Family Health Unit and analysed related physiological changes.<br>• *Population at risk:* 40<br>• *Total no. of amputations:* 3<br>**Setting:**<br>*Location:* Brazil, Region of the Americas<br>*Study setting and level:* Community - Not Specified
[46]	**Title:** Association between hospitalization due to diabetes mellitus and diabetic foot amputation<br>**Authors:** A. D. B. Marques, L. M. S. da Silva, T. M. M. Moreira, et. al<br>• *Publication Year:* 2018<br>• *Study Design:* Retrospective Cohort<br>• *Study Period:* 2010 - 2015<br>• *JBI Quality Score:* Moderate (6)<br>**Population:**<br>The study population included hospitalized persons with diabetes.<br>• *Mean Age:* Not reported<br>• *Male, %(n):* Not reported

	**Outcome:**<br>The study analysed the association between diabetes-related hospitalizations and limb amputations to inform primary health care planning and prevention strategies.<br>• *Population at risk:* 22872<br>• *Total no. of amputations:* 5914<br>**Setting:**<br>*Location:* Brazil, Region of the Americas<br>*Study setting and level:* Hospital based - Not Specified
[47]	**Title:** Factors associated with diabetic foot amputations<br>**Authors:** Isabel Cristina Ramos Vieira Santos, Eduardo Freese de Carvalho, Wayner Vieira de Souza, et. al.<br>• *Publication Year:* 2014<br>• *Study Design:* Prospective Cross-Sectional<br>• *Study Period:* Not reported<br>• *JBI Quality Score:* Moderate (7)<br>**Population:**<br>The study population included diabetic foot patients admitted for treatment (n=151) with ischemic, neuropathic, or infectious diabetic foot and Wagner grade 2–5 ulcers; excluded if never attended primary care or unable to answer due to physical/mental conditions.<br>• *Mean Age: Not reported*<br>• *Male, %(n):* 56.93% (78)<br>**Outcome:**<br>The study identified associations between amputations and factors related to individuals, comorbidities, and primary care received.<br>• *Population at risk:* 137<br>• *Total no. of amputations:* 85<br>**Setting:**<br>*Location:* Brazil, Region of the Americas<br>*Study setting and level:* Hospital based - Tertiary level
[48]	**Title:** The prevalence and factors associated with diabetic foot amputations<br>**Authors:** Santos I. C. R. V., Sobreira C. M. M.; Nunes É N. S., et. al.<br>• *Publication Year:* 2013<br>• *Study Design:* Prospective Cross-Sectional<br>• *Study Period:* 2008 - 2010<br>• *JBI Quality Score:* Moderate (7)<br>**Population:**<br>The study population included all individuals with diabetic foot hospitalized in vascular clinics at four Pernambuco hospitals (July 1, 2008–July 1, 2010).

	• *Mean Age: Not reported*<br>• *Male, %(n):* 54.67% (117)<br>**Outcome:**<br>The study determined the prevalence of diabetic foot amputations and analysed associations with person-level and primary care factors.<br>• *Population at risk:* 214<br>• *Total no. of amputations:* 107<br>**Setting:**<br>*Location:* Brazil, Region of the Americas<br>*Study setting and level:* Hospital based - Not Specified
[49]	**Title:** Factors associated with non-traumatic amputation in people with Diabetes Mellitus: a cross-sectional study<br>**Authors:** I.V.Diniz, P. Simplício de Oliveira, I.C. Ramos Vieira Santos, et. al.<br>• *Publication Year:* 2019<br>• *Study Design:* Prospective Cross-Sectional<br>• *Study Period:* June 2013 - June 2014<br>• *JBI Quality Score:* Moderate (7)<br>**Population:**<br>The study population included people with diabetes aged >18 with foot complications, excluded foot ulcers of other aetiologies.<br>• *Mean Age: Not reported*<br>• *Male, %(n):* 45.28% (96)<br>**Outcome:**<br>The study verified factors associated with non-traumatic amputations among persons with DM, examining links with sociodemographic characteristics.<br>• *Population at risk:* 212<br>• *Total no. of amputations:* 44<br>**Setting:**<br>*Location:* Brazil, Region of the Americas<br>*Study setting and level:* Hospital based - Not Specified
[50]	**Title:** Demographic and Clinical Characteristics of Dominican Adults Admitted to a Diabetic Foot Clinic in the Dominican Republic, 2015<br>**Authors:** G. A. Bonilla, P. P. Hornsby, A. F. Pannone<br>• *Publication Year:* 2015<br>• *Study Design:* Retrospective Cross-Sectional<br>• *Study Period:* 1 January 2015 - 31 December 2015<br>• *JBI Quality Score:* High (9)<br>**Population:**

	The study population included Dominican adults >18 admitted to INDEN's diabetic foot clinic between Jan 1 and Dec 31, 2015; excluded non-DR-born and ambulatory patients lacking chart access numbers.<br>• *Mean Age (SD):* 64.6 (12.6)<br>• *Male, %(n):* 46.97% (210)<br>**Outcome:**<br>The study identified demographic and clinical characteristics of Dominican adults admitted to the largest diabetic foot clinic in the Dominican Republic and compared characteristics by sex to better understand diabetes severity.<br>• *Population at risk:* 447<br>• *Total no. of amputations:* 214<br>**Setting:**<br>*Location:* Dominican Republic, Region of the Americas<br>*Study setting and level:* Hospital based - Tertiary level
[51]	**Title:** Rate of lower extremity amputations in patients with diabetes<br>**Authors:** N. Cisneros-González, IJ. Ascencio-Montiel, VN. Libreros-Bango et. al.<br>• *Publication Year:* 2015<br>• *Study Design:* Retrospective Cross-Sectional<br>• *Study Period:* 2004 and 2013<br>• *JBI Quality Score:* High (9)<br>**Population:**<br>The study population included patients diagnosed with DM, identified through the Comprehensive Health Care Information System and hospital records of the Medical Statistics System (Data-Mart) of the IMSS, excluding traumatic amputations.<br>• *Mean Age:* (61.7 and 65.6 years old for minor and major amputations respectively<br>• *Male, %(n):* Not reported<br>**Outcome:**<br>The study described LEA rates in subjects with DM in the Mexican Social Security Institute and compared rates between 2004 and 2013.<br>• *Population at risk:* 5750983<br>• *Total no. of amputations:* 15621<br>**Setting:**<br>*Location:* Mexico, Region of the Americas<br>*Study setting and level:* Hospital based - Not Specified
[52]	**Title:** Evaluation of management, control, complications and psychosocial aspects of diabetics in Bangladesh: DiabCare Bangladesh 2008<br>**Authors:** ZA. Latif, A. Jain, MM Rahman<br>• *Publication Year:* 2011

	• *Study Design:* Prospective Cross-Sectional<br>• *Study Period:* 1 March 2009 - 31 March 2009<br>• *JBI Quality Score:* High (9)<br>**Population:**<br>The study population comprised patients with Type 1 or Type 2 DM.<br>• *Mean Age (SD):* 50.9 (11.4)<br>• *Male, %(n):* 51.39% (956)<br>**Outcome:**<br>The study evaluated diabetes management, control, complications, psychosocial aspects, and physicians' perceptions and practices regarding diabetes management.<br>• *Population at risk:* 1860<br>• *Total no. of amputations:* 842<br>**Setting:**<br>*Location:* Bangladesh, South-East Asia Region<br>*Study setting and level:* Multiple - Multiple
[53]	**Title:** Long term outcomes after incident diabetic foot ulcer: Multicenter large cohort prospective study (EDI-FOCUS investigators) epidemiology of diabetic foot complications study Epidemiology of diabetic foot complications study<br>**Authors:** Ashu Rastogia, Ghanshyam Goyalb, Rajesh Kesavanc, et. al<br>• *Publication Year:* 2020<br>• *Study Design:* Prospective Cohort<br>• *Study Period:* January 2005 - December 2018<br>• *JBI Quality Score:* High (9)<br>**Population:**<br>The study population comprised consecutive individuals with Type 2 DM, with or without new or current neuropathic foot ulcers, presenting to five referral centres in India between January 2005 and December 2018, excluding those with prior ulcers, amputations, active Charcot neuroarthropathy, or significant peripheral arterial disease (ABI < 0.7).<br>• *Mean Age (Range):* 58 (50-65)<br>• *Male, %(n):* 74.09% (2134)<br>**Outcome:**<br>The long-term prospective study evaluated limb amputation and mortality outcomes following a first neuropathic DFU.<br>• *Population at risk:* 2880<br>• *Total no. of amputations:* 844<br>**Setting:**<br>*Location:* India, South-East Asia Region<br>*Study setting and level:* Hospital based - Tertiary level

<table>
<tr><td>[54]</td><td>

Title: Diabetic Foot: Clinical Aspect and Therapeutic Outcome

Authors: A. H. Zargar, M. H. Bhat, A. I. Wani, et. al.

- *Publication Year:* 2005
- *Study Design:* Retrospective Cohort
- *Study Period:* 1996 - 2000
- *JBI Quality Score:* High (9)

Population:

The study population included persons with diabetes admitted to the endocrinology department at Sher-i-Kashmir Institute of Medical Sciences, Srinagar.

- *Mean Age (SD):* 52.37 (9.45)
- *Male, %(n):* Not reported

Outcome:

The study explored the clinical aspects, progression, and therapeutic outcomes of diabetic foot conditions; specific outcomes were not reported.

- *Population at risk:* 49
- *Total no. of amputations:* 7

Setting:

Location: India, South-East Asia Region

Study setting and level: Hospital based - Tertiary level

</td></tr>
<tr><td>[55]</td><td>

Title: Evaluation of foot problems among diabetics in rural population

Authors: B. Mehra, A. Thawait, S. Karandikar, et al.

- *Publication Year:* 2006
- *Study Design:* Prospective Cross-Sectional
- *Study Period:* January 2002 - May 2003
- *JBI Quality Score:* Moderate (6)

Population:

The study population consisted of persons with diabetes with diabetic foot lesions.

- *Mean Age (SD):* 55.6 (+-12)
- *Male, %(n):* 69.6% (348)

Outcome:

The study obtained an initial data sample to identify common pedal complications of DM and to inform the development of a podiatric foot health education program.

- *Population at risk:* 500
- *Total no. of amputations:* 18

Setting:

Location: India, South-East Asia Region

Study setting and level: Hospital based - Teaching Hospital

</td></tr>
</table>

[56]	**Title:** Diabetic Foot Ulcer Complications and Its Management— a Medical College-Based Descriptive Study in Odisha, an Eastern State of India<br><br>**Authors:** Muduli IC, Ansar P. P. & Panda C, et al.<br><br>• *Publication Year:* 2015<br>• *Study Design:* Prospective Cohort<br>• *Study Period:* January 2009 - October 2010<br>• *JBI Quality Score:* Moderate (7)<br><br>**Population:**<br><br>The study population consisted of 60 consecutive cases of DFU treated at MKCG Medical College, Berhampur, from January 2009 to October 2010, excluding those with other ulcer-causing diseases or who could not be followed up for at least six weeks.<br><br>• *Mean Age (SD):* 54.57 (13)<br>• *Male, %(n):* 70% (42)<br><br>**Outcome:**<br><br>The study profiled DFU cases by Meggitt–Wagner grade, complications, management strategies, below-knee amputation rates, and mortality to assess outcomes of surgical interventions.<br><br>• *Population at risk:* 60<br>• *Total no. of amputations:* 17<br><br>**Setting:**<br><br>*Location:* India, South-East Asia Region<br><br>*Study setting and level:* Hospital based - Tertiary level
[57]	**Title:** Transcutaneous oxygen pressure (TcPO2) and ulcer outcome in diabetic patients: Is there any correlation?<br><br>**Authors:** Mohammad Zubair, Jamal Ahmad<br><br>• *Publication Year:* 2018<br>• *Study Design:* Prospective Cohort<br>• *Study Period:* December 2010 - December 2016<br>• *JBI Quality Score:* Moderate (7)<br><br>**Population:**<br><br>The study population included patients with Type 2 DM who had foot ulcers during the study; excluded inflammatory/infectious/autoimmune/rheumatic/cancer/hematologic diseases, severe renal/liver failure, anti-inflammatory treatment, and recent VTE.<br><br>• *Mean Age (SD):* 48.03 (12.74)<br>• *Male, %(n):* 18.75% (36)<br><br>**Outcome:**<br><br>The study examined ulcer healing and amputation rates and assessed correlations between $TcPO_2$ levels and ulcer outcomes in persons with diabetes.

	• *Population at risk:* 192<br>• *Total no. of amputations:* 148<br>**Setting:**<br>*Location:* India, South-East Asia Region<br>*Study setting and level:* Hospital based - Tertiary level
[58]	**Title:** Incidence, risk factors for amputation among patients with diabetic foot ulcer in a North Indian tertiary care hospital<br>**Authors:** M. Zubaira, A. Malika, J. Ahmad<br>• *Publication Year:* 2011<br>• *Study Design:* Prospective Cohort<br>• *Study Period:* December 2008 - March 2011<br>• *JBI Quality Score:* Moderate (7)<br>**Population:**<br>The study population included persons with diabetes admitted to the Centre for Diabetes and Endocrinology, AMU Aligarh, with foot ulcers developing Dec 2008–Mar 2011; Wagner grade 0 excluded.<br>• *Mean Age (SD):* 51.1 (11.4)<br>• *Male, %(n):* 64.81% (105)<br>**Outcome:**<br>The study evaluated the incidence and risk factors for amputations among DFU patients at a tertiary hospital.<br>• *Population at risk:* 162<br>• *Total no. of amputations:* 46<br>**Setting:**<br>*Location:* India, South-East Asia Region<br>*Study setting and level:* Hospital based - Specialist
[59]	**Title:** Prevalence and predictors of infected diabetic foot ulcers (DFU)<br>and DFU-related osteomyelitis amongst industrial workers<br>wearing occupational safety footwear<br>**Authors:** Sunetra Mondal, Moushumi Lodh, Sambit Sahoo, et al.<br>• *Publication Year:* 2025<br>• *Study Design:* Retrospective Cross-Sectional<br>• *Study Period:* 1 April 2018 - 31 March 2023<br>• *JBI Quality Score:* Moderate (6)<br>**Population:**<br>The study population included patients ≥18 with Type 2 DM and DFU with clinical infection hospitalized Apr 1, 2018–Mar 31, 2023; excluded significant non-workplace trauma, prolonged barefoot walking, musculoskeletal disease, major

	arteriopathy/vasculitis, <1-year employment in mines/steel plants, or antibiotics in past 4 weeks.<br>• *Mean Age (SD):* 53.3 (8.2)<br>• *Male, %(n):* 94.39% (101)<br>**Outcome:**<br>The study described the clinical and microbiologic profile of DFU in individuals wearing occupational footwear and identified risk factors for DFU-related osteomyelitis.<br>• *Population at risk:* 331<br>• *Total no. of amputations:* 58<br>**Setting:**<br>*Location:* India, South-East Asia Region<br>*Study setting and level:* Hospital based - Tertiary level
[60]	**Title:** Early screening for foot problems in people with diabetes is the need of the hour: 'Save the Feet and Keep Walking Campaign' in India<br>**Authors:** V. Viswanathan, A. Gupta, A. Devarajan, et al.<br>• *Publication Year:* 2024<br>• *Study Design:* Prospective Cross-Sectional<br>• *Study Period:* 10 July 2022 - 10 August 2022<br>• *JBI Quality Score:* Moderate (7)<br>**Population:**<br>The study population included people with diabetes; incomplete information excluded.<br>• *Mean Age: Not reported*<br>• *Male, %(n):* 68.69% (22848)<br>**Outcome:**<br>The study's aim was not specified, though it reported on diabetic foot prevalence.<br>• *Population at risk:* 33259<br>• *Total no. of amputations:* 5088<br>**Setting:**<br>*Location:* India, South-East Asia Region<br>*Study setting and level:* Community - Primary level
[61]	**Title:** Prevalence of Chronic Complications, Their Risk Factors, and the Cardiovascular Risk Factors among Patients with Type 2 DM Attending the Diabetic Clinic at a Tertiary Care Hospital in Sri Lanka<br>**Authors:** Maulee Hiromi Arambewela, Noel P. Somasundaram, Hettiarachchige Buddhi Pradeep Ranjan Jayasekara, et. al.<br>• *Publication Year:* 2018<br>• *Study Design:* Prospective Cross-Sectional

	• *Study Period:* January 2016 - July 2016<br>• *JBI Quality Score:* Moderate (7)<br>**Population:**<br>The study population included patients with Type 2 DM attending the National Hospital of Sri Lanka diabetic clinic (Jan–Jul 2016); excluded pregnant patients, gestational diabetes, and Type 1 DM.<br>• *Mean Age (SD):* 58.3 (10.3)<br>• *Male, %(n):* 27.33% (820)<br>**Outcome:**<br>The study determined the prevalence of macrovascular and microvascular complications and their associated risk factors, including coronary artery disease, among patients with Type 2 DM.<br>• *Population at risk:* 3000<br>• *Total no. of amputations:* 54<br>**Setting:**<br>*Location:* Sri Lanka, South-East Asia Region<br>*Study setting and level:* Hospital based - Tertiary level
[62]	**Title:** Thailand Diabetes Registry Project: Prevalence and Risk Factors Associated with Lower Extremity Amputation in Thai Diabetics<br>**Authors:** S. Krittiyawong, C. Ngarmukos, Y. Benjasuratwong<br>• *Publication Year:* 2006<br>• *Study Design:* Prospective Cross-Sectional<br>• *Study Period:* April 2003 - December 2003<br>• *JBI Quality Score:* High (9)<br>**Population:**<br>The study population included persons with diabetes attending outpatient clinics who were available for follow-up for at least one year.<br>• *Mean Age (SD):* 59.4 (13.5)<br>• *Male, %(n):* 35.16% (3312)<br>**Outcome:**<br>The study determined the prevalence and risk factors associated with LEA among Thai persons with diabetes, including history of ulcer, peripheral vascular disease, and retinopathy.<br>• *Population at risk:* 9419<br>• *Total no. of amputations:* 142<br>**Setting:**<br>*Location:* Thailand, South-East Asia Region<br>*Study setting and level:* Hospital based - Multiple

[63]	**Title:** Prevalence of Diabetic Foot Ulcers and Risk Classifications in Type 2 DM Patients at Rajavithi Hospital<br><br>**Authors:** V. Sarinnapakorn, T. Sunthorntepwarakul, C. Deerochanawong, et. al.<br><br>• *Publication Year:* 2016<br>• *Study Design:* Prospective Cross-Sectional<br>• *Study Period:* 2014<br>• *JBI Quality Score:* High (9)<br><br>**Population:**<br><br>The study population included patients with Type 2 DM at Rajavithi Hospital, age >18, with informed consent, excluded Type 1, gestational, or secondary DM.<br><br>• *Mean Age (SD):* 63.8 (13.7)<br>• *Male, %(n):* 34.73% (206)<br><br>**Outcome:**<br><br>The study assessed the prevalence of DFU in Type 2 DM patients at Rajavithi Hospital, classifying patients by amputation risk per Thai guidelines and evaluating correlations with ABI and CAVI.<br><br>• *Population at risk:* 593<br>• *Total no. of amputations:* 13<br><br>**Setting:**<br><br>*Location:* Thailand, South-East Asia Region<br><br>*Study setting and level:* Hospital based - Tertiary level
[64]	**Title:** Vascular Complications in Non-Insulin Dependent Diabetics in Thailand<br><br>**Authors:** Thai Multicenter Research Group on DM<br><br>• *Publication Year:* 1994<br>• *Study Design:* Prospective Cross-Sectional<br>• *Study Period:* January 1993 - December 1993<br>• *JBI Quality Score:* Moderate (6)<br><br>**Population:**<br><br>The study population included patients with non–insulin-dependent DM attending diabetic or general medical clinics, excluding those younger than 15 years or with diabetic ketoacidosis, malnutrition-related diabetes, insulin-dependent diabetes, or gestational diabetes.<br><br>• *Mean Age (SD):* 42 (8.2)<br>• *Male, %(n):* 30.43% (627)<br><br>**Outcome:**<br><br>The study determined the prevalence of microvascular and macrovascular complications in patients with non-insulin-dependent DM (NIDDM) in Thailand.<br><br>• *Population at risk:* 2060<br>• *Total no. of amputations:* 27

<table>
<tr><td></td><td>Setting:
Location: Thailand, South-East Asia Region
Study setting and level: Hospital based - Multiple</td></tr>
<tr><td>[65]</td><td>Title: Thailand Diabetes Registry Project: Prevalence of Vascular Complications in Long-standing Type 2 Diabetes
Authors: Leelawattana R, Pratipanawatr T, Bunnag P, et al.
• Publication Year: 2006
• Study Design: Prospective Cross-Sectional
• Study Period: April 2003 - December 2003
• JBI Quality Score: Moderate (6)
Population:
The study population comprised adult patients (>15 years) with Type 2 diabetes registered in the nationwide Diabetes Registry Project in Thailand.
• Mean Age (SD): 65.5 (10.3)
• Male, %(n): 33.95% (3152)
Outcome:
The study explored vascular complications among Thai Type 2 persons with DM, comparing those with DM duration over 15 years to those with a shorter duration.
• Population at risk: 9284
• Total no. of amputations: 408
Setting:
Location: Thailand, South-East Asia Region
Study setting and level: Hospital based - Tertiary level</td></tr>
<tr><td>[66]</td><td>Title: Outcomes of hospitalized diabetic foot patients in a multi-disciplinary team setting: Thailand's experience
Authors: Y. Thewjitcharoen, S. Krittiyawong, S. Porramatikul, et. al.
• Publication Year: 2014
• Study Design: Retrospective Cohort
• Study Period: 2009 - 2013
• JBI Quality Score: Moderate (7)
Population:
The study population included all hospitalized persons with diabetes foot treated at Theptarin Hospital (2009–2013).
• Mean Age (SD): 65.6 (11.9)
• Male, %(n): 53.01% (123)
Outcome:
The study evaluated treatment outcomes among hospitalized Thai patients with DFUs, identifying risk factors for non-healing ulcers and determining rates of major amputation.</td></tr>
</table>

<table>
<tr><td></td><td>

- *Population at risk:* 232
- *Total no. of amputations:* 60

Setting:

Location: Thailand, South-East Asia Region

Study setting and level: Hospital based - Not Specified
</td></tr>
<tr><td>[67]</td><td>

Title: Changing the patterns of hospitalized diabetic foot ulcer (DFU) over a 5-year period in a multi-disciplinary setting in Thailand

Authors: Y. Thewjitcharoen, J. Sripatpong, S. Krittiyawong, et. al.

- *Publication Year:* 2020
- *Study Design:* Retrospective Cohort
- *Study Period:* 2014 - 2018
- *JBI Quality Score:* Moderate (7)

Population:

The study population included consecutive hospitalized admissions for DFU.

- *Mean Age (Range):* 65.5 (13.3)
- *Male, %(n):* 57.58% (167)

Outcome:

The study examined recent trends and outcomes in hospitalized DFU patients at a tertiary diabetes centre in Bangkok, focusing on amputation, healing, and mortality rates.

- *Population at risk:* 290
- *Total no. of amputations:* 94

Setting:

Location: Thailand, South-East Asia Region

Study setting and level: Hospital based - Tertiary level
</td></tr>
<tr><td>[68]</td><td>

Title: The Influence of an Interdisciplinary Diabetic Foot Team on the Outcome of Patients with Diabetic Foot

Authors: Ömer Coşkun, Günalp Uzun, Ahmet Karakaş, et al.

- *Publication Year:* 2016
- *Study Design:* Retrospective Cohort
- *Study Period:* January 2005 - October 2014
- *JBI Quality Score:* Moderate (7)

Population:

The study population consisted of patients hospitalized for DFI in the department of infectious diseases.

- *Mean Age (SD):* ~63 (+-9.5)
- *Male, %(n):* 72.82% (67)

Outcome:
</td></tr>
</table>

	The study examined the effect of an interdisciplinary team approach on patient characteristics, hospitalization length, and amputation rates among patients admitted for DFI.<br>• *Population at risk:* 92<br>• *Total no. of amputations:* 36<br>**Setting:**<br>*Location:* Turkey, South-East Asia Region<br>*Study setting and level:* Hospital based - Not Specified
[69]	**Title:** Prevalence, risk level and risk factors of diabetic foot ulcer among adult individuals with diabetes in the Southeastern Anatolia Region of Turkiye<br>**Authors:** M. Kilic, N. Olgun, M. Dündar, et. al.<br>• *Publication Year:* 2024<br>• *Study Design:* Prospective Cross-Sectional<br>• *Study Period:* 1 June 2022 - 31 November 2023<br>• *JBI Quality Score:* Moderate (6)<br>**Population:**<br>The study population included adults with diabetes attending diabetes/internal medicine outpatient clinics in selected hospitals across 7 cities; excluded stroke, and bilateral below-knee amputation.<br>• *Mean Age (SD):* 60.22 (13.5)<br>• *Male, %(n):* 42.02% (150)<br>**Outcome:**<br>The study determined the prevalence and risk factors for DFU among adults with diabetes, identifying predisposing factors.<br>• *Population at risk:* 357<br>• *Total no. of amputations:* 8<br>**Setting:**<br>*Location:* Turkey, South-East Asia Region<br>*Study setting and level:* Hospital based - Multiple
[70]	**Title:** Change in amputation predictors in diabetic foot disease: Effect of multidisciplinary approach<br>**Authors:** K. Aydin, M. Isildak, J. Karakaya, et al<br>• *Publication Year:* 2010<br>• *Study Design:* Retrospective Cohort<br>• *Study Period:* 1 January 2002 - 31 December 2007<br>• *JBI Quality Score:* Moderate (7)<br>**Population:**

	The study population included patients with diabetic foot disease hospitalized at Hacettepe University Hospital (Jan 1, 2002–Dec 31, 2007); incomplete records excluded.<br>• *Mean Age (SD):* 62.4 (10.6)<br>• *Male, %(n):* 70.27% (52)<br>**Outcome:**<br>The study investigated changes in amputation rates and their predictors in persons with DFI managed through a multidisciplinary team approach, comparing outcomes with two previous hospital data sets from 1992–1996 and 2000–2002.<br>• *Population at risk:* 74<br>• *Total no. of amputations:* 16<br>**Setting:**<br>*Location:* Turkey, South-East Asia Region<br>*Study setting and level:* Hospital based - Teaching Hospital
[71]	**Title:** Prediction of amputation risk of patients with diabetic foot using classification algorithms: A clinical study from a tertiary center<br>**Authors:** D. Demirkol, C. Erol, X. Tannier, et al.<br>• *Publication Year:* 2024<br>• *Study Design:* Retrospective Cohort<br>• *Study Period:* 1 January 2009 - 31 September 2019<br>• *JBI Quality Score:* Moderate (7)<br>**Population:**<br>The study population included patients with DFU (n=407) with known amputations.<br>• *Mean Age:* Not reported<br>• *Male, %(n):* Not reported<br>**Outcome:**<br>The study used machine-learning classification algorithms to predict amputation risk among persons with diabetic foot.<br>• *Population at risk:* 407<br>• *Total no. of amputations:* 179<br>**Setting:**<br>*Location:* Turkey, South-East Asia Region<br>*Study setting and level:* Hospital based - Tertiary level
[72]	**Title:** Reliability and Validity of the Perfusion, Extent, Depth, Infection and Sensation (PEDIS) Classification System and Score in Patients with Diabetic Foot Ulcer<br>**Authors:** F. Chuan, K. Tang, P. Jiang, et. al.<br>• *Publication Year:* 2015<br>• *Study Design:* Retrospective Cohort

	• *Study Period:* May 2007 - September 2013<br>• *JBI Quality Score:* High (9)<br>**Population:**<br>The study population comprised patients with Type 1 or Type 2 DM who had at least one DFU treated at a specialized tertiary diabetic foot care centre, excluding those with non-diabetic foot conditions, secondary diabetes, or ulcers caused by autoimmune disease, malignancy, or acute foot ischemia.<br>• *Mean Age (Range):* 66 (23-95)<br>• *Male, %(n):* 60.16% (219)<br>**Outcome:**<br>The study aimed to validate the PEDIS classification system by creating and comparing a new scoring method with two existing ones to simplify clinical practice.<br>• *Population at risk:* 364<br>• *Total no. of amputations:* 62<br>**Setting:**<br>*Location:* China, Western Pacific Region<br>*Study setting and level:* Hospital based - Tertiary level
[73]	**Title:** Factors associated with lower-extremity amputation in patients with diabetic foot ulcers in a Chinese tertiary care hospital<br>**Authors:** Guo Z, Yue C, Qian Q., et al.<br>• *Publication Year:* 2019<br>• *Study Design:* Retrospective Cohort<br>• *Study Period:* December 2014 - September 2018<br>• *JBI Quality Score:* High (9)<br>**Population:**<br>The study population included patients with DFUs referred to the Third Xiangya Hospital between December 2014 and September 2018.<br>• *Mean Age (SD):* 61.18 (12.01)<br>• *Male, %(n):* 61.89% (294)<br>**Outcome:**<br>The study examined clinical differences between DFU patients who underwent amputation and those who did not in Hunan, China, identifying independent risk factors such as elevated HbA1c, lower triglycerides, and higher Wagner grades.<br>• *Population at risk:* 475<br>• *Total no. of amputations:* 59<br>**Setting:**<br>*Location:* China, Western Pacific Region<br>*Study setting and level:* Hospital based - Tertiary level

[74]	**Title:** Epidemiology of Type 2 Diabetic Foot Problems and Predictive Factors for Amputation in China<br>**Authors:** Y. Jiang, X. Ran, L. Jia, et. al.<br>• *Publication Year:* 2015<br>• *Study Design:* Prospective Cohort<br>• *Study Period:* 1 February 2012 - 31 January 2013<br>• *JBI Quality Score:* High (9)<br>**Population:**<br>The study population comprised patients with Type 2 DM and DFUs admitted to diabetic centres across 15 hospitals during the study period.<br>• *Mean Age (Range):* 64 (55.8-72.0)<br>• *Male, %(n):* 65.02% (435)<br>**Outcome:**<br>The study determined the incidence and key clinical risk factors for diabetic amputations—such as WBC count, infection, and diabetes duration—among DFU patients in China.<br>• *Population at risk:* 669<br>• *Total no. of amputations:* 113<br>**Setting:**<br>*Location:* China, Western Pacific Region<br>*Study setting and level:* Country – Multiple Levels
[75]	**Title:** Reducing major lower extremity amputations after the introduction of a multidisciplinary team in patients with diabetes foot ulcer<br>**Authors:** C. Wang, L. Mai, C. Yang<br>• *Publication Year:* 2016<br>• *Study Design:* Retrospective Cohort<br>• *Study Period:* 2004 - 2013<br>• *JBI Quality Score:* High (9)<br>**Population:**<br>The study population included patients with DFUs attending Sun Yat-sen Memorial Hospital with Wagner grade 1–5 ulcers.<br>• *Mean Age (SD):* 66.96 (+-11.96)<br>• *Male, %(n):* 51.54% (334)<br>**Outcome:**<br>The study compared the frequency of LEA s in DFU patients over a ten-year period.<br>• *Population at risk:* 648<br>• *Total no. of amputations:* 78<br>**Setting:**<br>*Location:* China, Western Pacific Region

	Study setting and level: Hospital based - Tertiary level
[76]	**Title:** Relationship of Diabetic Retinopathy with Severe Diabetic Foot and Diabetic Foot Amputation<br><br>**Authors:** J. An, L. Lu, G. An, et. al.<br><br>• *Publication Year:* 2019<br>• *Study Design:* Retrospective Cohort<br>• *Study Period:* December 2015 - December 2018<br>• *JBI Quality Score:* Moderate (7)<br><br>**Population:**<br><br>The study population included DM patients meeting 1999 WHO criteria and DF meeting 1999 WHO IWGDF criteria; excluded specified ocular/optic/uveitic/retinal conditions, prior eye surgery/PRP, non-DM vascular/ulcer aetiologies, acute Type 2 DM complications, and incomplete data.<br><br>• *Mean Age: Not reported*<br>• *Male, %(n):* 63.54% (61)<br><br>**Outcome:**<br><br>The study analysed the relationship between diabetic retinopathy and severe diabetic foot as well as DF-related amputations.<br><br>• *Population at risk:* 96<br>• *Total no. of amputations:* 22<br><br>**Setting:**<br><br>*Location:* China, Western Pacific Region<br><br>*Study setting and level:* Hospital based - Tertiary level
[77]	**Title:** Age Characteristics of Patients With Type 2 Diabetic Foot Ulcers and Predictive Risk Factors for Lower Limb Amputation: A Population-Based Retrospective Study<br><br>**Authors:** Yuanying Yao, Lei Chen, Yu Qian<br><br>• *Publication Year:* 2024<br>• *Study Design:* Retrospective Cohort<br>• *Study Period:* 1 January 2017 - 31 December 2023<br>• *JBI Quality Score:* Moderate (8)<br><br>**Population:**<br><br>The study population included adults >18 diagnosed with Type 2 DFU identified via ICD; excluded serious mental illness and non-healing refractory wounds due to prior trauma/foot surgery; records lacking critical data also excluded.<br><br>• *Mean Age (SD):* 61.86 (12.1)<br>• *Male, %(n):* 68.19% (626)<br><br>**Outcome:**<br><br>The study investigated the age-related characteristics of Type 2 DFU patients and analysed key factors predicting lower-extremity and major amputations.

	• *Population at risk:* 918<br>• *Total no. of amputations:* 178<br>**Setting:**<br>*Location:* China, Western Pacific Region<br>*Study setting and level:* Country – Multiple Levels
[78]	**Title:** Neutrophil Extracellular Traps (NETs) Are Associated with Type 2 Diabetes and Diabetic Foot Ulcer Related Amputation: A Prospective Cohort Study<br>**Authors:** I. Ibrahim, Y. Nuermaimaiti, G. Maimaituxun, et. al.<br>• *Publication Year:* 2024<br>• *Study Design:* Prospective Cohort<br>• *Study Period:* 1 June 2019 - 31 June 2022<br>• *JBI Quality Score:* Moderate (8)<br>**Population:**<br>The study population included three cohorts: Type 2 DM with DFU (n=200), newly diagnosed Type 2 DM (n=42), and healthy donors (n=38), meeting IWGDF or Chinese diabetic foot guidelines; excluded venous ulceration, trauma-related lower extremity ulcers, acute vascular occlusion, and skin tumours.<br>• *Mean Age (Range):* 62 (56-66)<br>• *Male, %(n):* 52.5% (105)<br>**Outcome:**<br>The study investigated serum NET levels in Type 2 DFU patients to evaluate novel biomarkers for clinical application and their prognostic value in disease outcomes.<br>• *Population at risk:* 200<br>• *Total no. of amputations:* 52<br>**Setting:**<br>*Location:* China, Western Pacific Region<br>*Study setting and level:* Hospital based - Tertiary level
[79]	**Title:** Development of a major amputation prediction model and nomogram in patients with diabetic foot<br>**Authors:** Y. Chen, J. Zhuang, C. Yang<br>• *Publication Year:* 2024<br>• *Study Design:* Retrospective Cohort<br>• *Study Period:* 1 January 2018 - 31 December 2023<br>• *JBI Quality Score:* Moderate (8)<br>**Population:**<br>The study population included patients with Type 2 DM and DFU meeting WHO-1999 and IWGDF-2015 criteria, age >18, and consented; excluded Type 1 or gestational DM, malignant tumours, and incomplete/inaccurate case data.<br>• *Mean Age (SD):* 61.64 (11.27)

	• *Male, %(n):* 73.81% (468)<br>**Outcome:**<br>The study developed a predictive nomogram model to estimate the risk of major amputation in patients with diabetic foot.<br>• *Population at risk:* 634<br>• *Total no. of amputations:* 71<br>**Setting:**<br>*Location:* China, Western Pacific Region<br>*Study setting and level:* Hospital based - Tertiary level
[80]	**Title:** Analysis of clinical characteristics in patients with diabetic foot ulcers undergoing amputation and establishment of a nomogram prediction model<br>**Authors:** T. Xu, L. Hu, B. Xie, et al.<br>• *Publication Year:* 2024<br>• *Study Design:* Retrospective Cohort<br>• *Study Period:* 1 January 2020 - 31 December 2023<br>• *JBI Quality Score:* Moderate (6)<br>**Population:**<br>The study population included DFU patients (n=144) at the First Affiliated Hospital of Nanchang University meeting IWGDF-2015 criteria with complete case data; excluded age <18, non-DFU infections, malignant tumour, or prior LLA.<br>• *Mean Age (SD):* 60.43 (12.01)<br>• *Male, %(n):* 74.30% (107)<br>**Outcome:**<br>The study assessed the accuracy of a nomogram for predicting the risk of LEA in individuals with DFUs.<br>• *Population at risk:* 144<br>• *Total no. of amputations:* 72<br>**Setting:**<br>*Location:* China, Western Pacific Region<br>*Study setting and level:* Hospital based - Tertiary level
[81]	**Title:** Predictive Factors of Major Lower Extremity Amputations in Diabetic Foot Infections: A Cross-sectional Study at District Hospital in Malaysia<br>**Authors:** RY. Kow, CL. Low, JK. Ruben<br>• *Publication Year:* 2019<br>• *Study Design:* Retrospective Cross-Sectional<br>• *Study Period:* 1 September 2014 - 31 December 2015<br>• *JBI Quality Score:* Moderate (7)

	**Population:**<br>The study population comprised patients who underwent surgical interventions for DFIs at three district hospitals between 1 September 2014 and 31 December 2015, excluding those with incomplete data.<br>• *Mean Age: Not reported*<br>• *Male, %(n):* 51.17% (87)<br>**Outcome:**<br>The study determined predictive factors of major LLA among patients with DM at three district hospitals in Pahang, Malaysia.<br>• *Population at risk:* 170<br>• *Total no. of amputations:* 21<br>**Setting:**<br>*Location:* Malaysia, Western Pacific Region<br>*Study setting and level:* Hospital based – Primary Level
**Incidence Studies**	
[82]	**Title:** Chronic neuropathic ulcer is not the most common antecedent of lower limb infection or amputation among diabetics admitted to a regional hospital in Jamaica: results from a prospective cohort study<br>**Authors:** J. M. East, D. A. Fray, D. E. Hall, et. al.<br>• *Publication Year:* 2015<br>• *Study Design:* Prospective Cohort<br>• *Study Period:* 16 March 2011 - 25 March 2013<br>• *JBI Quality Score:* High (9)<br>**Population:**<br>The study population included adult patients (≥18 years) with DM admitted to the hospital with a primary diagnosis of lower limb bacterial infection and/or gangrene, excluding those with persistent cognitive impairment during admission.<br>• *Mean Age (Range):* 58.4 (28-94)<br>• *Male, %(n):* 35.93% (46)<br>**Outcome:**<br>The study determined the frequency of antecedent events leading to serious lower-limb infection, gangrene, and amputation among adult persons with diabetes admitted to a Regional Hospital in western Jamaica.<br>• *Population at risk:* 128<br>• *Total no. of amputations:* 75<br>**Setting:**<br>*Location:* Jamaica, Region of the Americas<br>*Study setting and level:* Hospital based - Tertiary level

[83]	**Title:** The analysis of the cost and amputation rates of hospitalized diabetic foot infection patients<br>**Authors:** G. Karagöz, A. Kadanalı, S. Öztürk, et al.<br>• *Publication Year:* 2016<br>• *Study Design:* Retrospective Cohort<br>• *Study Period:* 1 January 2012 - 31 December 2015<br>• *JBI Quality Score:* Moderate (7)<br>**Population:**<br>The study population comprised adult persons with diabetes hospitalized with third- or fourth-category DFIs, including those with deep or extensive local infections per the PEDIS classification, excluding patients with other comorbid conditions requiring hospitalization such as nephropathy or diabetic acidosis.<br>• *Mean Age (Range):* 61.6 (32-85)<br>• *Male, %(n):* 71.73% (99)<br>**Outcome:**<br>The study analysed trends in direct hospital costs and amputation rates among patients hospitalized for DFI over time.<br>• *Population at risk:* 138<br>• *Total no. of amputations:* 80<br>**Setting:**<br>*Location:* Turkey, South-East Asia Region<br>*Study setting and level:* Hospital based - Teaching Hospital
[84]	**Title:** Prognostic Factors for Lower Extremity Amputation in<br>Diabetic Foot Ulcer Patients<br>**Authors:** A. Kurniawati, Y.D. Ismiarto, I. Hsu<br>• *Publication Year:* 2019<br>• *Study Design:* Retrospective Cohort<br>• *Study Period:* January 2017 - December 2017<br>• *JBI Quality Score:* Moderate (7)<br>**Population:**<br>The study population included patients with Type 2 DM and DFUs of Wagner grade 2 treated conservatively, excluding those younger than 30 years or with fasting blood glucose >200 mg/dL, serum albumin <2.5 g/dL, or haemoglobin <10 g/dL.<br>• *Mean Age:* 47<br>• *Male, %(n):* 13.69% (10)<br>**Outcome:**<br>The study investigated predictive risk factors for amputation among persons with diabetes with foot ulcers, measuring the incidence of LLA.<br>• *Population at risk:* 73

	• *Total no. of amputations:* 14<br>**Setting:**<br>*Location:* Indonesia, South-East Asia Region<br>*Study setting and level:* Hospital based - Tertiary level
[85]	**Title:** The Guyana Diabetes and Foot Care Project: Improved Diabetic Foot Evaluation Reduces Amputation Rates by Two-Thirds in a Lower Middle Income Country<br>**Authors:** J. Lowe, R. G. Sibbald, N. Y. Taha, et al.<br>• *Publication Year:* 2015<br>• *Study Design:* Prospective Cohort<br>• *Study Period:* July 2010 - March 2013<br>• *JBI Quality Score:* Moderate (6)<br>**Population:**<br>The study population included persons with Type 2 DM presenting to the Georgetown Public Hospital Corporation or regional facilities with personnel trained in the project.<br>• *Mean Age (SD):* Not reported<br>• *Male, %(n):* 66.77% (5053)<br>**Outcome:**<br>The study aimed to improve foot care and reduce diabetes-related amputations at the national referral hospital and regional centres, measuring reductions in major LEAs and improvements in diabetes care.<br>• *Population at risk:* 7567<br>• *Total no. of amputations:* 544<br>**Setting:**<br>*Location:* Guyana, Region of the Americas<br>*Study setting and level:* Multiple locations - Tertiary level
[86]	**Title:** An Assessment of the Disease Burden of Foot Ulcers in Patients With Diabetes Mellitus Attending a Teaching Hospital in Lagos, Nigeria<br>**Authors:** A. Ogbera, O. Fasanmade, A. Ohwovoriole, et al.<br>• *Publication Year:* 2006<br>• *Study Design:* Retrospective Cross-Sectional<br>• *Study Period:* 1998 - 2000<br>• *JBI Quality Score:* Moderate (7)<br>**Population:**<br>The study population included patients with DFUs and diabetes.<br>• *Mean Age (Range):* 57.9 (32-78)<br>• *Male, %(n):* 64.8% (81)<br>**Outcome:**

<table>
<tr><td></td><td>The study determined the disease burden and morbidity outcomes associated with DFUs as a major complication of DM.
• Population at risk: 125
• Total no. of amputations: 61
Setting:
Location: Nigeria, African Region
Study setting and level: Hospital based - Teaching Hospital</td></tr>
<tr><td>[87]</td><td>Title: Comparison of three systems of classification in predicting the outcome of diabetic foot ulcers in a Brazilian population
Authors: MCR. Parisi, DE. Zantut-Wittmann, EJ. Pavin, et. al
• Publication Year: 2008
• Study Design: Prospective Cohort
• Study Period: 1 December 2003 - 31 December 2005
• JBI Quality Score: High (9)
Population:
The study population comprised patients with DFUs treated at a specialist clinic in Brazil.
• Mean Age (SD): 57.6 (+-12.44)
• Male, %(n): 54.28% (57)
Outcome:
The study compared the Wagner, University of Texas, and ulcer classification systems as predictors of outcomes in DFUs at a specialist clinic in Brazil.
• Population at risk: 94
• Total no. of amputations: 11
Setting:
Location: Brazil, Region of the Americas
Study setting and level: Hospital based - Teaching Hospital</td></tr>
<tr><td>[88]</td><td>Title: Presentation and outcome of diabetic foot at a tertiary care unit
Authors: S. M. Ali, A. Basit, A. Fawwad, et. al.
• Publication Year: 2008
• Study Design: Prospective Cohort
• Study Period: January 1997 - December 2003
• JBI Quality Score: Moderate (7)
Population:
The study population comprised persons with diabetes with foot ulceration attending the Baqai Institute of Diabetology and Endocrinology, excluding those lost to follow-up or unable to complete treatment.
• Mean Age (SD): 53.64 (10.38)
• Male, %(n): 63.55% (136)</td></tr>
</table>

	**Outcome:**<br>The study defined the presentation and risk factors influencing outcomes of diabetic foot ulceration leading to LEA in a tertiary hospital in Karachi, Pakistan.<br>• *Population at risk:* 214<br>• *Total no. of amputations:* 59<br>**Setting:**<br>*Location:* Pakistan, Eastern Mediterranean Region<br>*Study setting and level:* Hospital based - Tertiary level
[89]	**Title:** Incidence and determinants of diabetes related lower limb amputations in Ghana, 2010–2015- a retrospective cohort study<br>**Authors:** O. Sarfo-Kantanka, F. Sarfo, I. Kyei, et. al.<br>• *Publication Year:* 2019<br>• *Study Design:* Retrospective Cohort<br>• *Study Period:* 1 January 2010 - 31 December 2015<br>• *JBI Quality Score:* Moderate (8)<br>**Population:**<br>The study population included patients enrolled in the diabetes clinic at Komfo Anokye Teaching Hospital (2010–2015) who underwent diabetes-related LLA; excluded if DFU at enrolment, prior diabetes-related LLA, lost to follow-up, or died before 1 year of follow-up.<br>• *Mean Age (SD):* 55.9 (14.6)<br>• *Male, %(n):* 37.89% (1191)<br>**Outcome:**<br>The study determined the annual incidence of diabetes-related LLAs in an outpatient tertiary clinic from 2010 to 2015 and identified clinical predictors of these amputations.<br>• *Population at risk:* 3143<br>• *Total no. of amputations:* 78<br>**Setting:**<br>*Location:* Ghana, African Region<br>*Study setting and level:* Hospital based - Tertiary level
[90]	**Title:** Association of multidrug-resistant bacteria and clinical outcomes in patients with infected diabetic foot in a Peruvian hospital: A retrospective cohort analysis<br>**Authors:** M. Yovera-Aldana, P. Sifuentes-Hermenegildo, M.S. Cervera-Ocaña, et al.<br>• *Publication Year:* 2024<br>• *Study Design:* Retrospective Cohort<br>• *Study Period:* 1 January 2017 - 31 December 2019<br>• *JBI Quality Score:* Moderate (8)<br>**Population:**

	The study population included patients treated in a General Hospital Diabetic Foot Unit with DFI, excluded absence of bacterial culture.<br>• *Mean Age (SD):* 59.9 (12.9)<br>• *Male, %(n):* 73.95% (142)<br>**Outcome:**<br>The study evaluated the association between multidrug-resistant bacteria and adverse clinical outcomes in patients with DFI at a Peruvian hospital.<br>• *Population at risk:* 192<br>• *Total no. of amputations:* 34<br>**Setting:**<br>*Location:* Peru, Region of the Americas<br>*Study setting and level:* Hospital based - Secondary level
[91]	**Title:** Machine Learning Algorithm-Aided Determination of Predictors of Mortality from Diabetic Foot Sepsis at a Regional Hospital in South Africa During the COVID-19 Pandemic<br>**Authors:** C. Matsinhe, S.B. Kagodora, T. Mukheli, et al.<br>• *Publication Year:* 2024<br>• *Study Design:* Retrospective Cohort<br>• *Study Period:* 1 March 2020 - 30 October 2021<br>• *JBI Quality Score:* Moderate (8)<br>**Population:**<br>The study population included patients admitted and treated for diabetic foot sepsis at a regional hospital in Ekurhuleni District, Gauteng.<br>• *Mean Age (SD):* 55 (14)<br>• *Male, %(n):* 57.89% (66)<br>**Outcome:**<br>The study determined factors associated with major amputation and mortality among patients admitted with diabetic foot syndrome during the COVID-19 pandemic.<br>• *Population at risk:* 114<br>• *Total no. of amputations:* 79<br>**Setting:**<br>*Location:* South Africa, African Region<br>*Study setting and level:* Hospital based - Secondary level
[92]	**Title:** Predictors of amputation in patients with diabetic foot ulcers: a multi-centre retrospective cohort study<br>**Authors:** D. Che, Z. Jiang, X. Xiang, et al.<br>• *Publication Year:* 2024<br>• *Study Design:* Retrospective Cohort<br>• *Study Period:* 1 January 2020 - 30 June 2023

	• *JBI Quality Score:* Moderate (8)<br>**Population:**<br>The study population included in-hospital DFU cohort of 526 patients across five Anhui hospitals (Jan 1, 2020–Jun 30, 2023) with DF diagnosis at admission and discharge and available clinical data; excluded stays ≤3 days, >30% data missing, concurrent malignancy/immunosuppression, or in-hospital death.<br>• *Mean Age (SD):* 63.32 (12.14)<br>• *Male, %(n):* 65.96% (347)<br>**Outcome:**<br>The study aimed to identify and describe risk factors associated with amputation among DFU patients in Anhui Province, China.<br>• *Population at risk:* 526<br>• *Total no. of amputations:* 264<br>**Setting:**<br>*Location:* China, Western Pacific Region<br>*Study setting and level:* Hospital based – Multiple levels
[93]	**Title:** Incidence and Risk Factors for Amputations in Persons with Diabetes Mellitus: A Retrospective Cohort Study<br>**Authors:** M. Bandeira, R. Macedo, J. Lázaro-Martinez, et al.<br>• *Publication Year:* 2025<br>• *Study Design:* Retrospective Cohort<br>• *Study Period:* 2015 - 2020<br>• *JBI Quality Score:* Moderate (8)<br>**Population:**<br>The study population included patients ≥18 with Type 1 or 2 DM treated 2015–2020, excluded amputations unrelated to diabetes or insufficient records.<br>• *Mean Age (SD):* 65.6 (13.05)<br>• *Male, %(n):* 87.55% (211)<br>**Outcome:**<br>The study identified and analysed the incidence and risk factors for amputations among people with DM attending two specialized outpatient clinics in Brazil.<br>• *Population at risk:* 241<br>• *Total no. of amputations:* 77<br>**Setting:**<br>*Location:* Brazil, Region of the Americas<br>*Study setting and level:* Community based - Primary level
[94]	**Title:** Initial handgrip strength as a predictive factor for amputation risk in diabetic foot patients<br>**Authors:** E. Imre, E. Imre, S. Ozturk

<table>
<tr><td></td><td>

- *Publication Year:* 2024
- *Study Design:* Prospective Cohort
- *Study Period:* 1 August 2020 - 31 June 2022
- *JBI Quality Score:* Moderate (8)

Population:

The study population included outpatients (Aug 2020–Jun 2023) with Type 2 DM and diabetic foot disease; excluded Type 1 diabetes, cancer, steroid use, neurological/mobility issues, haemodialysis, or missing handgrip data.

- *Mean Age (SD):* 60.67 (10.78)
- *Male, %(n):* 63.41% (130)

Outcome:

The study analysed the prognostic significance of handgrip strength as a predictor of LEA within one year of follow-up in patients with Type 2 DM.

- *Population at risk:* 205
- *Total no. of amputations:* 77

Setting:

Location: Turkey, South-East Asia Region

Study setting and level: Hospital based - Tertiary level
</td></tr>
<tr><td>[95]</td><td>

Title: Relationship between systemic immune inflammation index and amputation in patients with diabetic foot ulcer

Authors: M. Aydin, M. Eren, N. Uyar, et al.

- *Publication Year:* 2023
- *Study Design:* Retrospective Cohort
- *Study Period:* 2018 - 2021
- *JBI Quality Score:* Moderate (8)

Population:

The study population included in-patients with DFU (2017–2021); excluded Type 1 DM, severe hepatic or end-stage renal failure, known malignancy, rheumatologic disease, pregnancy, or another infection.

- *Mean Age (SD):* 61.3 (10.38)
- *Male, %(n):* 57.14% (292)

Outcome:

The study investigated the relationship between the systemic immune-inflammation index and amputation rates in DFU patients.

- *Population at risk:* 511
- *Total no. of amputations:* 96

Setting:

Location: Turkey, South-East Asia Region

Study setting and level: Hospital based - Tertiary level
</td></tr>
</table>

[96]	**Title:** Predictive Value of C-Reactive Protein to Albumin Ratio for Amputation Risk in Diabetic Foot Infection<br><br>**Authors:** B. Karaca, B. Ormen, T. Kiris, et. al.<br><br>• *Publication Year:* 2025<br>• *Study Design:* Retrospective Cohort<br>• *Study Period:* 1 January 2019 - 31 January 2020<br>• *JBI Quality Score:* Moderate (8)<br><br>**Population:**<br><br>The study population included all patients with DFIs.<br><br>• *Mean Age (SD):* 62.9 (11.4)<br>• *Male, %(n):* 66.29% (118)<br><br>**Outcome:**<br><br>The study examined the predictive value of the C-reactive protein to albumin ratio for assessing amputation risk in patients with DFI.<br><br>• *Population at risk:* 178<br>• *Total no. of amputations:* 65<br><br>**Setting:**<br><br>*Location:* Turkey, South-East Asia Region<br><br>*Study setting and level:* Hospital based - Tertiary level
[97]	**Title:** An interpreting machine learning models to predict amputation<br><br>risk in patients with diabetic foot ulcers: a multi-center study<br><br>**Authors:** H.Tao, L. You, Y. Huang et. al.<br><br>• *Publication Year:* 2025<br>• *Study Design:* Retrospective Cohort<br>• *Study Period:* 1 January 2020 - 31 October 2023<br>• *JBI Quality Score:* Moderate (8)<br><br>**Population:**<br><br>The study population included all DFU patients (Wagner 1–5) admitted to the Endocrinology Department at Sun Yat-sen Memorial Hospital with confirmed Type 2 DM and age >18; excluded non-Type 2 DM, refusal/discontinuation, malignancy, or major data deficiencies/ambiguous outcomes.<br><br>• *Mean Age (SD):* 66.2 (12.2)<br>• *Male, %(n):* 60.96% (631)<br><br>**Outcome:**<br><br>The study developed machine-learning models to predict the risk of LEA in DFU patients and used SHapley additive explanations to interpret the models.<br><br>• *Population at risk:* 1035<br>• *Total no. of amputations:* 472<br><br>**Setting:**

	Location: China, Western Pacific Region<br>*Study setting and level:* Hospital based - Tertiary level
[98]	**Title:** Risk factors and outcomes of diabetic foot ulcer among diabetes mellitus patients admitted to Nekemte referral hospital, western Ethiopia: Prospective observational study<br>**Authors:** F. Bekelea, L. Chelkebab, G. Fekaduc, et al.<br>• *Publication Year:* 2020<br>• *Study Design:* Prospective Cohort<br>• *Study Period:* 15 March 2018 - 15 June 2018<br>• *JBI Quality Score:* Moderate (7)<br>**Population:**<br>The study population included patients ≥18 admitted with non-traumatic chronic DFU with visible foot lesions; excluded traumatic ulcers from accidents/burns/sharp injuries and those in acute stress.<br>• *Mean Age (SD):* 44.4 (14.7)<br>• *Male, %(n):* 55.65% (64)<br>**Outcome:**<br>The study identified factors associated with the amputation rate among DFU patients treated at Nekemte Referral Hospital.<br>• *Population at risk:* 115<br>• *Total no. of amputations:* 35<br>**Setting:**<br>*Location:* Ethiopia, African Region<br>*Study setting and level:* Hospital based - Tertiary level
[99]	**Title:** Traditional and non-traditional risk factors for peripheral artery disease development/progression in patients with Type 2 diabetes mellitus: the Rio de Janeiro Type 2 diabetes mellitus cohort study<br>**Authors:** CRL Cardoso, JV Melo, TRM Santos.<br>• *Publication Year:* 2021<br>• *Study Design:* Prospective Cohort<br>• *Study Period:* August 2004 - December 2019<br>• *JBI Quality Score:* Moderate (6)<br>**Population:**<br>The study population included Type 2 DM outpatients with any micro/macrovascular complications or ≥2 other modifiable cardiovascular risk factors; excluded BMI ≥40, advanced renal failure (Cr >180 µmol/L or eGFR <30), or serious concomitant disease limiting life expectancy.<br>• *Mean Age (SD):* 59.9 (9.6)<br>• *Male, %(n):* 39.73% (298)<br>**Outcome:**

<table>
<tr><td></td><td>The study investigated whether carotid intima-media thickness and carotid-femoral pulse wave velocity provide additional prognostic information for peripheral arterial disease outcomes in patients with Type 2 DM.
• Population at risk: 750
• Total no. of amputations: 20
Setting:
Location: Brazil, Region of the Americas
Study setting and level: Hospital based - Tertiary level</td></tr>
<tr><td>[100]</td><td>Title: Lower Limb Amputation Rates in Patients With Diabetes and an Infected Foot Ulcer: A Prospective Observational Study
Authors: N. Chaudhary, F. Huda, R. Roshan, et. al.
• Publication Year: 2021
• Study Design: Prospective Cohort
• Study Period: December 2018 - May 2020
• JBI Quality Score: Moderate (6)
Population:
The study population included patients aged 18–65 with diabetes and foot ulcers showing clinical signs of infection; excluded osteomyelitis and conditions restricting mobility (bedridden or wheelchair-bound).
• Mean Age (SD): 54.58 (9.04)
• Male, %(n): 75.30% (61)
Outcome:
The study examined how demographic, vascular, biochemical, microbiological, and radiological variables, including the PEDIS score, influence the rate of LEA in patients with diabetes and infected foot ulcers.
• Population at risk: 81
• Total no. of amputations: 41
Setting:
Location: India, South-East Asia Region
Study setting and level: Hospital based - Teaching Hospital</td></tr>
<tr><td>[101]</td><td>Title: Mortality in Asian Indians with Charcot’s neuroarthropathy: a nested cohort prospective study
Authors: S. Chaudhary, A. Bhansali, A. Rastogi
• Publication Year: 2019
• Study Design: Prospective Cohort
• Study Period: January 2013 - June 2018
• JBI Quality Score: High (9)
Population:</td></tr>
</table>

<table>
<tr><td></td><td>The study population included individuals with diabetes and Charcot's neuropathy at a diabetic foot clinic; missing data excluded.
• Mean Age (SD): 55 (9.07)
• Male, %(n): 69.61% (181)
Outcome:
The study evaluated mortality outcomes among individuals with diabetes with or without Charcot neuroarthropathy of the foot.
• Population at risk: 260
• Total no. of amputations: 40
Setting:
Location: India, South-East Asia Region
Study setting and level: Hospital based - Tertiary level</td></tr>
<tr><td colspan="2">Prevalence and Incidence Studies</td></tr>
<tr><td>[102]</td><td>Title: Patient-level predictors of diabetes-related lower extremity amputations at a quaternary hospital in South Africa
Authors: S. Mtshali, O. Mahomed
• Publication Year: 2020
• Study Design: Retrospective Cohort
• Study Period: 1 January 2014 - 31 December 2015
• JBI Quality Score: High (10)
Population:
The study population included adult (≥18 years) diabetes patients treated at Inkosi Albert Luthuli Central Hospital during the study period, excluding mothers with gestational diabetes.
• Mean Age (SD): 60.95 (10.49)
• Male, %(n): 41% (95)
Outcome:
The study determined the incidence and patient-related factors associated with diabetes-related amputations among patients referred to a quaternary hospital between January 2014 and December 2015.
• Population at risk: 13495
• Total no. of amputations: 333 (99 Prevalence and 234 Incidence)
Setting:
Location: South Africa, African Region
Study setting and level: Hospital based – Quaternary</td></tr>
<tr><td colspan="2">Prevalence and Incidence of Re-amputation Studies</td></tr>
<tr><td>[103]</td><td>Title: Differences in initial versus recurrent diabetic foot ulcers at a specialized tertiary diabetic foot care center in China
Authors: Yuxia Cheng, Ping Zu, Jie Zhao, et al.</td></tr>
</table>

	Publication Year: 2021 *Study Design:* Prospective Cohort *Study Period:* March 2014 - October 2018 *JBI Quality Score:* High (9) **Population:**<br>The study population included patients with DFUs who were treated at a specialized tertiary diabetic foot care center, excluding those with non-diabetic foot conditions. *Mean Age (SD):* 61 (53.70) *Male, %(n):* 67.01% (384) **Outcome:**<br>The study investigated the characteristics of DFU recurrence and measured the incidence of amputation. *Population at risk:* 573 *Total no. of amputations:* 75 *Reamputation:* 66/75 **Setting:**<br>*Location:* China, Western Pacific Region<br>*Study setting and level:* Hospital based - Specialist
[104]	**Title:** Predictive factors for major amputation of lower limb in diabetic foot: about 430 patients<br>**Authors:** M. Khalfallah, E. L. Gouta, W. Dougaz, et al. *Publication Year:* 2018 *Study Design:* Prospective Cohort *Study Period:* 1 June 2008 – 31 December 2011 *JBI Quality Score:* Moderate (6) **Population:**<br>The study population comprised all patients admitted with an infected diabetic foot to the Surgery Unit B of Charles Nicolle Hospital in Tunis, regardless of age, sex, medical history, emergency status, or type of surgery performed. *Mean Age (SD):* 60.5 (12) *Male, %(n):* 74.18% (319) **Outcome:**<br>The study identified predictive factors for major LLA in persons with diabetes admitted with foot lesions using an administrative database. *Population at risk:* 430 *Total no. of amputations:* 396 *Reamputation:* 37/396 **Setting:**<br>*Location:* Tunisia, Eastern Mediterranean Region<br>*Study setting and level:* Hospital based – Teaching Hospital

[105]	**Title:** Predisposing factors for lower limb amputation in diabetic patients hospitalized with ulcerated feet in the state of Sergipe<br>**Authors:** MA. Nunes, KF. Resende, AA. Castro, et al.<br>• *Publication Year:* 2006<br>• *Study Design:* Prospective Cross-Sectional<br>• *Study Period:* July – December 2004<br>• *JBI Quality Score:* High (10)<br>**Population:**<br>The study population comprised persons with DFU, excluding those who died during hospitalization, pregnant women, or individuals unable to provide informed consent.<br>• *Mean Age (SD):* 62 (11.58)<br>• *Male, %(n):* 58.75% (47)<br>**Outcome:**<br>The study identified predisposing factors for LLA in hospitalized persons with DFU, measuring whether amputation occurred during hospitalization.<br>• *Population at risk:* 80<br>• *Total no. of amputations:* 44<br>• *Reamputation:* 13/18<br>**Setting:**<br>*Location:* Brazil, Region of the Americas<br>*Study setting and level:* Hospital based – Level not specified
[106]	**Title:** Clinical profile and outcome of diabetic foot ulcer, a view from tertiary care hospital in Semarang, Indonesia<br>**Authors:** TGD. Pemayuna and RM. Naibaho<br>• *Publication Year:* 2017<br>• *Study Design:* Retrospective Cross-Sectional<br>• *Study Period:* January 2012 – December 2014<br>• *JBI Quality Score:* High (9)<br>**Population:**<br>The study population included persons with diabetes admitted to Dr. Kariadi General Hospital with foot problems, excluding those with incomplete data or who declined amputation.<br>• *Mean Age (SD):* 54.3 (8.6)<br>• *Male, %(n):* 50.79% (96)<br>**Outcome:**<br>The study assessed the disease burden, clinical profile, and outcomes of DFU admissions at a tertiary care hospital in a developing country.<br>• *Population at risk:* 189<br>• *Total no. of amputations:* 98<br>• *Reamputation:* 7/98<br>**Setting:**

	Location: Indonesia, South-East Asia Region<br>*Study setting and level:* Hospital based – Teaching Hospital
[107]	**Title:** Predictors for limb loss among patients with diabetic foot infections: an observational retrospective multicentric study in Turkey<br>**Authors:** N. Saltoglu, M. Yemisen, O. Ergonul, et al.<br>• *Publication Year:* 2015<br>• *Study Design:* Retrospective Cohort<br>• *Study Period:* May 2011 – May 2013<br>• *JBI Quality Score:* High (9)<br>**Population:**<br>The study population included consecutive patients with DFI followed at 17 Turkish centres (May 2011–May 2013), inpatients and outpatients.<br>• *Mean Age (Range):* 62 (57–70)<br>• *Male, %(n):* 67.91% (309)<br>**Outcome:**<br>The study investigated predictors of limb loss among persons with diabetes with complicated skin and soft-tissue infections, with limb loss as the primary outcome.<br>• *Population at risk:* 455<br>• *Total no. of amputations:* 126<br>• *Reamputation:* 32/126<br>**Setting:**<br>*Location:* Turkey, South-East Asia Region<br>*Study setting and level:* Multiple centres with multiple levels
[108]	**Title:** Surgical management of diabetic foot ulcers: A Tanzanian university teaching hospital experience<br>**Authors:** PL. Chalya, JB. Mabula, RM. Dass, et al.<br>• *Publication Year:* 2011<br>• *Study Design:* Prospective Cohort<br>• *Study Period:* February 2008 – January 2010<br>• *JBI Quality Score:* Moderate (7)<br>**Population:**<br>The study population included all patients presenting to surgical wards or the surgical outpatient clinic with DFUs who consented; healed ulcers were excluded.<br>• *Mean Age (SD):* 54.32 (16.24)<br>• *Male, %(n):* 54.55% (2312)<br>**Outcome:**<br>The study described the experience of surgical management of DFUs in a local setting and compared outcome characteristics—including hospital stay, mortality, and amputation rate—with existing literature.<br>• *Population at risk:* 4238<br>• *Total no. of amputations:* 72<br>• *Reamputation:* 6/72

	**Setting:**<br>*Location:* Tanzania, African Region<br>*Study setting and level:* Multiple locations – Teaching Hospital
**Prevalence and Incidence of Contralateral Amputation Studies**	
[109]	**Title:** Surgical management of diabetic foot ulcers: A Tanzanian university teaching hospital experience<br>**Authors:** Jokotade O. Adeleye, SR Adeleye, BA Solagberu<br>• *Publication Year:* 2004<br>• *Study Design:* Retrospective Cohort<br>• *Study Period:* September 1999 – December 2003<br>• *JBI Quality Score:* Moderate (7)<br>**Population:**<br>The study population included patients with diabetic foot disease hospitalized at the University College Hospital during the study period, excluding those whose case notes could not be found.<br>• *Mean Age (SD):* 59.5 (11.6)<br>• *Male, %(n):* 63.63% (35)<br>**Outcome:**<br>The study reviewed outcomes and challenges of diabetic foot disease among hospitalized patients in Nigeria and measured rates of major and minor amputations, mortality, and associations with socioeconomic status.<br>• *Population at risk:* 55<br>• *Total no. of amputations:* 17<br>• *Reamputation:* 2/17<br>**Setting:**<br>*Location:* Nigeria, African Region<br>*Study setting and level:* Hospital based - Tertiary level
**Incidence of Re-amputation Studies**	
[110]	**Title:** Association of risk factors with a major re-amputation in Malaysian diabetic patients: a retrospective cohort analysis of patient registry<br>**Authors:** A. A. S. Abdullah, I. Ismail, M. A. Bujang<br>• *Publication Year:* 2016<br>• *Study Design:* Retrospective Cohort<br>• *Study Period:* June 2008 - December 2009<br>• *JBI Quality Score:* Moderate (6)<br>**Population:**<br>The study population included persons with diabetes admitted to one of 18 Malaysian hospitals (Jun 2008–Dec 2009) with complications of feet or hands and a history of amputation.<br>• *Mean Age (SD):* 56.9 (8.7)<br>• *Male, %(n):* 50.36% (69)

	**Outcome:**<br>The study determined associations between various risk factors and the likelihood of major re-amputation among Malaysian patients with diabetes.<br>• *Reamputation:* 32/137<br>**Setting:**<br>*Location:* Malaysia, Western Pacific Region<br>*Study setting and level:* Country based – Multiple levels
**Incidence, Re-amputation and Contralateral Amputation Studies**	
[111]	**Title:** Diabetic foot ulcers, their characteristics, and trends in survival: Real world outcomes at a tertiary care facility in India<br>**Authors:** Zachariah Thomas, Shrirang Kishor Bhurchandi, Bharathi Saravanan, et al.<br>• *Publication Year:* 2024<br>• *Study Design:* Retrospective Cohort<br>• *Study Period:* 2011 - 2018<br>• *JBI Quality Score:* Moderate (8)<br>**Population:**<br>The study population included two hundred DM patients with plantar foot ulcers attending the Integrated Diabetes Foot Clinic at CMC Vellore (2011–2018) with ≥3 visits; excluded bilateral ulcers at presentation, stump ulcers, other neuropathy causes, dorsal ulcers, and non-diabetic ulcer aetiologies.<br>• *Mean Age (SD):* 62.29 (10)<br>• *Male, %(n):* 63.5% (127)<br>**Outcome:**<br>The study characterized the clinical profile of patients with DM and foot ulcers at a tertiary care facility in Southern India, describing associations with ulcer recurrence and amputation.<br>• *Population at risk:* 200<br>• *Total no. of amputations:* 53<br>• *Reamputation:* 28/122<br>• *Contralateral amputation:* 16/53<br>**Setting:**<br>*Location:* India, South-East Asia Region<br>*Study setting and level:* Hospital based - Primary level

References:

[1] Kengne AP, Djouogo CF, Dehayem MY, Fezeu L, Sobngwi E, Lekoubou A, et al. Admission trends over 8 years for diabetic foot ulceration in a specialized diabetes unit in cameroon. Int J Low Extrem Wounds. 2009;8(4):180–6. https://doi.org/10.1177/1534734609349704

[2] Brown CK, Kejeh C, Limnyuy C, Mboni L, Ngansi T, Nguesseh B, et al. Assessing the knowledge of patients with diabetes about foot care and prevention of foot complications in Cameroon, West Africa. Wounds. 2025;37(2):51–5. https://doi.org/10.25270/wnds/24160

[3] Atosona A, Larbie C. Prevalence and Determinants of Diabetic Foot Ulcers and Lower Extremity Amputations in Three Selected Tertiary Hospitals in Ghana. J Diabetes Res. 2019;2019:7132861. https://doi.org/10.1155/2019/7132861

[4] Adigun IO, J. Foot complications in people with diabetes_Experience with 105 Nigerian Africans. Diabetic Foot Journal. 2008;11(1):36–42.

[5] Enamino M, Odoh G, Uwakwe JN, Puepet F. Predictors of lower extremity amputation among patients with diabetic foot ulcer in a tertiary health facility in North Central Nigeria. Nigerian Journal of Medicine. 2018;27:92. https://doi.org/10.4103/1115-2613.278236

[6] Musa AA. Diabetic foot lesions as seen in Nigerian teaching hospital: pattern and a simple classification. East Afr J Public Health. 2012;9(1):50–2. PMID: 23120950

[7] Ugwu E, Adeleye O, Gezawa I, Okpe I, Enamino M, Ezeani I. Predictors of lower extremity amputation in patients with diabetic foot ulcer: findings from MEDFUN, a multi-center observational study. J Foot Ankle Res. 2019;12:34. https://doi.org/10.1186/s13047-019-0345-y

[8] Mané DI, Demba D, Djiby S, Assane NM, Limane BA, Marie KC, et al. [Clinical and progressive profile of skin and soft tissue lesions in diabetics in 2017 at the dressing room

of the Marc Sankale Center in Dakar]. Pan Afr Med J. 2019;32:209. https://doi.org/10.11604/pamj.2019.32.209.18524

[9] Levitt NS, Bradshaw D, Zwarenstein MF, Bawa AA, Maphumolo S. Audit of public sector primary diabetes care in Cape Town, South Africa: high prevalence of complications, uncontrolled hyperglycaemia, and hypertension. Diabet Med. 1997;14(12):1073–7. https://doi.org/10.1002/(SICI)1096-9136(199712)14:12%3C1073::AID-DIA498%3E3.0.CO;2-9

[10] Gulam-Abbas Z, Lutale JK, Morbach S, Archibald LK. Clinical outcome of diabetes patients hospitalized with foot ulcers, Dar es Salaam, Tanzania. Diabet Med. 2002;19(7):575–9. https://doi.org/10.1046/j.1464-5491.2002.00740.x

[11] Assaad-Khalil SH, Zaki A, Abdel Rehim A, Megallaa MH, Gaber N, Gamal H, et al. Prevalence of diabetic foot disorders and related risk factors among Egyptian subjects with diabetes. Prim Care Diabetes. 2015;9(4):297–303. https://doi.org/10.1016/j.pcd.2014.10.010

[12] Saad N, Elhadedy K, Ramadan N, Mohmady O, Farid M. The prevalence and risk categorization of diabetic foot complications in cohort group in, Beni Suif, Egypt, 2010-2012. Life Science Journal. 2013;10:933–42.

[13] Niakan MH, Dehghankhalili M, Pourdavood A, Vahidi A, Nazemi S, Piranviseh A, et al. Clinical outcome and determinants of amputation in a large cohort of Iranian patients with diabetic foot ulcers. Foot (Edinb). 2020;45:101688. https://doi.org/10.1016/j.foot.2020.101688

[14] Hadadi A, Omdeh Ghiasi H, Hajiabdolbaghi M, Zandekarimi M, Hamidian R. Diabetic foot: infections and outcomes in Iranian admitted patients. Jundishapur J Microbiol. 2014;7(7):e11680. https://doi.org/10.5812/jjm.11680

[15] Janmohammadi N, Hasanjani Roshan MR, Rouhi M, Esmailnejad Ganji SM, Bahrami M, Moazezi Z. Management of diabetic foot ulcer in Babol, North of Iran: an experience on 520 cases. Caspian J Intern Med. 2012;3(3):456–9. PMID: 24009914

[16] Akha O, Kashi Z, Makhlough A. Correlation between amputation of diabetic foot and nephropathy. Iran J Kidney Dis. 2010;4(1):27–31. PMID: 20081301

[17] Khalooei A, Meymand M. Frequency of Diabetes Foot Ulcer and Related Factors Among Adult Diabetic Patients in the Diabetes Center of Kerman, Iran. Shiraz E-Medical Journal. 2019; In Press. https://doi.org/10.5812/semj.90082

[18] Alikhani A, Moradi M, Alikhani S. Epidemiological study, risk factors and clinical outcomes of diabetic foot infection in hospitalized patients. Journal of Mazandaran University of Medical Sciences. 2015;25:81–91.

[19] Shojaiefard A, Khorgami Z, Larijani B. Septic diabetic foot is not necessarily an indication for amputation. J Foot Ankle Surg. 2008;47(5):419–23. https://doi.org/10.1053/j.jfas.2008.05.005

[20] Aalaa M, Vahdani AM, Mohajeri Tehrani M, Mehrdad N, Zohdirad M, Sadati M, et al. Epidemiological Insights into Diabetic Foot Amputation and its Correlates: A Provincial Study. Clin Med Insights Endocrinol Diabetes. 2024;17. https://doi.org/10.1177/11795514241227618

[21] Mansour A. Chronic Complications of Diabetes in Iraq: Experience from Southern Iraq. Clinical Medicine: Endocrinology and Diabetes. 2010;2:1–9. https://doi.org/10.4137/CMED.S3657

[22] Mansour A, Imran H. Foot abnormalities in diabetics: Prevalence & predictors in Basrah, Iraq. Pakistan Journal of Medical Sciences. 2006;22.

[23] AlAyed MY, Younes N, Al-Smady M, Khader YS, Robert AA, Ajlouni K. Prevalence of Foot Ulcers, Foot at Risk and Associated Risk Factors Among Jordanian Diabetics. Curr Diabetes Rev. 2017;13(2):182–91. https://doi.org/10.2174/1573399812666151210143140

[24] Jbour AS, Jarrah NS, Radaideh AM, Shegem NS, Bader IM, Batieha AM, et al. Prevalence and predictors of diabetic foot syndrome in type 2 diabetes mellitus in Jordan. Saudi Med J. 2003;24(7):761–4. PMID: 12883610

[25] Benkhadoura M, Alswehly M, Elbarsha A. Clinical profile and surgical management of diabetic foot in Benghazi, Libya. Foot Ankle Surg. 2016;22(1):55–8. https://doi.org/10.1016/j.fas.2015.04.011

[26] Naqvi IH, Talib A, Naqvi SH, Yasin L, Rizvi NZ. The Neuro-Vascular Consequence of Diabetes: Foot Amputation and Evaluation of its Risk Factors and Health-Related Economic Impact. Curr Vasc Pharmacol. 2021;19(1):102–9. https://doi.org/10.2174/1570161118666200320112401

[27] Riaz M, Miyan Z, Waris N, Zaidi SIH, Tahir B, Fawwad A, et al. Impact of multidisciplinary foot care team on outcome of diabetic foot ulcer in term of lower extremity amputation at a tertiary care unit in Karachi, Pakistan. Int Wound J. 2019;16(3):768–72. https://doi.org/10.1111/iwj.13095

[28] Shafi A, Maaz Ul H, Tufail S. A study of risk factors of diabetic foot ulcers. Medical Forum Monthly. 2018;29:64–6.

[29] Gul A, Basit A, Ali SM, Ahmadani MY, Miyan Z. Role of wound classification in predicting the outcome of diabetic foot ulcer. J Pak Med Assoc. 2006;56(10):444–7. PMID: 17144390

[30] Imran S, Ali R, Mahboob G. Frequency of lower extremity amputation in diabetics with reference to glycemic control and Wagner's grades. J Coll Physicians Surg Pak. 2006;16(2):124–7. PMID: 16499806

[31] Rehman R, Malik F, Rehman Z. A comparative study on diabetic foot ulcers leading to amputations. Journal of Postgraduate Medical Institute. 2018;32:40–3.

[32] Riaz M, Miyan Z, Zaidi SI, Alvi SF, Fawwad A, Ahmadani MY, et al. Characteristics of a large cohort of patients with diabetes having at-risk feet and outcomes in patients with foot ulceration referred to a tertiary care diabetes unit. Int Wound J. 2016;13(5):594–9. https://doi.org/10.1111/iwj.12289

[33] Elkhider ATE, Almobark AO, Badi S, Tahir H, Ramadan A, Khalil AA, et al. Risk factors associated with lower extremity amputation in Sudanese individuals with diabetes: The need for improvement in primary health care system. J Family Med Prim Care. 2021;10(2):985–90. https://doi.org/10.4103/jfmpc.jfmpc_1881_20

[34] Bakheit HE, Mohamed MF, Mahadi SE, Widatalla AB, Shawer MA, Khamis AH, et al. Diabetic heel ulcer in the Sudan: determinants of outcome. J Foot Ankle Surg. 2012;51(2):152–5. https://doi.org/10.1053/j.jfas.2011.10.032

[35] Aziz ARA, Alsabek MB. Diabetic foot and disaster; risk factors for amputation during the Syrian crisis. J Diabetes Complications. 2020;34(2):107493. https://doi.org/10.1016/j.jdiacomp.2019.107493

[36] Tanirbergenova A, Kamaliev M, Akanov Z, Igissenova A. Analysis of Disability due to Diabetes Mellitus in a Large City. Electronic Journal of General Medicine. 2020;18:em271. https://doi.org/10.29333/ejgm/9347

[37] Uysal S, Arda B, Taşbakan MI, Çetinkalp Ş, Şimşir IY, Öztürk AM, et al. Risk factors for amputation in patients with diabetic foot infection: a prospective study. Int Wound J. 2017;14(6):1219–24. https://doi.org/10.1111/iwj.12788

[38] Spinelli MB, Cafruni VM, Lucero Viviani N, Parise A, Rodriguez C, Satera N, et al. [Translated article] Prevalence of diabetic foot in patients with diabetes mellitus undergoing

dialysis treatment in a tertiary-level hospital in Argentina. Revista Española de Cirugía Ortopédica y Traumatología. 2025;70.

[39] Costa W, Penha-Silva N, Bezerra I, Santos I, Ramos J, Castro J, et al. Analysis of Diabetes Mellitus-Related Amputations in the State of Espírito Santo, Brazil. Medicina. 2020;56:287. https://doi.org/10.3390/medicina56060287

[40] Mantovani AM, Fregonesi CE, Palma MR, Ribeiro FE, Fernandes RA, Christofaro DG. Relationship between amputation and risk factors in individuals with diabetes mellitus: A study with Brazilian patients. Diabetes Metab Syndr. 2017;11(1):47–50. https://doi.org/10.1016/j.dsx.2016.08.002

[41] Moura Neto A, Zantut-Wittmann DE, Fernandes TD, Nery M, Parisi MC. Risk factors for ulceration and amputation in diabetic foot: study in a cohort of 496 patients. Endocrine. 2013;44(1):119–24. https://doi.org/10.1007/s12020-012-9829-2

[42] Parisi MC, Moura Neto A, Menezes FH, Gomes MB, Teixeira RM, de Oliveira JE, et al. Baseline characteristics and risk factors for ulcer, amputation and severe neuropathy in diabetic foot at risk: the BRAZUPA study. Diabetol Metab Syndr. 2016;8:25. https://doi.org/10.1186/s13098-016-0126-8

[43] Quilici MT, Del Fiol Fde S, Vieira AE, Toledo MI. Risk Factors for Foot Amputation in Patients Hospitalized for Diabetic Foot Infection. J Diabetes Res. 2016;2016:8931508. https://doi.org/10.1155/2016/8931508

[44] Vieira-Santos ICR, Souza WVd, Carvalho EMFd, Medeiros MCWCd, Nóbrega MGdL, Lima PMS. Prevalência de pé diabético e fatores associados nas unidades de saúde da família da cidade do Recife, Pernambuco, Brasil, em 2005. Cadernos De Saude Publica. 2008;24:2861–70.

[45] Carla Luiza da Silva CM, Emanoel Severo, Maria Dagmar da Rocha Gaspar, Mariane Antunes Cavalheiro. Characteristics of diabetic foot injuries and their complications. Rev Rene [Internet]. 2012;13(2):445–53.

[46] Marques AD, Silva M, Moreira TM, Torres R. Enfermería Global Association between hospitalization due to diabetes mellitus and diabetic foot amputation Associação entre internação hospitalar por diabetes mellitus e amputação de pé diabético Asociación entre hospitalización por diabetes mellitus y amputación de pie diabético. Enfermeria Global. 2018;51. https://doi.org/10.6018/eglobal.17.3.286181

[47] Isabel Cristina Ramos Vieira Santos EFdC, Wayner Vieira de Souza, Emídio Cavalcanti de Albuquerque. Factors associated with diabetic foot amputations. Brazilian Journal of Vascular Surgery. 2015;14(1). https://doi.org/10.1590/1677-5449.20140049.

[48] Santos IC, Sobreira CM, Nunes EN, Morais MC. [The prevalence and factors associated with diabetic foot amputations]. Cien Saude Colet. 2013;18(10):3007–14. https://doi.org/10.1590/s1413-81232013001000025

[49] Diniz I, Oliveira P, Santos I, Oliveira Matos S, Costa I, Costa M, et al. Fatores associados à amputação não traumática em pessoas com Diabetes Mellitus: um estudo transversal. Revista Eletrônica de Enfermagem. 2019;21. https://doi.org/10.5216/ree.v21.52484

[50] Bonilla GA, Hornsby PP, Pannone AF, Case SK, Aviles ES, Carrasco Apolinario ME, et al. Demographic and clinical characteristics of dominican adults admitted to a diabetic foot clinic in the Dominican Republic, 2015. Diabetes Metab Syndr. 2019;13(3):1727–32. https://doi.org/10.1016/j.dsx.2019.03.034

[51] Cisneros-González N, Ascencio-Montiel IJ, Libreros-Bango VN, Rodríguez-Vázquez H, Campos-Hernández Á, Dávila-Torres J, et al. [Lower extremity amputation rates in diabetic patients]. Rev Med Inst Mex Seguro Soc. 2016;54(4):472–9. PMID: 27197105

[52] Latif ZA, Jain A, Rahman MM. Evaluation of management, control, complications and psychosocial aspects of diabetics in Bangladesh: DiabCare Bangladesh 2008. Bangladesh Med Res Counc Bull. 2011;37(1):11–6. https://doi.org/10.3329/bmrcb.v37i1.7793

[53] Rastogi A, Goyal G, Kesavan R, Bal A, Kumar H, Mangalanadanam, et al. Long term outcomes after incident diabetic foot ulcer: Multicenter large cohort prospective study (EDI-FOCUS investigators) epidemiology of diabetic foot complications study: Epidemiology of diabetic foot complications study. Diabetes Res Clin Pract. 2020;162:108113. https://doi.org/10.1016/j.diabres.2020.108113

[54] Zargar AHB, M. H.; Wani, A. I.; Bashir, M. I.; Laway, B. A.; Masoodi, S. R. Diabetic foot: Clinical aspect and therapeutic outcome. Journal International Medical Sciences Academy 2005;18(4):175–6. PMID: 27704116

[55] Mehra BR, Thawait AP, Karandikar SS, Gupta DO, Narang RR. Evaluation of foot problems among diabetics in rural population. Indian J Surg. 2008;70(4):175–80. https://doi.org/10.1007/s12262-008-0048-2

[56] Muduli IC, P. P A, Panda C, Behera NC. Diabetic Foot Ulcer Complications and Its Management—a Medical College-Based Descriptive Study in Odisha, an Eastern State of India. Indian Journal of Surgery. 2015;77(2):270–4. https://doi.org/10.1007/s12262-012-0791-2.

[57] Zubair M, Ahmad J. Transcutaneous oxygen pressure (TcPO2) and ulcer outcome in diabetic patients: Is there any correlation? Diabetes & Metabolic Syndrome: Clinical Research & Reviews. 2019;13(2):953–8. https://doi.org/10.1016/j.dsx.2018.12.008

[58] Zubair M, Malik A, Ahmad J. Incidence, risk factors for amputation among patients with diabetic foot ulcer in a North Indian tertiary care hospital. The Foot. 2012;22(1):24–30. https://doi.org/10.1016/j.foot.2011.09.003

[59] Mondal S, Lodh M, Sahoo S, Paul K, Biswas D, Krishna C, et al. Prevalence and predictors of infected diabetic foot ulcers (DFU) and DFU-related osteomyelitis amongst industrial workers wearing occupational safety footwear. Scientific Reports. 2025;15(1):2576. https://doi.org/10.1038/s41598-025-86013-y

[60] Viswanathan V, Gupta A, Devarajan A, Kumpatla S, Shukla S, Agarwal S, et al. Early screening for foot problems in people with diabetes is the need of the hour: 'Save the Feet and Keep Walking Campaign' in India. BMJ Open Diabetes Research & Care. 2024;12(4):e004064. https://doi.org/10.1136/bmjdrc-2024-004064

[61] Arambewela MH, Somasundaram NP, Jayasekara HBPR, Kumbukage MP, Jayasena PMS, Chandrasekara CMPH, et al. Prevalence of Chronic Complications, Their Risk Factors, and the Cardiovascular Risk Factors among Patients with Type 2 Diabetes Attending the Diabetic Clinic at a Tertiary Care Hospital in Sri Lanka. Journal of Diabetes Research. 2018;2018(1):4504287. https://doi.org/10.1155/2018/4504287

[62] Krittiyawong S, Ngarmukos C, Benjasuratwong Y, Rawdaree P, Leelawatana R, Kosachunhanun N, et al. Thailand diabetes registry project: prevalence and risk factors associated with lower extremity amputation in Thai diabetics. J Med Assoc Thai. 2006;89 Suppl 1:S43–8. PMID: 17715833

[63] Sarinnapakorn V, Sunthorntepwarakul T, Deerochanawong C, Niramitmahapanya S, Napartivaumnuay N. Prevalence of Diabetic Foot Ulcers and Risk Classifications in Type 2 Diabetes Mellitus Patients at Rajavithi Hospital. J Med Assoc Thai. 2016;99 Suppl 2:S99–105. PMID: 27266223

[64] Thai Multicenter Research Group on Diabetes Mellitus. Vascular complications in non-insulin dependent diabetics in Thailand. Diabetes Research and Clinical Practice. 1994;25(1):61–9.

[65] Leelawattana R, Pratipanawatr T, Bunnag P, Kosachunhanun N, Suwanwalaikorn S, Krittiyawong S, et al. Thailand diabetes registry project: prevalence of vascular complications in long-standing type 2 diabetes. J Med Assoc Thai. 2006;89 Suppl 1:S54–9. PMID: 17717878

[66] Thewjitcharoen Y, Krittiyawong S, Porramatikul S, Parksook W, Chatapat L, Watchareejirachot O, et al. Outcomes of hospitalized diabetic foot patients in a multi-disciplinary team setting: Thailand's experience. J Clin Transl Endocrinol. 2014;1(4):187–91. https://doi.org/10.1016/j.jcte.2014.10.002

[67] Thewjitcharoen Y, Sripatpong J, Krittiyawong S, Porramatikul S, Srikummoon T, Mahaudomporn S, et al. Changing the patterns of hospitalized diabetic foot ulcer (DFU) over a 5-year period in a multi-disciplinary setting in Thailand. BMC Endocrine Disorders. 2020;20(1):89. https://doi.org/10.1186/s12902-020-00568-7.

[68] Coşkun ÖU, G.; Karakaş, A.; Tok, D.; Çebi, G.; Çekli, Y.; Yıldız, Ş; Beşirbellioğlu, B. A. The influence of an interdisciplinary diabetic foot team on the outcome of patients with diabetic foot. Journal of Clinical and Analytical Medicine 2016;7(4):529–32.

[69] Kilic M, Olgun N, Dündar M, Celik Advan S, Kücük FZ, Okcuoglu S, et al. Prevalence, risk level and risk factors of diabetic foot ulcer among adult individuals with diabetes in the Southeastern Anatolia Region of Turkiye. Journal of Tissue Viability. 2025;34(1):100839. https://doi.org/10.1016/j.jtv.2024.12.003

[70] Aydin K, Isildak M, Karakaya J, Gürlek A. Change in amputation predictors in diabetic foot disease: effect of multidisciplinary approach. Endocrine. 2010;38(1):87–92. https://doi.org/10.1007/s12020-010-9355-z.

[71] Demirkol D, Erol Ç S, Tannier X, Özcan T, Aktaş Ş. Prediction of amputation risk of patients with diabetic foot using classification algorithms: A clinical study from a tertiary center. Int Wound J. 2024;21(1):e14556. https://doi.org/10.1111/iwj.14556

[72] Chuan F, Tang K, Jiang P, Zhou B, He X. Reliability and Validity of the Perfusion, Extent, Depth, Infection and Sensation (PEDIS) Classification System and Score in Patients with Diabetic Foot Ulcer. PLOS ONE. 2015;10(4):e0124739. https://doi.org/10.1371/journal.pone.0124739

[73] Guo Z, Yue C, Qian Q, He H, Mo Z. Factors associated with lower-extremity amputation in patients with diabetic foot ulcers in a Chinese tertiary care hospital. International Wound Journal. 2019;16(6):1304–13. https://doi.org/10.1111/iwj.13190

[74] Jiang Y, Ran X, Jia L, Yang C, Wang P, Ma J, et al. Epidemiology of Type 2 Diabetic Foot Problems and Predictive Factors for Amputation in China. The International Journal of Lower Extremity Wounds. 2015;14(1):19–27. https://doi.org/10.1177/1534734614564867

[75] Wang C, Mai L, Yang C, Liu D, Sun K, Song W, et al. Reducing major lower extremity amputations after the introduction of a multidisciplinary team in patient with diabetes foot ulcer. BMC Endocrine Disorders. 2016;16(1):38. https://doi.org/10.1186/s12902-016-0111-0

[76] An JL, L.; An, G.; Wang, X.; Yang, Y.; Cui, W. Relationship of Diabetic Retinopathy with Severe Diabetic Foot and Diabetic Foot Amputation. Chinese General Practice. 2019;22(31):3830–5. PMID: 37554103

[77] Yao Y, Chen L, Qian Y. Age Characteristics of Patients With Type 2 Diabetic Foot Ulcers and Predictive Risk Factors for Lower Limb Amputation: A Population-Based Retrospective Study. Journal of Diabetes Research. 2024;2024(1):2380337. https://doi.org/10.1155/jdr/2380337.

[78] Ibrahim I, Nuermaimaiti Y, Maimaituxun G, Luo X, Maimaituxun M, Akbar A, et al. Neutrophil Extracellular Traps (NETs) Are Associated with Type 2 Diabetes and Diabetic Foot Ulcer Related Amputation: A Prospective Cohort Study. Diabetes Ther. 2024;15(6):1333–48. https://doi.org/10.1007/s13300-024-01579-6

[79] Chen Y, Zhuang J, Yang C. Development of a major amputation prediction model and nomogram in patients with diabetic foot. Postgrad Med J. 2024;100(1190):908–16. https://doi.org/10.1093/postmj/qgae087

[80] Xu T, Hu L, Xie B, Huang G, Yu X, Mo F, et al. Analysis of clinical characteristics in patients with diabetic foot ulcers undergoing amputation and establishment of a nomogram prediction model. Sci Rep. 2024;14(1):27934. https://doi.org/10.1038/s41598-024-78215-7

[81] Kow RY, Low CL, Ruben JK, Zaharul-Azri MZ, Lim BC. Predictive Factors of Major Lower Extremity Amputations in Diabetic Foot Infections: A Cross-sectional Study at District Hospital in Malaysia. Malays Orthop J. 2019;13(3):45–52. PMID: 31890110

[82] East JM, Fray DA, Hall DE, Longmore CA. Chronic neuropathic ulcer is not the most common antecedent of lower limb infection or amputation among diabetics admitted to a regional hospital in Jamaica: results from a prospective cohort study. BMC Surgery. 2015;15(1):104. https://doi.org/10.1186/s12893-015-0091-4.

[83] Karagöz G, Kadanalı A, Öztürk S, Öztürk S, Çakar ŞE, Çomoğlu Ş, et al. The analysis of the cost and amputation rates of hospitalized diabetic foot infection patients. International Journal of Diabetes in Developing Countries. 2017;37(2):201–5. https://doi.org/10.1007/s13410-016-0524-3.

[84] Kurniawati A, Ismiarto YD, Hsu IL. Prognostic Factors for Lower Extremity Amputation in Diabetic Foot Ulcer Patients. J Acute Med. 2019;9(2):59–63. https://doi.org/10.6705/j.jacme.201906_9(2).0003

[85] Lowe J, Sibbald RG, Taha NY, Lebovic G, Rambaran M, Martin C, et al. The Guyana Diabetes and Foot Care Project: Improved Diabetic Foot Evaluation Reduces Amputation Rates by Two-Thirds in a Lower Middle Income Country. International Journal of Endocrinology. 2015;2015(1):920124. https://doi.org/10.1155/2015/920124.

[86] Ogbera AO, Fasanmade O, Ohwovoriole AE, Adediran O. An assessment of the disease burden of foot ulcers in patients with diabetes mellitus attending a teaching hospital in Lagos, Nigeria. Int J Low Extrem Wounds. 2006;5(4):244–9. https://doi.org/10.1177/1534734606294538

[87] Parisi MCR, Zantut-Wittmann DE, Pavin EJ, Machado H, Nery M, Jeffcoate WJ. Comparison of three systems of classification in predicting the outcome of diabetic foot ulcers in a Brazilian population. European Journal of Endocrinology. 2008;159(4):417–22. https://doi.org/10.1530/EJE-07-0841

[88] Ali SM, Basit A, Fawwad PDA, Ahmedani MY, Miyan Z, Malik RA, editors. Presentation and outcome of diabetic foot at a tertiary care unit. 2008.

[89] Sarfo-Kantanka O, Sarfo FS, Kyei I, Agyemang C, Mbanya JC. Incidence and determinants of diabetes-related lower limb amputations in Ghana, 2010-2015- a retrospective cohort study. BMC Endocr Disord. 2019;19(1):27. https://doi.org/10.1186/s12902-019-0353-8

[90] Yovera-Aldana M, Sifuentes-Hermenegildo P, Cervera-Ocaña MS, Mezones-Holguin E. Association of multidrug-resistant bacteria and clinical outcomes in patients with infected diabetic foot in a Peruvian hospital: A retrospective cohort analysis. PLOS ONE. 2024;19(6):e0299416. https://doi.org/10.1371/journal.pone.0299416

[91] Matsinhe C, Kagodora SB, Mukheli T, Mokoena TP, Malebati WK, Moeng MS, et al. Machine Learning Algorithm-Aided Determination of Predictors of Mortality from Diabetic Foot Sepsis at a Regional Hospital in South Africa During the COVID-19 Pandemic. Medicina. 2024;60(10):1718. https://doi.org/10.3390/medicina60101718

[92] Che D, Jiang Z, Xiang X, Zhao L, Liu X, Zhou B, et al. Predictors of amputation in patients with diabetic foot ulcers: a multi-centre retrospective cohort study. Endocrine. 2024;85(1):181–9. https://doi.org/10.1007/s12020-024-03704-8

[93] Bandeira MA, Macedo RN, Lázaro-Martinez JL, Alvarez YG, Rocha MNB, Santos VLCdG. Incidence and Risk Factors for Amputations in Persons with Diabetes Mellitus: A Retrospective Cohort Study. The International Journal of Lower Extremity Wounds. 2024;24(1):59–65. https://doi.org/10.1177/15347346241292377

[94] Imre E, Imre E, Ozturk S. Initial handgrip strength as a predictive factor for amputation risk in diabetic foot patients. Rev Clin Esp (Barc). 2024;224(6):346–56. https://doi.org/10.1016/j.rceng.2024.04.011

[95] Aydın MS, Eren MA, Uyar N, Kankılıç N, Karaaslan H, Sabuncu T, et al. Relationship between systemic immune inflammation index and amputation in patients with diabetic foot ulcer. J Orthop Sci. 2024;29(4):1060–3. https://doi.org/10.1016/j.jos.2023.07.015

[96] Karaca B, Ormen B, Kiris T, Sener A. Predictive Value of C-Reactive Protein to Albumin Ratio for Amputation Risk in Diabetic Foot Infection. Angiology. 2025;76(4):391–7. https://doi.org/10.1177/00033197231218330

[97] Tao H, You L, Huang Y, Chen Y, Yan L, Liu D, et al. An interpreting machine learning models to predict amputation risk in patients with diabetic foot ulcers: a multi-center study. Front Endocrinol (Lausanne). 2025;16:1526098. https://doi.org/10.3389/fendo.2025.1526098

[98] Bekele F, Chelkeba L, Fekadu G, Bekele K. Risk factors and outcomes of diabetic foot ulcer among diabetes mellitus patients admitted to Nekemte referral hospital, western Ethiopia: Prospective observational study. Ann Med Surg (Lond). 2020;51:17–23. https://doi.org/10.1016/j.amsu.2020.01.005

[99] Cardoso CRL, Melo JV, Santos TRM, Leite NC, Salles GF. Traditional and non-traditional risk factors for peripheral artery disease development/progression in patients with type 2 diabetes: the Rio de Janeiro type 2 diabetes cohort study. Cardiovascular Diabetology. 2021;20(1):54. https://doi.org/10.1186/s12933-021-01249-y

[100] Chaudhary N, Huda F, Roshan R, Basu S, Rajput D, Singh SK. Lower Limb Amputation Rates in Patients With Diabetes and an Infected Foot Ulcer: A Prospective Observational Study. Wound Manag Prev. 2021;67(7):22–30. PMID: 34264200

[101] Chaudhary S, Bhansali A, Rastogi A. Mortality in Asian Indians with Charcot's neuroarthropathy: a nested cohort prospective study. Acta Diabetol. 2019;56(12):1259–64. https://doi.org/10.1007/s00592-019-01376-9

[102] Mtshali S, Mahomed O. Patient-level predictors of diabetes-related lower extremity amputations at a quaternary hospital in South Africa. PLoS One. 2020;15(10):e0240588. https://doi.org/10.1371/journal.pone.0240588

[103] Cheng Y, Zu P, Zhao J, Shi L, Shi H, Zhang M, et al. Differences in initial versus recurrent diabetic foot ulcers at a specialized tertiary diabetic foot care center in China. J Int Med Res. 2021;49(1):300060520987398. https://doi.org/10.1177/0300060520987398

[104] Khalfallah M, Gouta EL, Dougaz W, Jerraya H, Samaali I, Nouira R, et al. Predictive factors for major amputation of lower limb in diabetic foot: about 430 patients. Tunis Med. 2018;96(5):298–301. PMID: 30430504

[105] Marco Antonio Prado Nunes KFR, Aldemar Araújo Castro, Guilherme Benjamin Brandão Pitta, Luis Francisco Poli de Figueiredo, Fausto Miranda Jr. Fatores predisponentes para amputação de membro inferior em pacientes diabéticos internados com pés ulcerados no estado de Sergipe. Brazilian Journal of Vascular Surgery. 2006. https://doi.org/10.1590/S1677-54492006000200008

[106] Pemayun TGD, Naibaho RM. Clinical profile and outcome of diabetic foot ulcer, a view from tertiary care hospital in Semarang, Indonesia. Diabet Foot Ankle. 2017;8(1):1312974. https://doi.org/10.1080/2000625x.2017.1312974

[107] Saltoglu N, Yemisen M, Ergonul O, Kadanali A, Karagoz G, Baierl A, et al. Predictors for limb loss among patient with diabetic foot infections: an observational retrospective

multicentric study in Turkey. Clin Microbiol Infect. 2015;21(7):659–64. https://doi.org/10.1016/j.cmi.2015.03.018

[108] Chalya PL, Mabula JB, Dass RM, Kabangila R, Jaka H, McHembe MD, et al. Surgical management of Diabetic foot ulcers: A Tanzanian university teaching hospital experience. BMC Res Notes. 2011;4:365. https://doi.org/10.1186/1756-0500-4-365

[109] Adeleye J. Diabetic foot disease: The perspective of a Nigerian tertiary health care centre. Practical Diabetes International. 2005;22:211–4. https://doi.org/10.1002/

[110] Abdullah A, Ismail I, Bujang MA. Association of risk factors with a major re-amputation in Malaysian diabetic patients: a retrospective cohort analysis of patient registry. International Journal of Diabetes in Developing Countries. 2017;38. https://doi.org/10.1007/s13410-017-0551-8

[111] Thomas Z, Bhurchandi SK, Saravanan B, Christina F, Volena R, Rebekah G, et al. Diabetic foot ulcers, their characteristics, and trends in survival: Real world outcomes at a tertiary care facility in India. Diabetes Metab Syndr. 2024;18(4):103011. https://doi.org/10.1016/j.dsx.2024.103011
