# Supplementary material for: Lower Limb Amputations Among Individuals Living With Diabetes Mellitus in Low‐ and Middle‐Income Countries: A Systematic Review and Meta‐Analysis
Source: World J Surg. 2026 Jul 14;50(9):2750–9. doi: 10.1002/wjs.70493 (PMC13576533; doi:10.1002/wjs.70493)
Supplement: Supplementary file 3 [file WJS-50-2750-s003.pdf]

World Journal of Surgery

Title: Lower limb amputations among individuals living with diabetes mellitus in low- and middle-income countries: A systematic review and meta-analysis

Short title: Diabetes-related LLA in LMICs: A systematic review and meta-analysis

Authors

Kriste N. Alberts[a*] (0000-0002-9068-6979), Mercia N. Companie[a] (0009-0007-0015-296X), Maxine P. Kerrod[b] (0009-0003-2801-288X), Jessica Davies[c] (0000-0002-4695-9028), Rosa D.E. Bock[d] (0009-0008-4735-008X), Cinae Swart[b] (0009-0001-7864-7467), Eyitayo O. Owolabi[e, a] (0000-0001-5575-7842), Justine Davies[f] (0000-0001-6834-1838), Kathryn M. Chu[a] (0000-0002-8923-7447), Michael McCaul[g] (0000-0002-2730-6478)

Affiliations

[a] Centre for Global Surgery, Department of Surgery, Stellenbosch University, Stellenbosch, Cape Town, South Africa

[b] Faculty of Medicine and Health Sciences, Stellenbosch University, Stellenbosch, Cape Town, South Africa

[c] International Association of Student Surgical Societies, Cape Town, South Africa

[d] St John's Hospital, NHS Lothian, United Kingdom

[e] Center for Health Promotion and Disease Prevention, Edson College of Nursing and Health Innovation, Arizona State University, Arizona, USA.

[f] Institute of Applied Health Research, University of Birmingham, Birmingham, United Kingdom

[g] Division of Epidemiology and Biostatistics, Department of Global Health, Stellenbosch University, Stellenbosch, Cape Town, South Africa

***Corresponding author**

Kriste N. Alberts

Email address: kristealberts@sun.ac.za

Physical address: Room 4046A, 4th Floor, Education building, Francie van Zijl Drive, Parrow, Cape Town, 7500

Contact number: +27 21 938 9442

Online Resource 1 (ESM_1)

Full electronic search strategies used for the systematic review across Medline, CINAHL, AJOL, Scopus, and Embase, including controlled vocabulary terms, free-text keywords, and Boolean operators related to lower limb amputation, diabetes, and low- and middle-income countries.

1. Medline

#1	Incidence [MeSH Term]
#2	Title, Abstract and Keyword: Proportion OR cumulative incidence OR Incidence rates OR incidence proportion OR Epidemiology OR prevalence
#3	#1 OR #2
#4	Amputations [MeSH Term]
#5	Title, Abstract and Keyword: Lower extremity amputation OR lower limb amputation OR stumps OR prosthesis
#6	#4 OR #5
#7	Diabetes mellitus [MeSH Terms]

#8	Title Abstract Keyword: Diabetes OR type 2 diabetes OR diabetes Mellitus complications OR diabetic complications OR diabetes-related Complication OR diabetic foot
#9	#7 OR #8
#10	Developing countries [MeSH Terms]
#11	Title Abstract Keyword: Afghanistan OR Guinea-Bissau OR Sierra Leone OR Burkina Faso OR Haiti OR Somalia OR Burundi OR Democratic People's Republic of Korea OR South Sudan OR Central African Republic OR Liberia OR Sudan OR Chad OR Madagascar OR Syrian Arab Republic OR Democratic Republic of Congo OR Malawi OR Tajikistan OR Eritrea OR Mali OR Togo OR Ethiopia OR Mozambique OR Uganda OR The Gambia OR Niger OR Republic of Yemen OR Guinea OR Rwanda OR Angola OR Honduras OR Papua New Guinea OR Algeria OR India OR Philippines OR Bangladesh OR Kenya OR São Tomé and Principe OR Benin OR Kiribati OR Senegal OR Bhutan OR Kyrgyz Republic OR Solomon Islands OR Bolivia OR Lao PDR OR Sri Lanka OR Cabo Verde OR Lesotho OR Tanzania OR Cambodia OR Mauritania OR Timor-Leste OR Cameroon OR Federated States of Micronesia OR Tunisia OR Comoros OR Moldova OR Ukraine OR Republic of Congo OR Mongolia OR Uzbekistan OR Côte d'Ivoire OR Morocco OR Vanuatu OR Djibouti OR Myanmar OR Vietnam OR Arab Republic of Egypt OR Nepal OR West Bank and Gaza OR El Salvador OR Nicaragua OR Zambia OR Eswatini OR Nigeria OR Zimbabwe OR Ghana OR Pakistan OR Albania OR Fiji OR Montenegro OR American Samoa OR Gabon OR Namibia OR Argentina OR Georgia OR North Macedonia OR Armenia OR Grenada OR Paraguay OR Azerbaijan OR Guatemala OR Peru OR Belarus OR Guyana OR Russian Federation

	OR Belize OR Indonesia OR Samoa OR Bosnia and Herzegovina OR Islamic Republic of Iran OR Serbia OR Botswana OR Iraq OR South Africa OR Brazil OR Jamaica OR St. Lucia OR Bulgaria OR Jordan OR St. Vincent and the Grenadines OR China OR Kazakhstan OR Suriname OR Colombia OR Kosovo OR Thailand OR Costa Rica OR Lebanon OR Tonga OR Cuba OR Libya OR Turkey OR Dominica OR Malaysia OR Turkmenistan OR Dominican Republic OR Maldives OR Tuvalu OR Equatorial Guinea OR Marshall Islands OR Venezuela OR RB Ecuador OR Mexico
#12	#10 OR #11
#13	#3 AND #6 AND #9 AND #12
	Limit to 1990-2021

2. **CINAHL**

Similar search strategy used for Medline will be applied on this database.

Scopus: These databases do not use Mesh Terms, Keywords similar to those applied on PubMed-Medline will be used on these databases as below:

3. **Scopus and Embase**

#1	(incidence OR proportion OR "cumulative incidence" OR "Incidence rates" OR "incidence proportion" OR epidemiology)
#2	(amputations OR stumps OR prosthesis OR "lower extremity amputation" OR "lower limb amputation")

#3	("Diabetes mellitus" OR diabetes OR "diabetes Mellitus complications" OR "diabetic Complications" OR "diabetes-related Complication" OR "diabetic foot")
#4	("Developing countries" OR Afghanistan OR "Guinea-Bissau" OR "Sierra Leone" OR "Burkina Faso" OR Haiti OR Somalia OR Burundi OR Korea OR "Democratic people's republic of south Sudan" OR "Central African Republic" OR Liberia OR Sudan OR Chad OR Madagascar OR "Syrian Arab Republic" OR "Democratic Republic of Congo" OR Malawi OR Tajikistan OR Eritrea OR Mali OR Togo OR Ethiopia OR Mozambique OR Uganda OR "The Gambia" OR Niger OR "Republic of Yemen" OR Guinea OR Rwanda OR Angola OR Honduras OR "Papua New Guinea" OR Algeria OR India OR Philippines OR Bangladesh OR Kenya OR "Sao tomé and principe" OR Benin OR Kiribati OR Senegal OR Bhutan OR "Kyrgyz Republic" OR "Solomon Islands" OR Bolivia OR "Lao PDR" OR "Sri Lanka" OR "Cabo Verde" OR Lesotho OR Tanzania OR Cambodia OR Mauritania OR "Timor-leste" OR Cameroon OR "Federated states of micronesia" OR Tunisia OR Comoros OR Moldova OR Ukraine OR "Republic of Congo" OR Mongolia OR Uzbekistan OR "Cote d'ivoire" OR Morocco OR Vanuatu OR Djibouti OR Myanmar OR Vietnam OR "Arab republic of Egypt" OR Nepal OR "West bank and Gaza" OR "El salvador" OR Nicaragua OR Zambia OR Eswatini OR Nigeria OR Zimbabwe OR Ghana OR Pakistan OR Albania OR Fiji OR Montenegro OR "American Samoa" OR Gabon OR Namibia OR Argentina OR Georgia OR "North Macedonia" OR Armenia OR Grenada OR Paraguay OR Azerbaijan OR Guatemala OR Peru OR Belarus OR Guyana OR "Russian Federation" OR Belize OR Indonesia OR Samoa OR "Bosnia and Herzegovina" OR "Islamic republic of Iran" OR Serbia OR Botswana OR Iraq OR "South Africa" OR Brazil OR Jamaica OR "St. Lucia" OR Bulgaria OR Jordan OR "St. Vincent and the Grenadines" OR China OR Kazakhstan OR Suriname OR

	Colombia OR Kosovo OR Thailand OR "Costa Rica" OR Lebanon OR Tonga OR Cuba OR Libya OR Turkey OR Dominica OR Malaysia OR Turkmenistan OR "Dominican Republic" OR Maldives OR Tuvalu OR "Equatorial Guinea" OR "Marshall Islands" OR Venezuela OR "Rb Ecuador" OR Mexico).
#5	#1 AND #2 AND #3 AND #4

4. African Journal Online (AJOL)

Keywords used for Scopus database will be applied on AJOL to extract relevant studies.
