# Supplementary material for: Lower Limb Amputations Among Individuals Living With Diabetes Mellitus in Low‐ and Middle‐Income Countries: A Systematic Review and Meta‐Analysis
Source: World J Surg. 2026 Jul 14;50(9):2750–9. doi: 10.1002/wjs.70493 (PMC13576533; doi:10.1002/wjs.70493)
Supplement: Supplementary file 5 [file WJS-50-2750-s005.pdf]

World Journal of Surgery

Title: Lower limb amputations among individuals living with diabetes mellitus in low- and middle-income countries: A systematic review and meta-analysis

Short title: Diabetes-related LLA in LMICs: A systematic review and meta-analysis

Authors

Kriste N. Alberts[a*] (0000-0002-9068-6979), Mercia N. Companie[a] (0009-0007-0015-296X), Maxine P. Kerrod[b] (0009-0003-2801-288X), Jessica Davies[c] (0000-0002-4695-9028), Rosa D.E. Bock[d] (0009-0008-4735-008X), Cinae Swart[b] (0009-0001-7864-7467), Eyitayo O. Owolabi[e, a] (0000-0001-5575-7842), Justine Davies[f] (0000-0001-6834-1838), Kathryn M. Chu[a] (0000-0002-8923-7447), Michael McCaul[g] (0000-0002-2730-6478)

Affiliations

[a] Centre for Global Surgery, Department of Surgery, Stellenbosch University, Stellenbosch, Cape Town, South Africa

[b] Faculty of Medicine and Health Sciences, Stellenbosch University, Stellenbosch, Cape Town, South Africa

[c] International Association of Student Surgical Societies, Cape Town, South Africa

[d] St John's Hospital, NHS Lothian, United Kingdom

[e] Center for Health Promotion and Disease Prevention, Edson College of Nursing and Health Innovation, Arizona State University, Arizona, USA.

[f] Institute of Applied Health Research, University of Birmingham, Birmingham, United Kingdom

[g] Division of Epidemiology and Biostatistics, Department of Global Health, Stellenbosch University, Stellenbosch, Cape Town, South Africa

***Corresponding author**

Kriste N. Alberts

Email address: kristealberts@sun.ac.za

Physical address: Room 4046A, 4th Floor, Education building, Francie van Zijl Drive, Parrow, Cape Town, 7500

Contact number: +27 21 938 9442

Online Resource 5 (ESM_5)

Forest plot showing the individual study estimates and pooled incidence of re-amputation following diabetes-related lower limb amputation among individuals with diabetes in low- and middle-income countries, with corresponding 95% confidence intervals, and heterogeneity statistics.

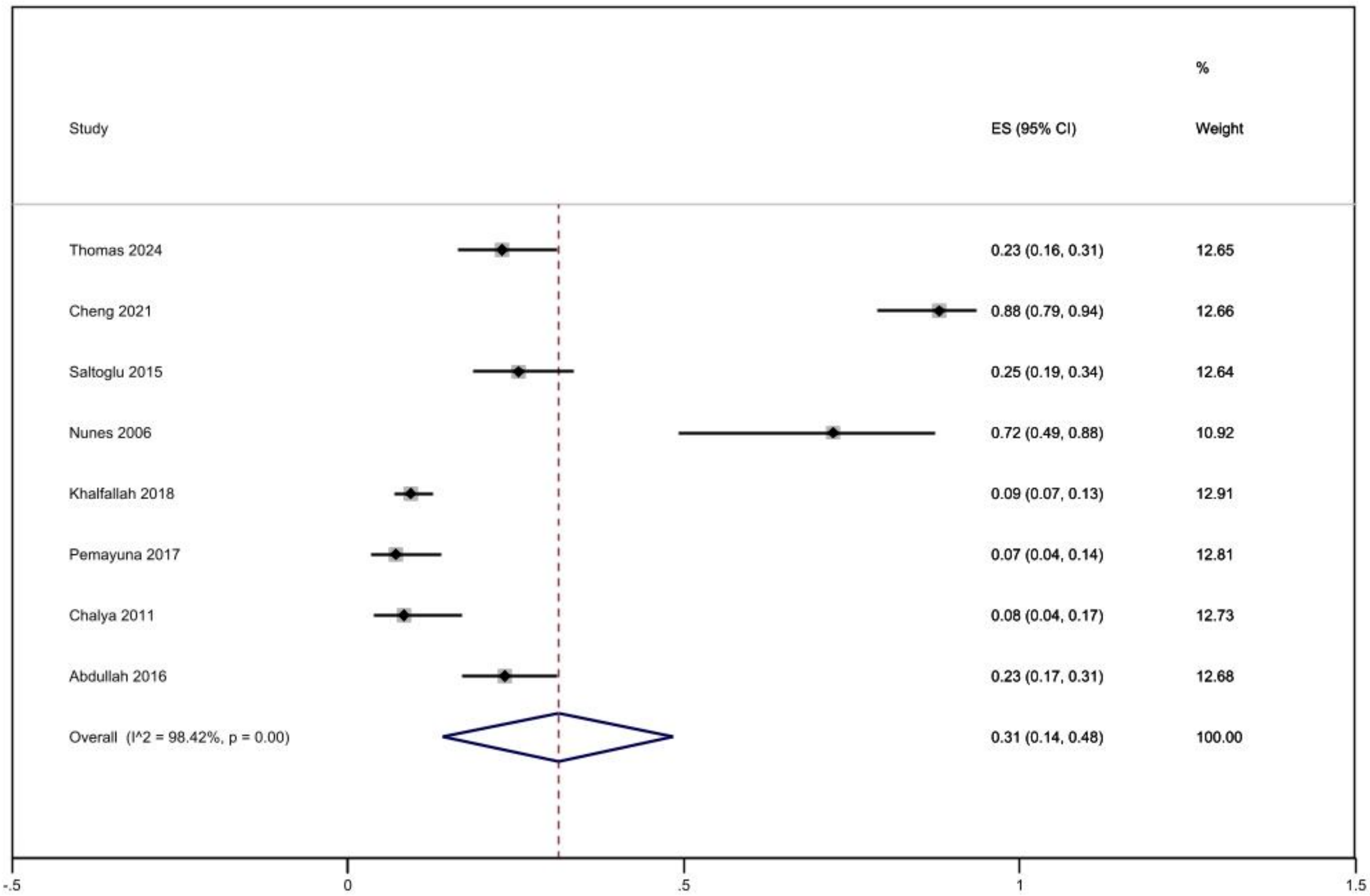

Figure 1: Forest plot showing individual study estimates and pooled incidence of re-amputation among patients with diabetes-related lower-limb amputation with 95% confidence intervals.
